# NSC 18725, a Pyrazole Derivative Inhibits Growth of Intracellular *Mycobacterium tuberculosis* by Induction of Autophagy

**DOI:** 10.3389/fmicb.2019.03051

**Published:** 2020-01-28

**Authors:** Garima Arora, Gagandeep, Assirbad Behura, Tannu Priya Gosain, Ravi P. Shaliwal, Saqib Kidwai, Padam Singh, Shamseer Kulangara Kandi, Rohan Dhiman, Diwan S. Rawat, Ramandeep Singh

**Affiliations:** ^1^Tuberculosis Research Laboratory, Translational Health Science and Technology Institute, Faridabad, India; ^2^Department of Chemistry, Faculty of Science, University of Delhi, New Delhi, India; ^3^Laboratory of Mycobacterial Immunology, Department of Life Science, National Institute of Technology, Rourkela, India

**Keywords:** *Mycobacterium tuberculosis*, phenotypic screening, pyrazole scaffold, NSC-18725, autophagy

## Abstract

The increasing incident rates of drug-resistant tuberculosis (DR-TB) is a global health concern and has been further complicated by the emergence of extensive and total drug-resistant strains. Identification of new chemical entities which are compatible with first-line TB drugs, possess activity against DR-, and metabolically less active bacteria is required to tackle this epidemic. Here, we have performed phenotypic screening of a small molecule library against *Mycobacterium bovis* BCG and identified 24 scaffolds that exhibited MIC_99_ values of at least 2.5 μM. The most potent small molecule identified in our study was a nitroso containing pyrazole derivative, NSC 18725. We observed a significant reduction in viable bacilli load of starved *Mycobacterium tuberculosis* upon exposure to NSC 18725. The action of NSC 18725 was “synergistic” with isoniazid (INH) and “additive” with other drugs in our checkerboard assays. Structure-activity relationship (SAR) studies of the parent compound revealed that pyrazole derivatives without a functional group at fourth position lacked anti-mycobacterial activity *in vitro*. The derivative with *para*-chlorophenyl substitution at the first position of the pyrazole ring was the most active scaffold. We also demonstrate that NSC 18725 is able to induce autophagy in differentiated THP-1 macrophages. The induction of autophagy by NSC 18725 is the major mechanism for the killing of intracellular slow and fast-growing mycobacteria. Taken together, these observations support the identification of NSC 18725 as an antimycobacterial compound, which synergizes with INH, is active against non-replicative mycobacteria and induces autophagy in macrophages.

## Introduction

Tuberculosis (TB), is responsible for the highest number of annual deaths among the infectious diseases (Glaziou et al., 2018). Furthermore, approximately 1.7 billion individuals are estimated to be latently infected with *Mycobacterium tuberculosis*. These individuals are asymptomatic, non-infectious but at a risk of developing disease during their lifetime (Glaziou et al., 2018). The current regimen for TB treatment comprises of an intensive phase of 2 months of administration of isoniazid (INH), rifampicin (RIF), ethambutol (EMB), and pyrazinamide (PZA) followed by a 4-month continuation phase for INH and RIF administration (Snider and Roper, 1992; Bass et al., 1994). Several factors, such as poor patient compliance, low tolerability, and sub-optimal drug concentration contribute to the emergence of drug resistant (DR-) strains. Approximately, 3.5% of newly diagnosed and 18% of previously treated TB cases are estimated to be multi-drug resistant TB (MDR-TB), which are defined as having resistance to both INH and RIF. Among cases of MDR-TB, 8.5% are extensively drug resistant TB (XDR-TB), defined as individuals having resistance to at least one fluoroquinolone and a second-line injectable drug in addition to INH and RIF (Horsburgh et al., 2015). The cure rates in individuals with drug-susceptible TB (DS-TB), MDR-TB and XDR-TB, are 82, 55, and 34%, respectively (Glaziou et al., 2018). Therefore, it is imperative to design better tolerated and shorter drug regimens to eliminate both DS- and DR-TB. The new candidate drug should (i) target a novel metabolic pathway, (ii) possess activity against DR-strains and metabolically dormant bacteria, and (iii) be compatible with current first-line TB and anti-retroviral therapy.

High-throughput phenotypic screening is the most successful approach for identification of new chemical entities against *M. tuberculosis*. Phenotypic screening addresses challenges associated with cell wall penetration, pro-drug activation and results in the identification of accessible and essential bacterial targets (Swinney, 2013; Dhiman and Singh, 2018; Yuan and Sampson, 2018). Several groups have performed modified phenotypic screening by incorporating conditions such as acidic, low oxygen, nutrient starvation, reactive nitrogen intermediates, and fatty acids as carbon source in their screening assays (Cho et al., 2007; Mak et al., 2012; Grant et al., 2013; VanderVen et al., 2015; Early et al., 2019). In addition, high-content screening has also resulted in identification of compounds that inhibit growth of intracellular *M. tuberculosis* (Christophe et al., 2009, 2010; Brodin et al., 2010; Pethe et al., 2013; Stanley et al., 2014). Target-based phenotypic screening combines the advantage of both phenotypic and target-based screening for validation of various metabolic pathways as drug-targets and identification of small molecules targeting these essential enzymes (Bogatcheva et al., 2010; Wilson et al., 2013; Moreira et al., 2015). The combination of phenotypic screening and whole-genome sequencing of the DR-strains has led to identification of various scaffolds that are currently being evaluated in different stages of clinical trials (Dhiman and Singh, 2018; Yuan and Sampson, 2018). Among these, Bedaquiline (BDQ, targeting ATP synthase), Pretomanid (PA-824), and Delamanid (OPC-68683, targeting bacterial respiration) have been recently FDA-approved for administration in individuals with MDR-TB (Diacon et al., 2014; Li H. et al., 2019; Li Y. et al., 2019).

In the present study, we have performed conventional phenotypic screening to identify small molecules that possess anti-tubercular activity. Among the identified anti-mycobacterial compounds, NSC 18725 was the most potent scaffold that displayed an MIC_99_ value of 0.3125 μM against both fast and slow growing mycobacteria in liquid cultures. The lead compound possessed activity against starved *M. tuberculosis* and was synergistic with first-line TB drug, INH *in vitro*. Using medicinal chemistry approach, we demonstrate that the nitroso functional group is important for NSC 18725 activity. Further, we show that NSC 18725 induces autophagy and inhibits survival of intracellular *M. tuberculosis* in human macrophages. Taken together, we have identified an anti-tubercular lead compound for future mechanistic and structure-based drug design studies.

## Materials and Methods

### Cell Culture and Reagents

The maintenance and differentiation of THP-1, a human monocytic cell line, into macrophages (THP-1) was performed as previously described (Mawatwal et al., 2017). The details of cell culture reagents used in the present study are provided in Supplementary Text 1.

### Bacterial Strains and Growth Conditions

The culturing of various mycobacterial strains was carried out in Middlebrook (MB) 7H9 medium supplemented with 0.2% glycerol, 1 × Albumin-Dextrose-Saline (ADS), 0.05% Tween-80, or 7H11 agar supplemented with 1 × Oleic acid-Albumin-Dextrose-Saline (OADS) as previously described (Singh et al., 2013). For MIC_99_ determination assays, *Staphylococcus aureus* (ATCC-BAA-976), *Klebsiella pneumoniae* (ATCC-33495), and *Pseudomonas aeruginosa* (ATCC-2785) were cultured in Mueller-Hinton broth. *Enterococcus faecium* (ATCC-19434), *Acinetobacter baumannii* (ATCC-BAA-2800), and *Escherichia coli* MSG1655 were cultured in brain heart infusion broth, tryptic soy broth, and Luria-Bertani broth, respectively.

### Phenotypic Screening and MIC_99_ Determination Assays

*In vitro* MIC_99_ determination assays against various bacterial strains were determined as reported previously (Kidwai et al., 2017). Preliminary screening of small molecular library at 10 μM concentration was performed using *Mycobacterium bovis* BCG as a host strain. For actual MIC_99_ determination, the plates were incubated at 37°C for 1 day in the case of ESKAPE pathogens, 2 days in the case of *Mycobacterium smegmatis* and 10–14 days in the case of *M. bovis* BCG and *M. tuberculosis*. The lowest concentration of drug at which no visible growth was observed is reported as the MIC_99_ values. All assay plates included no drug, medium only controls, and positive controls such as INH for *M. tuberculosis* and *M. bovis* BCG and ampicillin or tetracycline for ESKAPE pathogens. We also determined the synergy of the lead compound NSC 18725 with various first-line TB drugs, INH, RIF, or EMB and drugs in clinical trials, BTZ043 or BDQ or PA-824 using checkerboard assay. The fractional inhibitory concentration index (ΣFIC) in various drug-combinations was calculated as previously described (Odds, 2003). For *in vitro* killing experiments, early logarithmic cultures (OD_600 nm_ ∼0.2) and nutritionally starved cultures were exposed to various drugs at 10 × MIC_99_ concentration as described previously (Betts et al., 2002; Kidwai et al., 2017). For nutritionally starved bacteria, mid-log phase cultures were washed with 1 × PBS, resuspended in 1 × PBS and exposed to 10 × MIC_99_ of drugs. After 7 days of exposure, 10-fold serial dilutions were prepared and plated on MB7H11 plates at 37°C for 3–4 weeks.

### Cell Viability and Intracellular Killing Experiments

Cell viability of THP-1 cells after exposure to drugs was determined using Cell Proliferation Reagent, WST-1 as per manufacturer’s recommendation (Sigma-Aldrich, St. Louis, MO, United States). For macrophage killing experiments, THP-1 cells were infected with single-cell bacterial suspensions as previously described (Mawatwal et al., 2017). After 4 h post-infection, the extracellular bacteria were removed by overlaying macrophages with RPMI medium containing 200 μg/ml of amikacin. After 2 h of incubation, cells were washed and infected macrophages were overlaid with RPMI medium containing drugs for indicated time points. In another experiment, infected macrophages were pre-treated for 1 h with 3-methyl adenine (3-MA, 10 mM), a selective PI3K inhibitor that inhibits autophagy before treating with NSC 18725 for varied time points. Co-localization experiments were performed by infecting THP-1 cells with GFP labeled *M. bovis* BCG at a MOI of 1:10 as described above followed by treatment with NSC 18725 treatment for 12 h. For bacterial enumeration, 10-fold serial dilutions were prepared and plated on MB7H11 plates at 37°C for 3–4 weeks.

### Confocal Microscopy Experiments

The formation and counting of LC3 puncta were estimated using a previously published protocol (Mawatwal et al., 2017). Briefly, drug-treated macrophages were fixed, permeabilized, and stained with specific antibodies. The formation of LC3 puncta was manually counted in approximately 50 cells for each experiment. In a separate experiment, vacuolar ATPase inhibitor, Bafilomycin A1 (Baf-A1, 50 nM) was added 3 h prior to completion of NSC 18725 treatment followed by estimation of LC3 puncta. Further, monodansylcadaverine (MDC) staining was also performed in drug treated THP-1 macrophages as previously described (Mawatwal et al., 2018). The images were acquired using confocal scanning laser microscope (CSLM, Leica Microsystems, Wetzlar, Germany) and were finally processed for presentation using Adobe Photoshop software. In co-localization experiments, macrophages were fixed, stained for LC3 and visualized under confocal microscope using same methodology as discussed above. The% co-localization between GFP labeled *M. bovis* BCG and LC3 was calculated by counting more than 50 bacteria in at least five or six random fields.

### Western Blot Analysis

The expression analysis of various autophagy markers such as Beclin-1 and Atg 3 in THP-1 macrophages was quantified by Western blot analysis as per manufacturer’s recommendations. Briefly, the protein samples were prepared in radioimmunoprecipitation assay (RIPA) buffer containing protease inhibitors. The samples were fractionated through SDS-PAGE, transferred to nitrocellulose membrane, probed with appropriate antibodies, and detected using ECL kit. The relative fold intensities in drug treated samples in comparison to control samples were quantified using ImageJ software (NIH, United States).

### Chemical Synthesis of Various Pyrazole Derivatives

The reagents for chemical synthesis of pyrazole derivatives were purchased from Spectrochem, India. The formation of the final products was monitored by thin-layer chromatography (TLC). The purification of the final products was performed by column chromatography using silica gel. The melting points of various compounds were recorded on EZ-Melt automated melting point apparatus, Stanford Research Systems and are uncorrected. IR-spectra were recorded on Perkin-Elmer FT-IR spectrophotometer using KBr pellets, and the values are expressed in cm^–1^. ^1^H NMR (400 MHz) and ^13^C NMR (100 MHz) spectra were recorded on Jeol ECX spectrospin instrument using CDCl_3_ as a solvent with TMS as an internal reference. The chemical shift values were expressed on δ scale and the coupling constant (*J*) in Hz. The mass data were recorded in Jeol-Accu TOF JMS-T100LC and micromass LCT mass spectrometer/Data system. The synthesis and characterization details of various small molecules are described in Supplementary Text 1.

### Statistical Analysis

Differences between groups were determined by paired (two-tailed) *t* test. Differences were considered significant at a *P* value of <0.05. GraphPad Prism version 8 (GraphPad Software Inc., San Jose, CA, United States) was used for statistical analysis and the generation of graphs.

## Results

### Identification of NSC 18725 as a Highly Potent and Specific Hit for *Mycobacterium tuberculosis*

**FIGURE 1:**
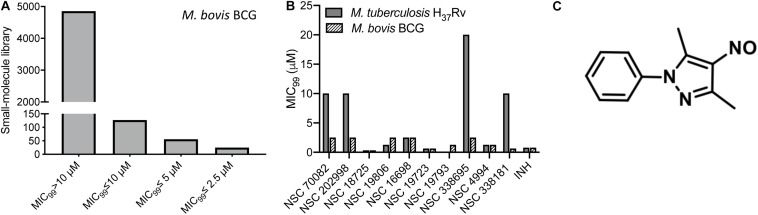
Phenotypic screening of small molecule library obtained from NCI-DTP. **(A)** The small molecule library was evaluated for antimycobacterial activity at a single concentration of 10 μM. Bars are showing number of hits that possessed activity >10 μM, between 5 and 10 μM, between 5 and 2.5 μM, and less than 2.5 μM. **(B)** MIC_99_ values for the few selected small molecules were determined as mentioned in section “Materials and Methods.” The value on *y*-axis denotes MIC_99_ values and *x*-axis denotes Ids of compounds tested for antimycobacterial activity. **(C)** Chemical structure of most potent hit (NSC 18725; 3,5-dimethyl-4-nitroso-1-phenylpyrazole) identified in our phenotypic screening.

In order to identify novel scaffolds with anti-tubercular activity, we screened approximately 5,000 small molecules using *M. bovis* BCG as a host strain. The small molecule library was procured from the National Institutes of Health and compounds belonged to either Open Set II or Oncology Set V. Initially, the preliminary screening was performed at a single concentration of 10 μM, and we observed a hit rate of 4.14% with 207 compounds inhibiting bacterial growth by more than 99% (Figure 1A). These active scaffolds were re-evaluated for MIC_99_ determination in a dose dependent manner. Among the active scaffolds, 127, 56, and 24 compounds displayed MIC_99_ value in the range of 5–10 μM, 2.5– 5 μM, and less than 2.5 μM, respectively (Figure 1A). Among the scaffolds that displayed MIC_99_ below 2.5 μM, we selected 10 preliminary hits, and these were evaluated for their anti-tubercular activity (Table 1 and Supplementary Figure S1). As shown in Figure 1B, we observed that MIC_99_ values of NSC 18725, NSC 19806, NSC 16698, NSC 19723, NSC 19793, and NSC 4994 were comparable against both *M. bovis* BCG and *M. tuberculosis*. However, NSC 70082, NSC 202998, NSC 338695, and NSC 338181 showed less potency against *in vitro* grown cultures of *M. tuberculosis* in comparison to their activity against *M. bovis* BCG (Figure 1B). The most potent hits identified in our phenotypic screening were NSC 18725 and NSC 19723, and their activity was comparable to the activity observed for INH, a front-line TB drug (Table 1).

**TABLE 1:** List of compounds displaying MIC_99_ values less than 2.5 μM identified from phenotypic screening performed in the present study.

**S. No**	**NSC number**	**Compound name**	**Molecular weight (Daltons)**	**MIC_99_ (*M. bovis* BCG)**
1	NSC 19893	5-Fluorouracil	130.08	0.156
2	NSC 15558	(4-Fluorophenyl)(oxo)arsane	186.01	1.25
3	NSC 70082	Diethylcarbamodithioic acid; tellurium	276.9	2.5
4	NSC 203105	Mercury, bis(1-butanethiolato)-	290.78	2.5
5	NSC 202998	Phenazine 5-oxide	196.2	2.5
6	NSC 18725	3,5-Dimethyl-4-nitroso-1-phenylpyrazole	201.22	0.3125
7	NSC 19806	Cinnamaldehyde, alpha-bromo-	211.05	2.5
8	NSC 16698	2-Methoxy-4-[(Z)-2-methyl-3-nitroprop-1-enyl]phenol	223.22	2.5
9	NSC 12470	Ethyl 2-acetamido-2-cyano-5-oxopentanoate	226.23	0.156
10	NSC 338695	Benzo[g]isoquinoline-5,10-dione	209.2	2.5
11	NSC 4830	Pyridylmercuric acetate	337.73	1.25
12	NSC 4773	Phenylmercuric hydroxide	295.71	0.156
13	NSC 4994	1-Chloro-5-nitroanthraquinone	287.65	1.25
14	NSC 338181	5-[(4-Chlorophenyl)hydrazinylidene]-2-(dimethylamino)-6-methylpyrimidin-4-one	291.73	0.625
15	NSC 269612	7-Chloro-[1,4]dithiino[2,3-b]quinoxaline-2,3-dicarbonitrile	302.8	1.25
16	NSC 19723	[(E)-(4-Prop-2-enoxyphenyl)methylideneamino]thiourea	235.31	0.625
17	NSC 19793	(1Z)-1-(4-Chlorophenyl)-2-diazonio-3-methoxy-3-oxoprop-1-en-1-olate	238.63	1.25
18	NSC 338107	1-(2H-Tetrazol-5-ylhydrazinylidene)naphthalen-2-one	240.22	1.25
19	NSC 4603	Chloro(2,2-dimethylpropyl)mercury	307.18	0.156
20	NSC 63142	N-[(E)-1-(3-Bromophenyl)ethylideneamino]pyridine-4-carboxamide	318.17	1.25
21	NSC 4772	Nitrooxy(phenyl)mercury or Phermernite	339.7	0.156
22	NSC 60777	3-Methoxyestra-1,3,5(10)-triene-16,17-dione 16-oxime	313.4	2.5
23	NSC 36758	Tolonium chloride, (7-amino-8-methylphenothiazin-3-ylidene)-dimethylazanium;chloride	305.8	1.56
24	NSC 171303	3-Nitro-N-(5-nitro-1,3-thiazol-2-yl) benzamide	294.25	2.5

**FIGURE 2:**
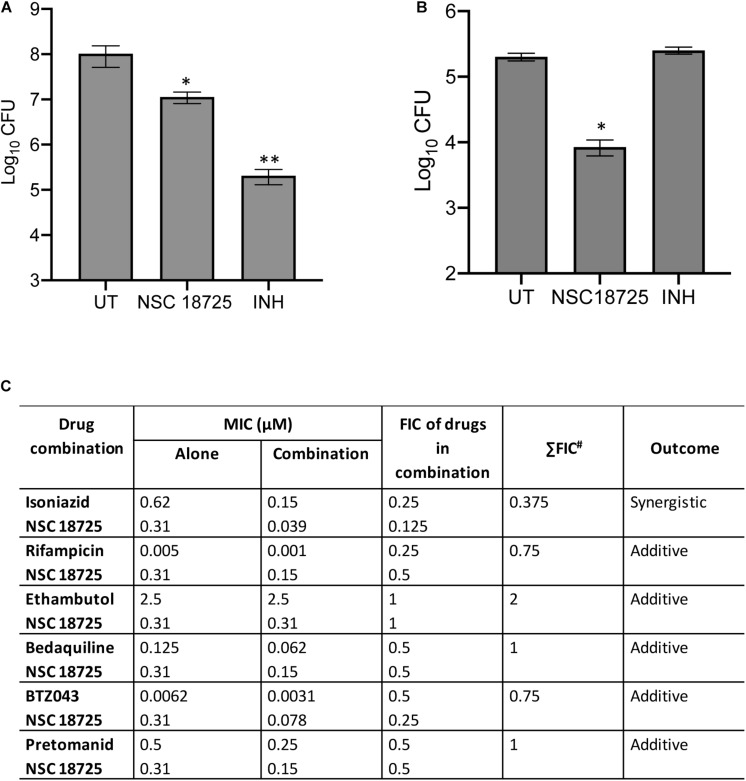
**(A,B)** Time kill kinetics of NSC 18725 against *Mycobacterium bovis* BCG and *Mycobacterium tuberculosis*. **(A)** *M. bovis* BCG was grown till early logarithmic phase (OD_600 nm_ ∼0.2) and subsequently exposed to either NSC 18725 or INH for 7 days. **(B)** The starved *M. tuberculosis* cultures were exposed to either NSC 18725 or INH for 7 days. For bacterial enumeration, 10.0-fold serial dilutions were prepared and 100 μl was plated on MB7H11 plates at 37°C for 3–4 weeks. The data shown in panels **(A,B)** are mean ±SE of CFU obtained from three independent experiments. *P* < 0.05 and *P* < 0.01 are represented as ^∗^ and ^∗∗^, respectively. **(C)** Synergy experiments of NSC 18725 with first-line TB drugs and drugs in clinical trials against *M. tuberculosis* using checkerboard assay. Two-fold serial dilutions of NSC 18725 prepared horizontally were cross-diluted vertically with two-fold serial dilutions of other drugs and ΣFIC values were calculated for each combination. Combinations with best ΣFIC values are shown.

In the subsequent sections, we would discuss results of structure-activity relationship (SAR) and activity of NSC 18725 against mycobacteria *in vitro* and in macrophages (Figure 1C). We next determined the antimicrobial spectrum of NSC 18725 by evaluating its activity against well-characterized ESKAPE pathogens. As shown in Table 2, we noticed that NSC 18725 was inactive against *E. coli* and ESKAPE pathogens *in vitro* even at 25 μM. As shown in Table 2, the control drugs inhibited the growth of ESKAPE pathogens *in vitro* in the expected range. We also evaluated NSC 18725 for activity against fast-growing mycobacterial species *M. smeg*matis and observed that the MIC_99_ value was similar to that obtained against slow growing mycobacteria (Table 2). Taken together, these results demonstrate that NSC 18725 inhibits a metabolic pathway that is vital for *in vitro* growth of mycobacteria. We next determined the mode of mycobacterial killing by NSC 18725 *in vitro*. As shown in Figure 2A, we observed that exposure of *M. bovis* BCG early logarithmic cultures to NSC 18725 resulted in reduction of bacterial counts by ∼9.0 folds in comparison to untreated samples (^∗^*P* < 0.05). As expected, exposure of early logarithmic cultures to INH for 7 days resulted in ∼450-fold reduction in bacterial counts (Figure 2A, ^∗∗^*P* < 0.01). Several studies have shown that *M. tuberculosis* enters into dormancy in host tissues by slowing down its metabolism, and this metabolically less active dormant bacteria is tolerant to front-line TB drugs (Wayne and Sohaskey, 2001; Peddireddy et al., 2017). Next, the activity of NSC 18725 was evaluated against non-replicating persistent *M. tuberculosis* using nutrient-starvation model (Betts et al., 2002). Interestingly, we observed that exposure to NSC 18725 results in the killing of starved bacteria in a bactericidal manner. As shown in Figure 2B, the bacterial counts declined by ∼24.0-fold upon exposure to NSC 18725 (^∗^*P* < 0.05). As expected, nutrient deprived-cultures of *M. tuberculosis* were resistant to killing by INH after 7 days of exposure (Figure 2B). These observations indicate that NSC 18725 targets a metabolic pathway that is essential for *M. tuberculosis* to survive in nutrient limiting growth conditions.

**TABLE 2:** Activity of NSC 18725 against *Mycobacterium smegmatis* and ESKAPE Pathogens.

**Strain name**	**NSC 18725 (μM)**	**Tetracycline (μg/ml)**	**Ampicillin (μg/ml)**	**Rifampicin (μM)**
*E. coli* MG1655	50	0.38	Not done	Not done
*S. aureus* (ATCC-BAA-976)	50	<0.09	Not done	Not done
*K. pneumoniae* (ATCC – 33495)	25	25	Not done	Not done
*P. aeruginosa* (ATCC-2785)	50	12.5	Not done	Not done
*E. faecium* (ATCC-19434)	>100	0.39	3.125	10
*A. baumannii* (ATCC-BAA-2800)	25	>50	>200	10
*M. smegmatis* mc^2^155	0.39–0.78	Not done	Not done	Not done

### NSC 18725 Potentiates the Anti-tubercular Efficacy of Front-Line Anti-tubercular Drugs and Drugs in Clinical Trials

In order to tackle the threat imposed by anti-microbial resistance, there is an urgent need to identify small molecules that are compatible with first-line TB drugs and possess activity against DR-TB. Hence, we investigated the interactions between NSC 18725 and other front-line TB drugs or drugs in clinical trials. We measured the activity of NSC 18725 either alone or in combination with either INH or RIF or EMB or BDQ or BTZ043 or PA-824 using checkerboard assay. As shown in Figure 2C, NSC 18725 synergizes with INH against *M. tuberculosis* with a ΣFIC value of 0.375 in our checkerboard experiments. This combination improved the individual MIC_99_ values of NSC 18725 and INH by 8.0 fold and 4.0 fold, respectively. The ΣFIC of NSC 18725 with RIF, EMB, BDQ, BTZ043, and PA-824 was approximately 0.75, 2, 1, 0.75, and 1, respectively suggesting the additive effect in these drug-combinations (Figure 2C). Taken together, these data augur well for future evaluation of NSC18725 in combination with first-line TB drugs in particular INH against *M. tuberculosis*.

### Structure-Activity Relationship Studies of NSC 18725

**FIGURE 3:**
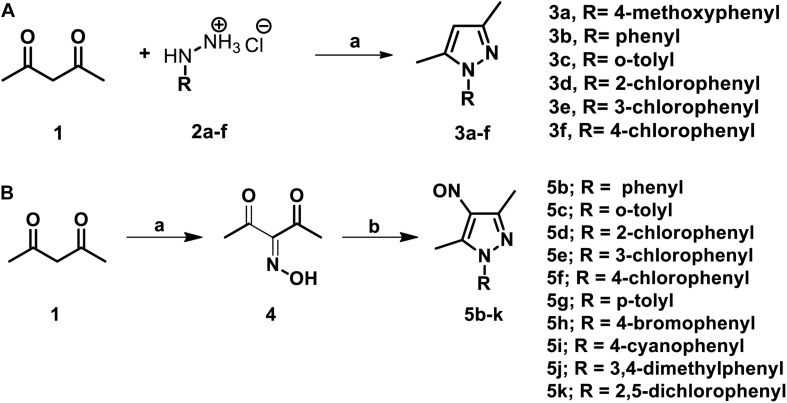
**(A)** The schematic for synthesis of pyrazole derivatives. The commercially available acetylacetone was condensed with substituted phenyl hydrazine hydrochloride (2a–f) at 90°C in a solvent system of glycerol-water (1:1). The reaction was allowed to proceed for 3–4 h and the desired pyrazole derivatives were purified using column chromatography. **(B)** The schematic for synthesis of pyrazole derivatives with nitroso functional group at the fourth position. The synthesis of nitroso containing derivatives was initiated by reacting commercially available acetylacetone with NaNO_2_ and diluted HCl at 0°C for 20 min resulting in the formation of intermediate 3-(hydroxyimino) pentane-2,4-dione. The intermediate (4) was subsequently subjected to condensation with various substituted phenyl hydrazone hydrochlorides and final products were purified using column chromatography.

**FIGURE 4:**
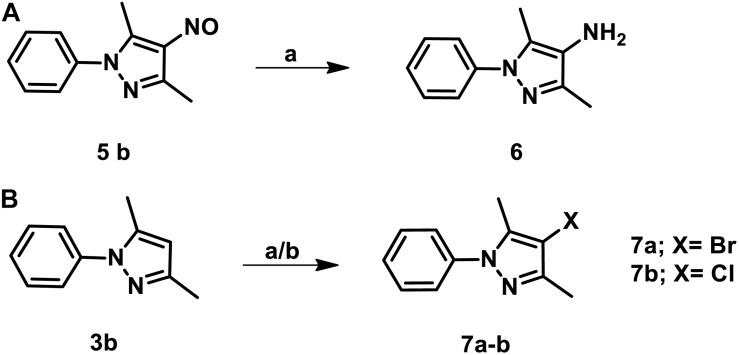
**(A)** The schematic for the synthesis of 3,5-dimethyl-1-phenyl-1H-pyrazol-4-amine. The nitroso group of the parent compound was reduced by catalytic hydrogenation and the desired product was obtained at a yield of 85% and purified using column chromatography. **(B)** The procedure for the synthesis of 4-bromo/chloro pyrazole derivatives. The halogen groups such as Cl, Br were introduced by reacting 3,5-dimethyl-1-phenyl-1*H*-pyrazole with either *N*-bromosuccinimide or *N*-chlorosuccinimide. The desired products were obtained with a yield of 75% and purified using column chromatography.

The parent compound, NSC 18725 (compound 5b, 3,5-dimethyl-4- nitroso-1-phenyl-1*H*-pyrazole), was chemically synthesized and evaluated for its activity against slow growing mycobacteria in liquid cultures. The synthesized parent compound (5b) displayed a MIC_99_ value of 0.3125 μM, and this was similar to the activity obtained from our phenotypic screening (Table 3). In order to design a more potent analog, we synthesized series of NSC 18725 structural analogs using medicinal chemistry approach and evaluated their *in vitro* anti-mycobacterial activity. We synthesized two series of compounds. In Series I the substituted phenyl ring was attached to the N-1 position of the pyrazole ring and lacked any substitution at the fourth position of the pyrazole ring (3a–3f, Figure 3A). In Series II, the nitroso group was introduced at the fourth position of the pyrazole ring and the substituted phenyl ring was varied at the N-1 position of the pyrazole ring (5b–5k, Figure 3B). Subsequently, the nitroso group of the parent compound (5b) was reduced by catalytic hydrogenation using H_2_ gas in the presence of a catalyst, Pd/C (6a, Figure 4A). Finally, the halogen groups were introduced at the fourth position of the pyrazole ring by reacting 3,5-dimethyl-1-phenyl-1*H*-pyrazole (3b) with either *N*-bromosuccinimide or *N*-chlorosuccinimide (7a, 7b, Figure 4B). The details of the synthesis and characterization of various scaffolds are provided in Supplementary Text 1.

**TABLE 3:** *In vitro* MIC_99_ determination of NSC 18725 and its derivatives against both *Mycobacterium tuberculosis* H37Rv and *Mycobacterium bovis* BCG.

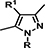					
**S. No.**	**Compound code**	**R**	**R^1^**	**MIC_99_ (μM) (*M. tuberculosis* H_37_Rv)**	**MIC_99_ (*M. bovis* BCG)**

1	3a	4-methoxyphenyl	H	>50	>50
2	3b	Phenyl	H	>50	>50
3	3c	o-tolyl	H	>50	>50
4	3d	2-chlorophenyl	H	>50	>50
5	3e	3-chlorophenyl	H	>50	>50
6	3f	4-chlorophenyl	H	>50	>50
7	5b	Phenyl	NO	0.3125	0.3125
8	5c	o-tolyl	NO	0.156	0.078–0.156
9	5d	2-chlorophenyl	NO	0.3125–0.625	0.3125
10	5e	3-chlorophenyl	NO	0.3125	0.3125–0.625
11	5f	4-chlorophenyl	NO	0.039–0.078	0.039
12	5g	p-tolyl	NO	0.078	0.078–0.156
13	5h	4-bromophenyl	NO	0.3125	0.3125
14	5i	4-cyanophenyl	NO	0.156	0.156
15	5j	3,4dimethylphenyl	NO	0.3125	0.3125
16	5k	2,5-dichlorophenyl	NO	0.3125–0.625	0.3125
17	6	Phenyl	NH_2_	>50	>50
18	7a	Phenyl	Br	>50	>50
19	7b	Phenyl	Cl	>50	>50

In our MIC_99_ determination assays, we observed that pyrazole derivatives (3a–3f) lacking a functional group at the fourth position were inactive against *M. tuberculosis* and displayed an MIC_99_ value greater than 50 μM (Table 3). We also noticed that derivatives (5b–5k) having the nitroso functional group at the fourth position were active and displayed MIC_99_ value in the range of 0.039–6.25 μM. Among these molecules, pyrazole derivative with *para*-chlorophenyl at the first position displayed the highest activity in the range of 0.039–0.078 μM against *M. tuberculosis* (5f, Table 3). The pyrazole derivative with *p*-tolyl substitution also displayed 4.0-fold higher activity in comparison to the parent compound (5g, Table 3). We also observed that pyrazole derivative with nitrile substitution at *para-*position of the phenyl ring (5i) enhanced the activity of the parent compound by 2.0-fold (Table 3). However, a derivative with a bromo-group (5h) substitution at *para-* position of the phenyl group displayed MIC_99_ values that were comparable to those observed for the parent compound. Next, we determined the effect of *ortho*- and *meta*- position substitution of the phenyl ring on NSC 18725 activity. We noticed that changing the position of substitution from *para*- to *ortho*- and *meta*- position resulted in a decrease of activity by 2.0-fold (5c, with methyl substitution at *ortho*-position), 4.0-fold (5d, with chloro substitution at *ortho*-position) and 4.0-fold (5e, with chloro substitution at *para-* position). Further, multiple substitutions on the phenyl ring resulted in reduced activity (5j; MIC_99_ = 0.3125–0.6250 μM and 5 k; MIC_99_, = 0.3125 μM) in comparison to mono-substituted compounds (Table 3). We observed that the derivatives with multiple substitutions (5j, 5k) on the phenyl ring displayed MIC_99_ values similar to those obtained for the parent compound (Table 3). These observations suggest that nitroso substitution at the fourth position of the pyrazole ring is essential for NSC 18725 activity *in vitro*. Also, substitution at the *para*-position of the phenyl ring with chloro and methyl functional groups improves NSC 18725 anti-tubercular activity.

### NSC 18725 Induces Autophagy in Differentiated THP-1 Macrophages and Inhibits Growth of Intracellular *Mycobacterium tuberculosis*

**FIGURE 5:**
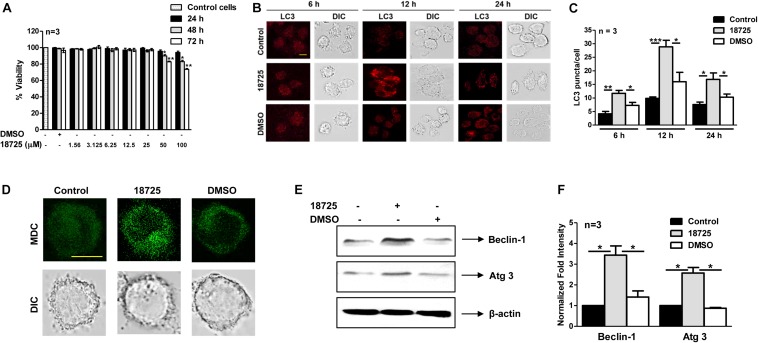
The effect of NSC 18725 pre-treatment on cell viability and autophagy induction in THP-1 cells. **(A)** THP-1 cells were treated with different concentrations of NSC 18725, and cell viability was determined through WST-1 assay. The data shown on *y*-axis is percentage cell viability obtained from control and drug-treated macrophages. **(B)** THP-1 cells were treated with either 25 μM NSC 18725 or DMSO for 6, 12, and 24 h. At designated time points, macrophages were fixed, stained with anti-LC3 antibody, and immunofluorescent images were captured using a confocal microscope. The images shown are representative of three independent experiments. Scale bar given is 10 μM. **(C)** The LC3 puncta formation in images shown in panel **(B)** were counted in a random manner (*n* = 50). The data shown on *y*-axis is mean ±SE of puncta formation/cell obtained from three independent experiments. **(D)** THP-1 cells were pre-treated with either 25 μM NSC 18725 or DMSO for 12 h. Subsequently, MDC staining was performed and images of fixed cells were acquired under a confocal microscope. Images given are the representation of the experiment performed in duplicates. Scale bar given is 10 μM. **(E)** THP-1 cells were pre-incubated with NSC 18725 (25 μM) for 12 h followed by whole cell lysate preparation. The expression of Beclin-1 and Atg 3 in various samples was analyzed through immunoblotting using specific antibodies. The immunoblots shown are representative of three independent experiments. **(F)** This panel depicts the quantification of the fold change in the expression of Beclin-1 and Atg-3 in NSC 18725 pre-treated samples in comparison to control macrophages for the blots shown in panel **(E)**. The data is shown as mean ± SE of fold change in expression obtained from three independent experiments. *P* < 0.001, *P* < 0.01, *P* < 0.05 are represented as ***, **, and *, respectively.

Being a facultative intracellular pathogen, *M. tuberculosis* is able to adapt to various stress conditions encountered in the host and to replicate inside the host macrophage. Macrophages employ numerous antimicrobial mechanisms such as production of reactive oxygen intermediates, reactive nitrogen intermediates, and phagosome lysosome fusion to combat infections. Autophagy is a lysosomal degradative process and can be used by the macrophages to inhibit growth of intracellular *M. tuberculosis* (Lowrie and Andrew, 1988; Bah and Vergne, 2017). Several studies have shown that small molecules inducing autophagy are able to clear intracellular DR- and DS-TB (Kidwai et al., 2017; Mawatwal et al., 2017; Dhiman and Singh, 2018). In order to investigate whether NSC 18725 is able to induce autophagy, we first determined cell viability of THP-1 cells in the presence of different concentrations of drug. We observed that NSC 18725 at 25 μM concentration was non-cytotoxic to THP-1 cells till 72 h of incubation and subsequent experiments were performed at this concentration (Figure 5A). We observed that exposure of THP-1 cells to NSC 18725 at 25 μM concentration resulted in significant LC3 puncta formation after 12 h of incubation, hence this time point was selected for future experiments (Figures 5B,C, ^∗^*P* < 0.05, ^∗∗^*P* < 0.01 and ^∗∗∗^*P* < 0.001). In concordance, MDC staining revealed significant autophagic vacuole formation in NSC 18725 treated THP-1 macrophages, and this observation was further corroborated with specific upregulation of autophagic markers such as Beclin-1 and Atg 3 at protein level in drug-treated samples (Figures 5D,E). As shown in Figure 5F, we observed that Beclin-1 and Atg 3 expression was increased by ∼3.0-fold and 2.5-fold, respectively, in NSC 18725 treated macrophages (Figure 5F, ^∗^*P* < 0.05).

**FIGURE 6:**
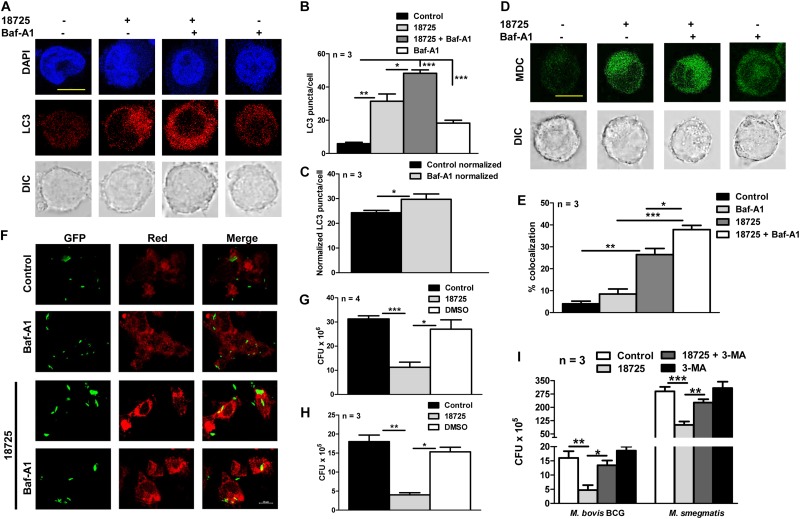
The effect of NSC 18725 on autophagic flux and intracellular mycobacterial growth. **(A)** THP-1 cells were pre-treated with 25 μM NSC 18725 for 12 h and Baf-A1 treatment was performed as described in section “Materials and Methods.” At designated time points, cells were fixed, stained with anti-LC3, and immunofluorescent images were captured using a confocal microscope. The image shown is representative of three independent experiments. Scale bar given is 10 μM. **(B)** The formation of LC3 puncta in panel **(A)** in different samples were quantified in a random manner (*n* = 50). The data shown on *y*-axis is mean ±SE of LC3 puncta formation per cell obtained from three independent experiments. **(C)** Quantitative data depicting normalized values of LC3 puncta formation in 18725 treated THP-1 cells in the absence or presence of Baf-A1. **(D)** MDC staining of macrophages pre-treated with NSC 18725 in the presence or absence of Baf-A1 was performed as described in section “Materials and Methods.” The images shown in this panel are representative of experiments performed in duplicates. Scale bar given is 10 μM. **(E)** THP-1 cells were infected with GFP labeled *M. bovis* BCG for 4 h at a MOI of 1:10 before treating with NSC 18725 for 12 h. In few combinations, Baf-A1 was added as described earlier before staining with LC3 antibody followed by visualization under confocal microscope. This panel represents the cumulative quantification depicting co-localization between phagosomes and LC3 in three independent experiments and data is represented as mean ± SE **(F)** The images shown in this panel are representative of experiments performed in triplicates. Scale bar given is 10 μM. **(G,H)** THP-1 macrophages were infected with either *Mycobacterium smegmatis* with MOI of 1:1 **(G)** or *Mycobacterium bovis* BCG with MOI 1:10 **(H)** and anti-tubercular activity of NSC 18725 against intracellular mycobacteria was determined as described in section “Materials and Methods.” **(I)** The antimycobacterial activity of NSC 18725 against intracellular *M. smegmatis* and *M. bovis BCG* was determined in the presence of 3-MA as described in section “Materials and Methods.” The data shown in this panel is mean ± SE of bacterial numbers obtained from three or four independent experiments. *P* < 0.001, *P* < 0.01, *P* < 0.05 are represented as ***, **, and *, respectively.

Previous studies have shown that there is an accumulation of LC3 puncta or autophagic vacuole formation during autophagy inhibition, therefore, we next performed autophagy experiments in NSC 18725 treated THP-1 cells in the presence of Baf-A1 (Yoshii and Mizushima, 2017). In concordance with our earlier results, we observed that Baf-A1 addition significantly enhanced LC3 puncta and autophagic vacuole formation in NSC 18725 pre-treated THP-1 cells in comparison to untreated macrophages (Figures 6A–D, ^∗^*P* < 0.05, ^∗∗^*P* < 0.01, and ^∗∗∗^*P* < 0.001). These observations were further validated by quantifying co-localization between GFP labeled *M. bovis* BCG and LC3 in NSC 18725 treated THP-1 cells in the absence or presence of Baf-A1. As shown in Figures 6E,F, significant co-localization was observed in treated THP-1 cells in the presence of Baf-A1 in comparison to only NSC 18725 treated cells (37.9 ± 1.9% vs. 26.4 ± 2.8%, ^∗^*P* < 0.05). These observations indicate that NSC 18725 induces autophagy in human macrophages. Several reports have shown that modulation of autophagy by small molecules results in faster clearance of intracellular *M. tuberculosis*, therefore, we further evaluated the antimicrobial efficacy of NSC 18725 against the pathogen replicating inside macrophages (Kidwai et al., 2017; Mawatwal et al., 2017; Dhiman and Singh, 2018). In concordance with previous studies, we observed that autophagy induction upon NSC 18725 treatment inhibited the growth of mycobacteria in human macrophages. We observed that exposure to NSC 18725 resulted in approximately 64 and 78% significant reduction in bacterial counts of *M. smegmatis* and *M. bovis* BCG, respectively in comparison to untreated and DMSO treated macrophages (Figures 6G,H, ^∗^*P* < 0.05, ^∗∗^*P* < 0.01, ^∗∗∗^*P* < 0.001). We next studied whether 3-MA inhibited the killing activity of NSC 18725. In concordance with our earlier observations, we demonstrated that preincubation of macrophages with 3-MA reduced the intracellular killing of NSC 18725 (Figure 6I, *^∗^P* < *0.05, ^∗∗^P* < *0.01*). As expected, pre-incubation with 3-MA only has no effect on the intracellular growth of both *M. bovis BCG* and *M. smegmatis*. These findings elucidate that induction of autophagy is the mechanism by which NSC 18725 inhibits the survival of intracellular mycobacteria. Taken together, the observations presented in this study demonstrate that modulation of autophagy by NSC 18725 in human macrophages can be exploited further to design novel therapeutics against TB.

## Discussion

The current scenario of TB epidemiology stresses for the development of new diagnostic tools, vaccines, and drugs to tackle the challenge of DR- and DS-TB. Despite the availability of various scaffolds in clinical pipeline, there is an urgent need to develop new lead molecules that possess activity against DR- and metabolically dormant bacilli. Till date, phenotypic and target-based screening have been extensively utilized for identification and validation of novel anti-tubercular agents. Although, the target-based approach has been the backbone for drug discovery in pharmaceutical industry in past decades, it has failed to show ample success in the area of antitubercular drug discovery. This lack of whole-cell activity for small molecules identified from target-based screening is attributed to their poor penetration. Phenotypic screening has led to identification of various antitubercular scaffolds with a novel mechanism of action (Dhiman and Singh, 2018). The highly infectious and pathogenic nature of *M. tuberculosis* along with the prerequisite for complex infrastructure for handling *M. tuberculosis* led us to use *M. bovis* BCG as a surrogate host for initial screening. In the present study, we have performed whole cell based screening and identified 24 scaffolds that possessed anti-mycobacterial activity below 2.5 μM. In concordance, with previous studies, majority of these compounds showed comparable activity against both *M. bovis* BCG and *M. tuberculosis in vitro* (Taneja and Tyagi, 2007; Altaf et al., 2010; Stanley et al., 2012; Kidwai et al., 2017). However, NSC 70082, NSC 202998, NSC 338695, and NSC 338181 displayed better activity against *M. bovis* BCG in comparison to *M. tuberculosis*. This differential activity could be attributed to (i) altered expression levels of their respective drug-targets in *M. bovis* BCG and *M. tuberculosis* (ii) modification of the drug-target in *M. tuberculosis* or (iii) differential ability of the small molecules to penetrate in *M. bovis* BCG and *M. tuberculosis*.

In the present study, we have performed detailed characterization of NSC 18725 (3,5-dimethyl-4-nitroso-1-phenylpyrazole), the most active compound identified in our phenotypic screening. Pyrazoles containing pharmaco-active agents are potent medicinal scaffolds and exhibit a broad spectrum of biological activities such as antimicrobial, anti-inflammatory, anti-cancer, analgesic, and neuroprotection (Wilfred et al., 1956, 1958; Slack, 1957; Daidone et al., 1992; Bekhit et al., 2005; Chandra et al., 2010; Ahsan et al., 2011; Keche et al., 2012; Maurya et al., 2013; Alegaon et al., 2014; Pathak et al., 2014; Naim et al., 2016). We also observed that NSC 18725 displayed MIC_99_ value of ∼0.3125 μM against slow growing mycobacteria and was non-cytotoxic to THP-1 macrophages even at 25 μM concentration. SAR studies revealed that the nitroso group is important for anti-tubercular activity associated with this series. In concordance previous studies have also shown that nitro or nitroso functional groups are essential for the anti-tubercular activity of small molecules (Singh et al., 2008; Kidwai et al., 2017, 2019). We also show that substitution at the *para*-position of the phenyl ring with either electron withdrawing group such as (chloro and cyano) or electron donating groups (such as methyl) improved NSC 18725 activity *in vitro*. A major limitation in the field of drug development is target identification of small molecules identified from phenotypic screens. In the present work, we have also tried to generate resistant mutant strains against NSC 18725 but all these attempts have been unsuccessful.

Indiscriminate use of antimicrobial drugs globally has resulted in increased incident rates of various DR-TB strains. Several studies have shown that pyrazole derivatives possess activities against various microbial species such as *S. aureus*, *P. aeruginosa*, *Bacillus subtilis*, *E. Coli*, and *Salmonella typhi* as well as fungal strains such as *Aspergillus niger* and *Candida albicans* (Keche et al., 2012; Naim et al., 2016; Karrouchi et al., 2018). Therefore, we also evaluated the ability of NSC 18725 against a panel of resistant strains that constitute ESKAPE pathogens. However, we observed that NSC 18725 failed to inhibit the *in vitro* growth of the tested ESKAPE pathogens thereby indicating that these pyrazole derivatives lack cross resistance with existing drugs and target a mycobacteria specific metabolic pathway. Another challenge in the field of TB chemotherapy is that among various clinical candidates very few scaffolds are able to inhibit the growth of dormant bacteria. Here, we show that NSC 18725 is able to kill the dormant population of *M. tuberculosis* thereby indicating that NSC 18725 might target a metabolic pathway which is essential for *M. tuberculosis* to survive in nutrient limiting growth conditions. Most of the compounds that are currently in different stages of clinical trials possess activity against both DS- and DR- strains *in vitro* and show synergistic effect with the current TB drugs. We also observed that NSC 18725 shows synergistic effect with INH and additive effect with other tested TB drugs. Our results demonstrate that if used in combination, NSC 18725 can potentially reduce the dosage associated toxicity associated with TB drugs. These observations suggest that evaluation of NSC 18725 in combination with other first- and second-line drugs could help design better regimens against both DS- and DR-TB infection.

In the present study, we also validated the activity of NSC 18725 against intracellular mycobacteria in macrophage model of infection. We observed that pre-incubation with NSC 18725 resulted in LC3 puncta formation and increased expression of autophagy markers such as Atg 3 and Beclin-1. This NSC18725 mediated modulation of autophagy resulted in inhibition of growth of mycobacteria in infected macrophages. We also observed that pre-incubation of THP-1 macrophages with 3-MA completely abrogated the intracellular activity associated with NSC 18725. Therefore, we hypothesize, that induction of autophagy is the main mechanism by which NSC 18725 inhibits intracellular bacterial growth in macrophages. These observations are in concordance with previous reports showing that induction of autophagy can be harnessed as a host-directed therapy (HDT) either alone or in combination with first-line TB drugs (Dara et al., 2019). Despite identification of autophagy inducers, enough information is not available about the co-operative action of various known or unknown mechanisms regulated by autophagy (Paik et al., 2019). Therefore, evaluation of promising autophagy inducers as host-directed therapy either alone or in combination with first-line TB drugs will refine therapeutic interventions against TB.

Taken together, we have identified a pyrazole derivative that possesses anti-mycobacterial activity. We showed that this compound is active against both actively growing, dormant bacteria, and the nitroso group is essential for the observed anti-tubercular activity. Finally, we also show that NSC 18725 induces autophagy and inhibits the growth of intracellular mycobacteria in macrophages. Further experiments include (i) designing of structural analogs with better therapeutic index, (ii) understanding the mechanism of action of NSC 18725 *in vitro*, (iii) pharmacokinetics and pharmacodynamic studies to determine stability of these series of compounds in serum or plasma of animals, and (iv) evaluating the *in vivo* efficacy of this series in mice model of infection.

## Conclusion

In conclusion, we have identified NSC 18725 as an anti-tubercular compound with the activity comparable to INH, first-line TB drug. In addition, NSC 18725 also possesses activity against dormant *M. tuberculosis in vitro*. We also demonstrate that NSC 18725 augments the host defense mechanisms by inducing autophagy and inhibits *M. tuberculosis* survival in macrophages. Furthermore, NSC 18725 showed synergy with INH and additive effect with other tested drugs in checkerboard assays. We also demonstrated that the nitroso group is essential for the anti-mycobacterial activity of the parent compound. Further, substitution at the *para*-position of the phenyl ring enhanced NSC 18725 activity *in vitro*. Future studies would involve more detailed SAR studies to improve NSC 18725 activity *in vitro* and evaluate the efficacy of this series in aerosol infected mice.

## Data Availability Statement

All datasets generated for this study are included in the article/Supplementary Material.

## Author Contributions

RS conceived the idea and supervised the experiments. The microbiology related experiments were performed by GA, TG, RPS, SK, and PS. Gagandeep and SKK performed chemical synthesis of NSC 18725 analogs. AB performed the autophagy experiments. RD supervised the autophagy experiments. DR supervised the experiments related to chemical synthesis. RS, GA, Gagandeep, and PS wrote the manuscript with inputs from other authors.

## Conflict of Interest

The authors declare that the research was conducted in the absence of any commercial or financial relationships that could be construed as a potential conflict of interest.
