# Supplementary material for: NSC 18725, a Pyrazole Derivative Inhibits Growth of Intracellular Mycobacterium tuberculosis by Induction of Autophagy
Source: Front Microbiol. 2020 Jan 28;10:3051. doi: 10.3389/fmicb.2019.03051 (PMC6999026; doi:10.3389/fmicb.2019.03051)
Supplement: Supplementary file 1 [file Presentation_1.PPTX]

## Slide 1
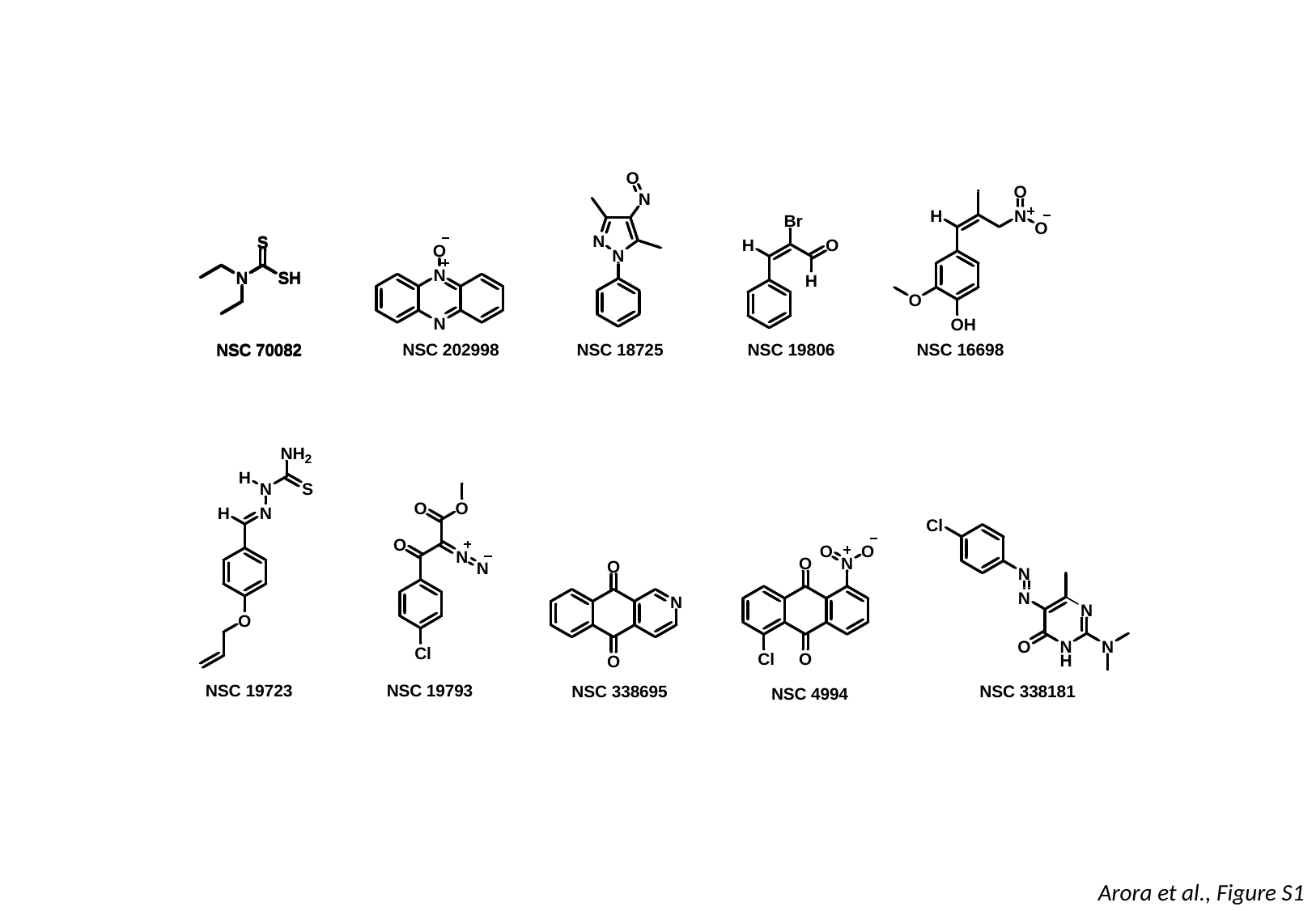

Arora et al., Figure S1

## Slide 2
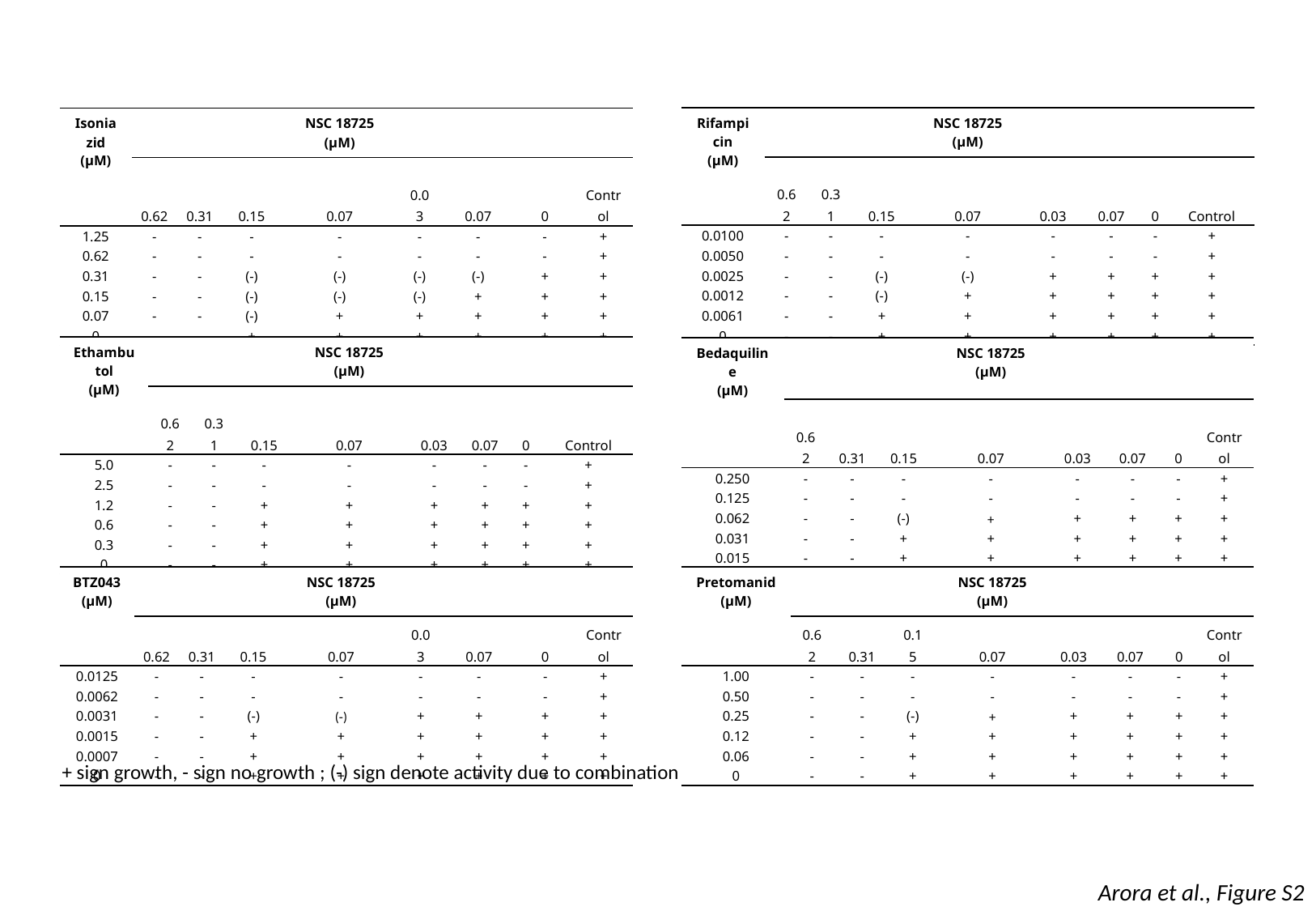

| Rifampicin (µM) | | | | NSC 18725 (µM) | | | | |
| --- | --- | --- | --- | --- | --- | --- | --- | --- |
| | 0.62 | 0.31 | 0.15 | 0.07 | 0.03 | 0.07 | 0 | Control |
| 0.0100 | - | - | - | - | - | - | - | + |
| 0.0050 | - | - | - | - | - | - | - | + |
| 0.0025 | - | - | (-) | (-) | + | + | + | + |
| 0.0012 | - | - | (-) | + | + | + | + | + |
| 0.0061 | - | - | + | + | + | + | + | + |
| 0 | - | - | + | + | + | + | + | + |
| Isoniazid (µM) | | | | NSC 18725 (µM) | | | | |
| --- | --- | --- | --- | --- | --- | --- | --- | --- |
| | 0.62 | 0.31 | 0.15 | 0.07 | 0.03 | 0.07 | 0 | Control |
| 1.25 | - | - | - | - | - | - | - | + |
| 0.62 | - | - | - | - | - | - | - | + |
| 0.31 | - | - | (-) | (-) | (-) | (-) | + | + |
| 0.15 | - | - | (-) | (-) | (-) | + | + | + |
| 0.07 | - | - | (-) | + | + | + | + | + |
| 0 | - | - | + | + | + | + | + | + |
| | | | | | | | | |
| Ethambutol (µM) | | | | NSC 18725 (µM) | | | | |
| --- | --- | --- | --- | --- | --- | --- | --- | --- |
| | 0.62 | 0.31 | 0.15 | 0.07 | 0.03 | 0.07 | 0 | Control |
| 5.0 | - | - | - | - | - | - | - | + |
| 2.5 | - | - | - | - | - | - | - | + |
| 1.2 | - | - | + | + | + | + | + | + |
| 0.6 | - | - | + | + | + | + | + | + |
| 0.3 | - | - | + | + | + | + | + | + |
| 0 | - | - | + | + | + | + | + | + |
| Bedaquiline (µM) | | | | NSC 18725 (µM) | | | | |
| --- | --- | --- | --- | --- | --- | --- | --- | --- |
| | 0.62 | 0.31 | 0.15 | 0.07 | 0.03 | 0.07 | 0 | Control |
| 0.250 | - | - | - | - | - | - | - | + |
| 0.125 | - | - | - | - | - | - | - | + |
| 0.062 | - | - | (-) | + | + | + | + | + |
| 0.031 | - | - | + | + | + | + | + | + |
| 0.015 | - | - | + | + | + | + | + | + |
| 0 | - | - | + | + | + | + | + | + |
| | | | | | | | | |
| BTZ043 (µM) | | | | NSC 18725 (µM) | | | | |
| --- | --- | --- | --- | --- | --- | --- | --- | --- |
| | 0.62 | 0.31 | 0.15 | 0.07 | 0.03 | 0.07 | 0 | Control |
| 0.0125 | - | - | - | - | - | - | - | + |
| 0.0062 | - | - | - | - | - | - | - | + |
| 0.0031 | - | - | (-) | (-) | + | + | + | + |
| 0.0015 | - | - | + | + | + | + | + | + |
| 0.0007 | - | - | + | + | + | + | + | + |
| 0 | - | - | + | + | + | + | + | + |
| | | | | | | | | |
| Pretomanid (µM) | | | | NSC 18725 (µM) | | | | |
| --- | --- | --- | --- | --- | --- | --- | --- | --- |
| | 0.62 | 0.31 | 0.15 | 0.07 | 0.03 | 0.07 | 0 | Control |
| 1.00 | - | - | - | - | - | - | - | + |
| 0.50 | - | - | - | - | - | - | - | + |
| 0.25 | - | - | (-) | + | + | + | + | + |
| 0.12 | - | - | + | + | + | + | + | + |
| 0.06 | - | - | + | + | + | + | + | + |
| 0 | - | - | + | + | + | + | + | + |
| | | | | | | | | |
+ sign growth, - sign no growth ; (-) sign denote activity due to combination
Arora et al., Figure S2
