# Supplementary material for: NSC 18725, a Pyrazole Derivative Inhibits Growth of Intracellular Mycobacterium tuberculosis by Induction of Autophagy
Source: Front Microbiol. 2020 Jan 28;10:3051. doi: 10.3389/fmicb.2019.03051 (PMC6999026; doi:10.3389/fmicb.2019.03051)
Supplement: Supplementary file 2 [file Data_Sheet_1.pdf]

Supplementary Text 1

Materials and Methods

List of cell culture reagents.

Cell culture reagents were procured from Gibco Laboratories (Grand Island, NY, USA). ECL western blotting detection reagent was procured from GE Healthcare (Buckinghamshire, UK). Various primary antibodies used in the study were obtained from Cell Signaling Technology (MA, USA). Phorbol 12-myristate 13-acetate (PMA), monodansylcadaverine (MDC, a fluorescent dye that stains autophagic vacuoles), anti-rabbit HRP antibody, Bafilomycin A1 (Baf-A1, a vacuolar ATPase inhibitor), BCA kit, 100 × protease inhibitor cocktail and Fluoromount aqueous mounting medium were procured from Sigma (St. Louis, MO, USA). Middlebrook growth media 7H9 broth, 7H10 agar, 7H11 agar was ordered from BD (MD, USA). WST-1 cell viability assay kit and other general chemicals unless not mentioned were obtained from HiMedia Laboratory (Mumbai, India).

Chemical synthesis and characterization of various pyrazole derivatives

General procedure for the synthesis of compounds 3a-f.

For synthesis of the pyrazole derivatives, the commercially available acetylacetone (500 mg, 4.99 mmol) and compound 2a-f (4.99 mmol) were mixed in a round bottom flask containing 5 mL glycerol-water (1:1) mixture as a solvent and heated for 3-4 hrs at 90˚C with continuous stirring. The progress of the reaction was monitored by TLC and the reaction mixture was allowed to cool upon completion. The desired final product was extracted multiple times using ethyl acetate. The combined organic layer was washed with water, brine solution, dried by adding anhydrous Na_2SO_4 and the final product was purified by column chromatography.

1-(4-Methoxyphenyl)-3,5-dimethyl-1H-pyrazole (3a): Light yellow oil; Yield: 82%; IR (KBr, cm^{-1}): 2964, 1553, 1514, 1246, 745; ^{1}H NMR (400 MHz, $CDCl_3$): δ 2.23 (s, 3H), 2.28 (s, 3H), 3.83 (s, 3H), 5.95 (s, 1H), 6.94 (d, J = 8.70 Hz, 2H), 7.31 (d, J = 8.70 Hz, 2H); ^{13}C NMR (100 MHz, $CDCl_3$): δ 12.22, 13.58, 55.60, 106.32, 114.17, 126.46, 133.16, 139.56, 148.60, 158.84; ESI-HRMS (m/z) calculated for $C_{12}H_{14}N_2O$: 202.1106; observed: 203.2461 $(MH)^+$.

3,5-dimethyl-1-phenyl-1H-pyrazole (3b): Light yellow oil; Yield: 80%; IR (KBr, cm^{-1}): 2922, 1596, 1500, 1373, 1025, 753; ^{1}H NMR (400 MHz, $CDCl_3$): δ 2.30 (s, 6H), 5.99 (s, 1H), 7.32-7.35 (m, 1H), 7.41-7.46 (m, 4H); ^{13}C NMR (100 MHz, $CDCl_3$): δ 12.46, 13.59, 106.99, 124.84, 127.33, 129.07, 139.48, 139.97, 149.04; ESI-HRMS (m/z) calculated for $C_{11}H_{12}N_2$: 172.1000; observed: 173.1453 $(MH)^+$.

3,5-dimethyl-1-(o-tolyl)-1H-pyrazole (3c): Brown liquid; Yield: 83%; IR (KBr, cm^{-1}): 2923, 1583, 1552, 1287, 1129, 1031, 775; ^{1}H NMR (400 MHz, $CDCl_3$): δ 2.05 (s, 6H), 2.29 (s, 3H), 5.96 (s, 1H), 7.22-7.33 (m, 4H); ^{13}C NMR (100 MHz, $CDCl_3$): δ 11.35, 13.66, 17.30, 105.00, 126.50, 128.00, 129.00,

130.89, 136.39, 138.83, 140.29, 148.57; ESI-HRMS (m/z) calculated for $C_{12}H_{14}N_2$: 186.1157; observed: 187.1643 $(MH)^+$.

1-(2-Chlorophenyl)-3,5-dimethyl-1H-pyrazole (3d): Light yellow oil; Yield: 82%; IR (KBr, cm^{-1}): 2968, 1556, 1496, 1217, 1032, 745, 660; ^1H NMR (400 MHz, $CDCl_3$): δ 2.12 (s, 3H), 2.31 (s, 3H), 6.00 (s, 1H), 7.37-7.41 (m, 3H), 7.50-7.53 (m, 1H); ^{13}C NMR (100 MHz, $CDCl_3$): δ 11.32, 13.69, 105.58, 127.61, 130.02, 130.24, 132.60, 137.56, 141.29, 149.36; ESI-HRMS (m/z) calculated for $C_{11}H_{11}ClN_2$: 206.0611; observed: 207.0686 $(MH)^+$, 209.0645 $(MH+2)^+$.

1-(3-Chlorophenyl)-3,5-dimethyl-1H-pyrazole (3e): Light yellow oil; Yield: 85%; IR (KBr, cm^{-1}): 2973, 1590, 1556, 1489, 1216, 744, 686; ^1H NMR (400 MHz, $CDCl_3$): δ 2.28 (s, 3H), 2.32 (s, 3H), 6.00 (s, 1H), 7.29-7.37 (m, 3H), 7.47-7.48 (m, 1H); ^{13}C NMR (100 MHz, $CDCl_3$): δ 12.59, 13.57, 107.64, 122.56, 124.88, 127.29, 130.02, 134.74, 139.57, 141.03, 149.60; ESI-HRMS (m/z) calculated for $C_{11}H_{11}ClN_2$: 206.0611; observed: 207.0874 $(MH)^+$, 209.0732 $(MH+2)^+$.

1-(4-Chlorophenyl)-3,5-dimethyl-1H-pyrazole (3f): Light yellow oil; Yield: 75%; IR (KBr, cm^{-1}): 2973, 1554, 1498, 1216, 1037, 744, 665; ^1H NMR (400 MHz, $CDCl_3$): δ 2.28-2.29 (m, 6H), 5.99 (s, 1H), 7.35-7.42 (m, 4H); ^{13}C NMR (100 MHz, $CDCl_3$): δ 12.48, 13.56, 107.42, 125.87, 129.22, 132.93, 138.41, 139.50, 149.43; ESI-HRMS (m/z) calculated for $C_{11}H_{11}ClN_2$: 206.0611; observed: 207.0848 $(MH)^+$, 209.0719 $(MH+2)^+$.

General procedure for the synthesis of compounds 5b-k.

Acetylacetone (500 mg, 4.99 mmol) was mixed in a round bottom flask containing diluted HCl (5 mL) and the mixture was cooled in ice bath to 37˚C followed by the addition of $NaNO_2$ (344.95 mg, 5 mmol, 5 mL H_2O) solution. The reaction mixture was subsequently left undisturbed for 20 mins. Simultaneously, substituted phenyl hydrazine hydrochloride (4.99 mmol) was dissolved in a mixture of polyethylene glycol-water (1:1) solvent system. The solution of substituted phenyl hydrazine hydrochloride was added dropwise to the above reaction mixture with continuous stirring and the temperature of the reaction mixture was slowly allowed to rise to 37˚C from room temperature. The obtained green precipitate was filtered, washed with 50% PEG solution, dried and purified by column chromatography using silica gel and ethyl acetate-hexane as a solvent system.

3,5-dimethyl-4-nitroso-1-phenyl-1H-pyrazole (5b): Greenish solid; Yield: 64%; mp 96-98 ºC; IR (KBr, cm^{-1}): 2923, 1543, 1511, 1383, 1088, 1016, 766; ^1H NMR (400 MHz, $CDCl_3$): δ 2.43 (s, 3H), 2.94 (s, 3H), 7.50-7.58 (m, 5H); ^{13}C NMR (100 MHz, $CDCl_3$): δ 11.06, 12.98, 124.99, 129.14, 129.45, 137.61, 160.66; ESI-HRMS (m/z) calculated for $C_{11}H_{11}N_3O$: 201.0902 ; observed: 202.0976 $(MH)^+$.

3,5-dimethyl-4-nitroso-1-(o-tolyl)-1H-pyrazole (5c): Greenish solid; Yield: 66%; mp 68-70 ºC; IR (KBr, cm^{-1}): 2929, 1539, 1512, 1338, 1236, 1018, 771; ^1H NMR (400 MHz, $CDCl_3$): δ 2.14 (s, 3H), 2.45(brs, 3H), 2.66 (brs, 3H), 7.28-7.45 (m, 3H); ^{13}C NMR (100 MHz, $CDCl_3$): δ 10.57, 13.16, 17.44, 127.16, 127.32, 130.42, 131.53, 135.69, 136.44, 160.27; ESI-HRMS (m/z) calculated for $C_{12}H_{13}N_3O$: 215.1059; observed: 216.1125 $(MH)^+$.

1-(2-Chlorophenyl)-3,5-dimethyl-4-nitroso-1H-pyrazole (5d): Greenish solid; Yield: 65%; mp 116-118 ºC; IR (KBr, cm^{-1}): 2920, 1546, 1513, 1349, 1225, 1098, 768, 718; ^{1}H NMR (400 MHz, $CDCl_3$): δ 2.44(brs, 3H), 2.76 (brs, 3H), 7.48-7.63 (m, 4H); ^{13}C NMR (100 MHz, $CDCl_3$): δ 10.56, 13.20, 128.18, 129.39, 130.71, 131.67, 132.02, 135.31, 160.13; ESI-HRMS (m/z) calculated. for $C_{11}H_{10}ClN_3O$: 235.0512; observed: 236.0581 $(MH)^+$, 238.0557 $(MH+2)^+$.

1-(3-Chlorophenyl)-3,5-dimethyl-4-nitroso-1H-pyrazole (5e): Greenish solid; Yield: 69%; mp 87-89 ºC; IR (KBr, cm^{-1}): 2922, 1591, 1544, 1350,1222, 1078, 777, 682; ^{1}H NMR (400 MHz, $CDCl_3$): δ 2.41 (s, 3H), 2.97 (s, 3H), 7.42-7.43 (m, 1H), 7.49-7.50 (m, 2H), 7.57 (s, 1H); ^{13}C NMR (100 MHz, $CDCl_3$): δ 11.27, 13.09, 123.12, 125.48, 129.46, 130.58, 135.43, 138.86, 160.65; ESI-HRMS (m/z) calculated for $C_{11}H_{10}ClN_3O$: 235.0512; observed: 236.0606 $(MH)^+$, 238.058 $(MH+2)^+$.

1-(2-Chlorophenyl)-3,5-dimethyl-4-nitroso-1H-pyrazole (5f) Greenish solid; Yield: 70%; mp 120-122 ºC; IR (KBr, cm^{-1}): 2927, 1543, 1508, 1344, 1235, 1085, 827, 769; ^{1}H NMR (400 MHz, $CDCl_3$): δ 2.40 (s, 3H), 2.95 (s, 3H), 7.47 (d, J = 8.70 Hz, 2H), 7.54 (d, J = 8.24 Hz, 2H); ^{13}C NMR (100 MHz, $CDCl_3$): δ 11.24, 13.10, 126.33, 129.84, 135.26, 136.32, 160.70; ESI-HRMS (m/z) calculated for $C_{11}H_{10}ClN_3O$: 235.0512; observed: 236.0597 $(MH)^+$, 238.0579 $(MH+2)^+$.

3,5-dimethyl-4-nitroso-1-(p-tolyl)-1H-pyrazole (5g): Greenish solid; Yield: 67%; mp 114-116 ºC; IR (KBr, cm^{-1}): 2926, 1541, 1517, 1342, 1232, 1080, 817; ^{1}H NMR (400 MHz, $CDCl_3$): δ 2.41-2.45 (m, 6H), 2.91 (brs, 3H), 7.33-7.39 (m, 4H); ^{13}C NMR (100 MHz, $CDCl_3$): δ 9.02, 10.95, 19.12, 122.82, 127.98, 133.09, 137.38, 158.58; ESI-HRMS (m/z) calculated for $C_{12}H_{13}N_3O$: 215.1059; observed: 216.2504 $(MH)^+$.

1-(4-Bromophenyl)-3,5-dimethyl-4-nitroso-1H-pyrazole (5h): Greenish solid; Yield: 62%; mp 118-120 ºC; IR (KBr, cm^{-1}): 2925, 1543, 1508, 1348, 1228, 1072, 769, 705; ^{1}H NMR (400 MHz, $CDCl_3$): δ 2.40 (brs, 3H), 2.96 (brs, 3H), 7.41 (d, J = 8.24 Hz, 2H), 7.70 (d, J = 8.70 Hz, 2H); ^{13}C NMR (100 MHz, $CDCl_3$): δ 11.26, 13.11, 123.25, 126.57, 132.82, 136.83, 160.71; ESI-HRMS (m/z) calculated for $C_{11}H_{10}BrN_3O$: 279.0007; observed: 280.0074 $(MH)^+$, 282.0058 $(MH+2)^+$.

4-(3,5-dimethyl-4-nitroso-1H-pyrazol-1-yl)benzonitrile (5i): Greenish solid; Yield: 65%; mp 126-128 ºC; IR (KBr, cm^{-1}): 2932, 2227, 1544, 1510, 1336, 1230, 839, 771; ^{1}H NMR (400 MHz, $CDCl_3$): δ 2.39 (s, 3H), 3.06 (s, 3H), 7.73 (d, J = 8.24 Hz, 2H), 7.87 (d, J = 8.24,, Hz, 2H); ^{13}C NMR (100 MHz, $CDCl_3$): δ 11.51, 13.14, 112.80, 117.78, 125.28, 133.59, 141.46, 160.62; ESI-HRMS (m/z) calculated for $C_{12}H_{10}N_4O$: 226.0855; observed: 227.0936 $(MH)^+$.

1-(3,4-dimethylphenyl)-3,5-dimethyl-4-nitroso-1H-pyrazole (5j): Greenish solid; Yield: 65%; mp 106-108 ºC; IR (KBr, cm^{-1}): 2922, 1541, 1514, 1323, 1172, 1043, 790; ^{1}H NMR (400 MHz, $CDCl_3$): δ 2.31-2.42 (m, 9H), 2.90 (brs, 3H), 7.19 (d, J = 7.79 Hz, 1H), 7.27-7.30 (m, 2H); ^{13}C NMR (100 MHz, $CDCl_3$): δ 11.01, 12.93, 19.46, 19.75, 122.15, 125.93, 126.03, 130.31, 135.26, 138.02, 138.16, 160.66; ESI-HRMS (m/z) calculated for $C_{13}H_{15}N_3O$: 229.1215; observed: 230.1285 $(MH)^+$.

1-(2,5-dichlorophenyl)-3,5-dimethyl-4-nitroso-1H-pyrazole (5k): Greenish solid; Yield: 67%; mp 98-100 ºC; IR (KBr, cm^{-1}): 2926, 1541, 1465, 1344, 1255, 1095, 817, 777; ^{1}H NMR (400 MHz, $CDCl_3$):

δ 2.44 (s, 3H), 2.77 (s, 3H), 7.49-7.57 (m, 3H); ^{13}C NMR (100 MHz, $CDCl_3$): δ 10.58, 13.13, 129.66, 130.42, 131.50, 131.80, 133.82, 136.16, 159.93; ESI-HRMS (m/z) calculated for $C_{11}H_9Cl_2N_3O$: 269.0123; observed: 270.0339 $(MH)^+$, 272.0264 $(MH+2)^+$, 274.0155$(MH+4)^+$.

Procedure for the synthesis of 3,5-dimethyl-1-phenyl-1H-pyrazol-4-amine (6).

Compound 3,5-dimethyl-4-nitroso-1-phenyl-1*H*-pyrazole (500 mg, 2.48 mmol) was dissolved in methanol (5 mL) and hydrogen gas was passed in the presence of Pd/C (50 mg) at 60 psi pressure followed by shaking for 3-4 hrs at room temperature. The completion of reaction was determined by TLC, palladium was removed by filtering through celite pad and followed by washing with methanol. The filtrate obtained was combined and dried using rotavapor at reduced pressure. Subsequently, 50 mL diluted HCL was added to the crude product with stirring. The impurities were removed by the addition of ethyl acetate, the-aqueous layer containing final product was neutralised with saturated K_2CO_3 solution and finally isolated by extraction with ethyl acetate.

3,5-dimethyl-1-phenyl-1H-pyrazol-4-amine (6): Yellow solid; Yield: 85%; mp 64-66 ºC 67 ºC; IR (KBr, cm^{-1}): 3323, 2921, 1598, 1438, 1368, 1215, 1074, 766; ^{1}H NMR (400 MHz, $CDCl_3$): δ 2.20 (s, 3H), 2.22 (s, 3H), 2.57 (brs, 2H), 7.24-7.42 (m, 5H); ^{13}C NMR (100 MHz, $CDCl_3$): δ 10.41, 11.00, 124.20, 124.94, 126.81, 127.14, 129.08, 140.28, 140.98; ESI-HRMS (m/z) calculated for $C_{11}H_{13}N_3$: 187.1109; observed: 188.1328 $(MH)^+$.

Procedure for the synthesis of 4-bromo/chloro-3,5-dimethyl-1-phenyl-1H-pyrazole (7a-b).

The synthesized compound 3,5-dimethyl-1-phenyl-1*H*-pyrazole (300 mg, 1.74 mmol) and N-halo-succinimide (1.74 mmol) were taken in a round bottom flask containing 5 mL of water. The reaction mixture containing N-bromosuccinimide or N-chlorosuccinimide was stirred at room temperature or at 37 ˚C, respectively for 2 hrs. The completion of reaction was confirmed by TLC and the product was extracted with ethyl acetate multiple times. The combined organic layer was washed with water and brine, dried by the addition of Na_2SO_4 and ethyl acetate to purify the desired products.

4-Bromo-3,5-dimethyl-1-phenyl-1H-pyrazole (7a): Yellow oil; Yield: 75%; IR (KBr, cm^{-1}): 2982, 1597, 1502, 1373, 1216, 1080, 744, 691; ^{1}H NMR (400 MHz, $CDCl_3$): δ 2.30 (s, 6H), 7.38-7.41 (m, 3H), 7.45-7.47 (m, 2H); ^{13}C NMR (100 MHz, $CDCl_3$): δ 11.69, 12.29, 96.07, 124.60, 127.75, 129.10, 137.44, 139.70, 147.50; ESI-HRMS (m/z) calculated for $C_{11}H_{11}BrN_2$: 250.0106; observed: 251.0179 $(MH)^+$, 253.0159 $(MH+2)^+$.

4-Chloro-3,5-dimethyl-1-phenyl-1H-pyrazole (7b): Yellow oil; Yield: 75%; IR (KBr, cm^{-1}): 2923, 1596, 1501, 1374, 1272, 1099, 760, 692; ^{1}H NMR (400 MHz, $CDCl_3$): δ 2.30 (s, 6H), 7.37-7.46 (m, 5H); ^{13}C NMR (100 MHz, $CDCl_3$): δ 10.91, 11.49, 109.89, 124.62, 127.81, 129.26, 135.79, 139.82, 146.14; ESI-HRMS (m/z) calculated for $C_{11}H_{11}ClN_2$: 206.0611; observed: 207.0930 $(MH)^+$, 209.0757 $(MH+2)^+$.

Characterization details of various scaffolds prepared in the present study.

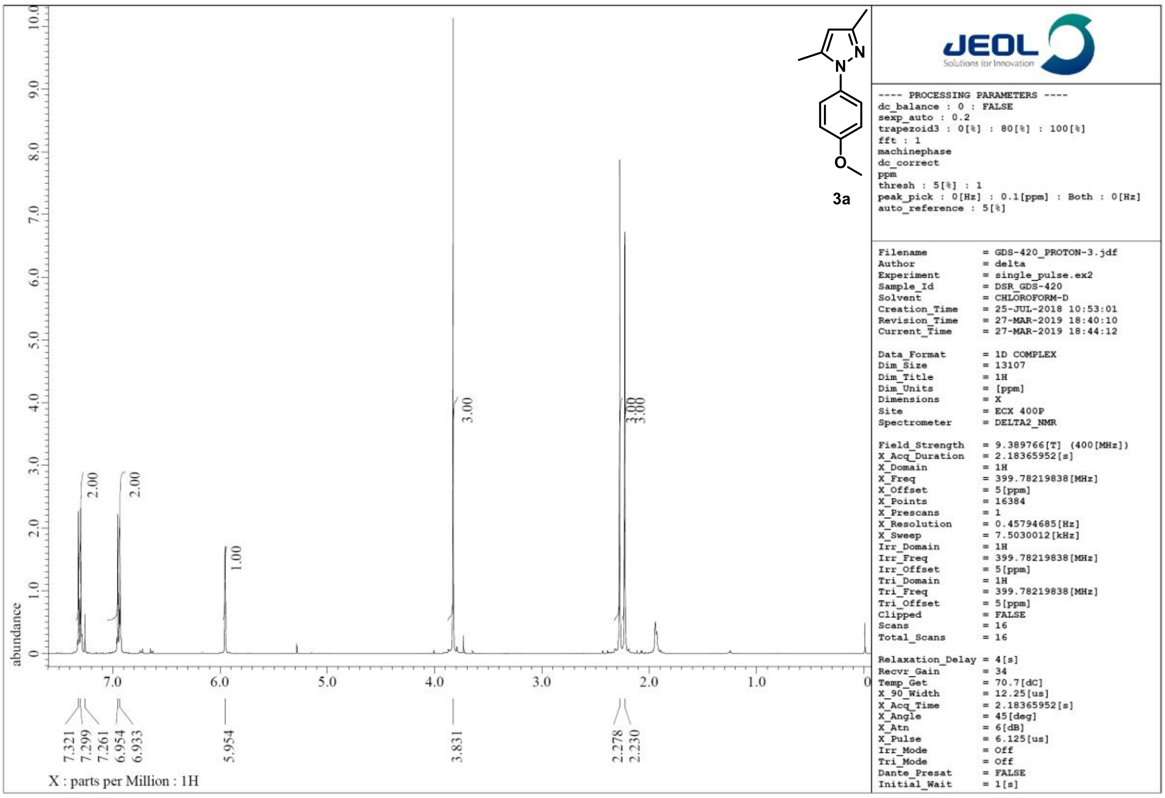

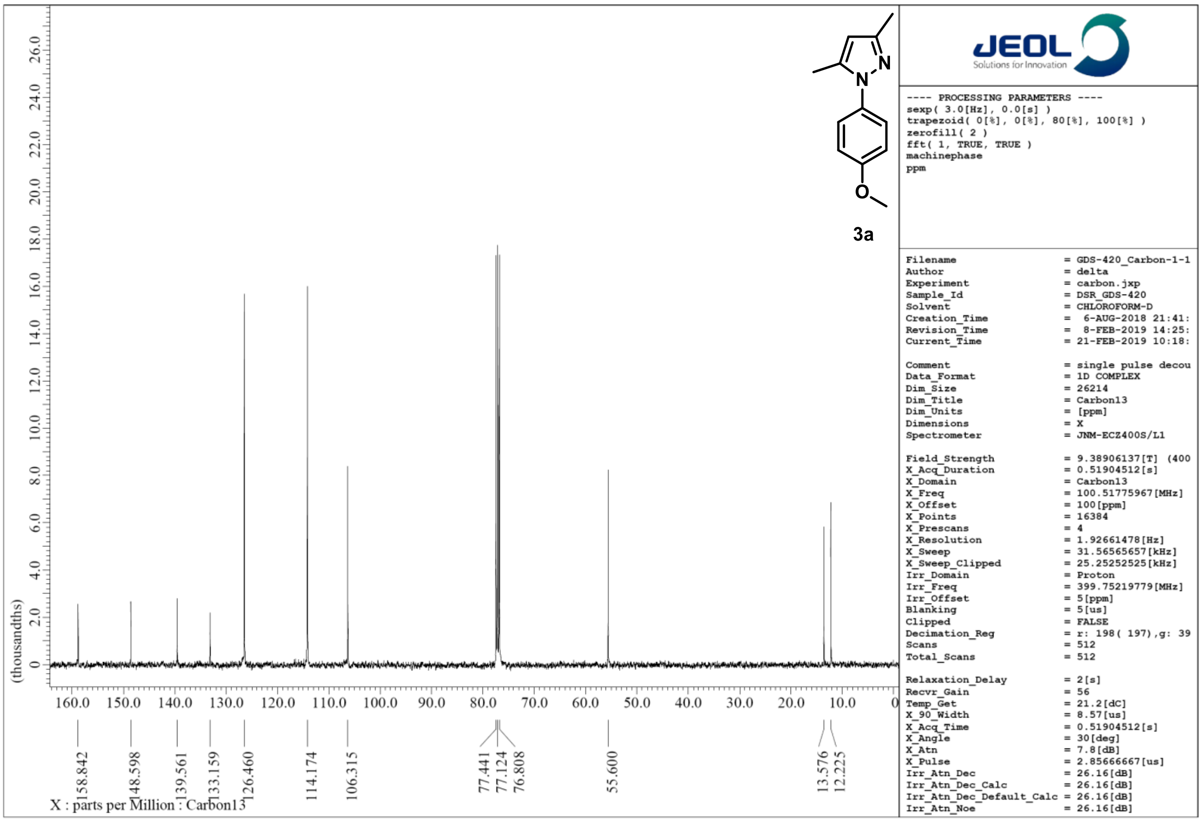

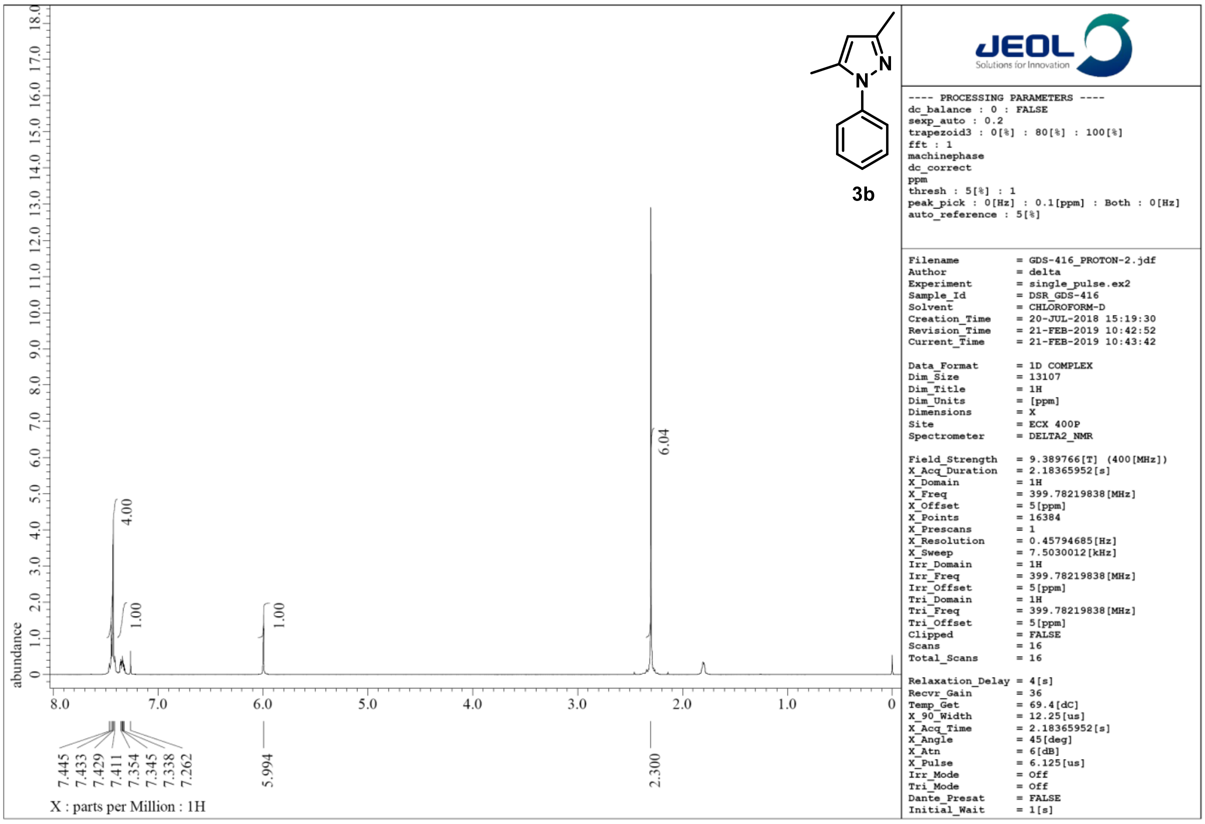
N
N
3b
abundance
0 1.0 2.0 3.0 4.0 5.0 6.0 7.0 8.0 9.0 10.0 11.0 12.0 13.0 14.0 15.0 16.0 17.0 18.0
4.00
1.00
1.00
6.04
8.0 7.0 6.0 5.0 4.0 3.0 2.0 1.0 0
7.445
7.433
7.429
7.411
7.354
7.345
7.338
7.262
5.994
2.300
X : parts per Million : 1H
---- PROCESSING PARAMETERS ----
dc_balance : 0 : FALSE
sexp_auto : 0.2
trapezoid3 : 0[%] : 80[%] : 100[%]
fft : 1
machinephase
dc_correct
ppm
thresh : 5[%] : 1
peak_pick : 0[Hz] : 0.1[ppm] : Both : 0[Hz]
auto_reference : 5[%]
Filename = GDS-416_PROTON-2.jdf
Author = delta
Experiment = single_pulse.ex2
Sample_Id = DSR_GDS-416
Solvent = CHLOROFORM-D
Creation_Time = 20-JUL-2018 15:19:30
Revision_Time = 21-FEB-2019 10:42:52
Current_Time = 21-FEB-2019 10:43:42
Data_Format = 1D COMPLEX
Dim_Size = 13107
Dim_Title = 1H
Dim_Units = [ppm]
Dimensions = X
Site = ECX 400P
Spectrometer = DELTA2_NMR
Field_Strength = 9.389766[T] (400[MHz])
X_Acq_Duration = 2.18365952[s]
X_Domain = 1H
X_Freq = 399.78219838[MHz]
X_Offset = 5[ppm]
X_Points = 16384
X_Prescans = 1
X_Resolution = 0.45794685[Hz]
X_Sweep = 7.5030012[kHz]
Irr_Domain = 1H
Irr_Freq = 399.78219838[MHz]
Irr_Offset = 5[ppm]
Tri_Domain = 1H
Tri_Freq = 399.78219838[MHz]
Tri_Offset = 5[ppm]
Clipped = FALSE
Scans = 16
Total_Scans = 16
Relaxation_Delay = 4[s]
Recvr_Gain = 36
Temp_Get = 69.4[dC]
X_90_Width = 12.25[us]
X_Acq_Time = 2.18365952[s]
X_Angle = 45[deg]
X_Atn = 6[dB]
X_Pulse = 6.125[us]
Irr_Mode = Off
Tri_Mode = Off
Dante_Presat = FALSE
Initial_Wait = 1[s]

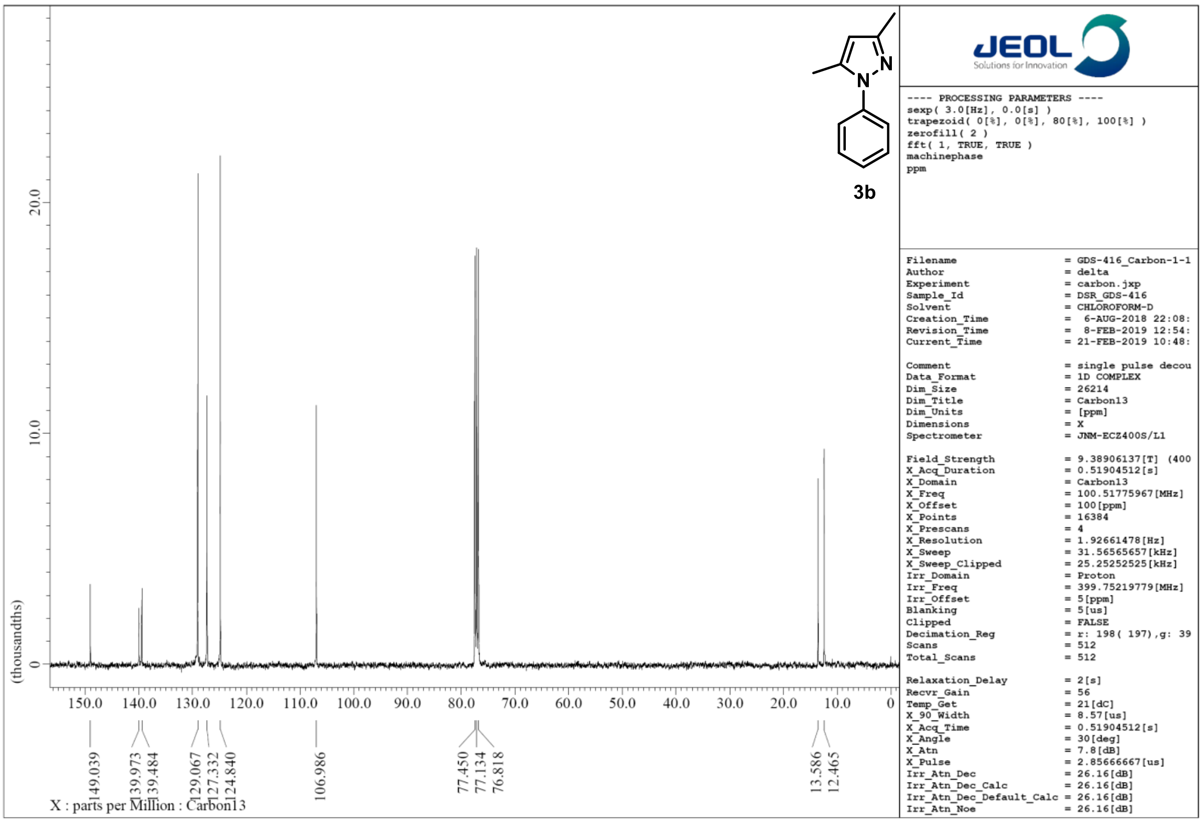
N
N
3b
(thousandths)
0
10.0
20.0
150.0 140.0 130.0 120.0 110.0 100.0 90.0 80.0 70.0 60.0 50.0 40.0 30.0 20.0 10.0 0
149.039
139.973
139.484
129.067
127.332
124.840
106.986
77.450
77.134
76.818
13.586
12.465
X : parts per Million : Carbon13
---- PROCESSING PARAMETERS ----
sexp(3.0[Hz], 0.0[s])
trapezoid(0[%], 0[%], 80[%], 100[%])
zerofill(2)
fft(1, TRUE, TRUE)
machinephase
ppm
Filename = GDS-416_Carbon-1-1
Author = delta
Experiment = carbon.jxp
Sample_Id = DSR_GDS-416
Solvent = CHLOROFORM-D
Creation_Time = 6-AUG-2018 22:08:
Revision_Time = 8-FEB-2019 12:54:
Current_Time = 21-FEB-2019 10:48:
Comment = single pulse decou
Data_Format = 1D COMPLEX
Dim_Size = 26214
Dim_Title = Carbon13
Dim_Units = [ppm]
Dimensions = X
Spectrometer = JNM-ECZ400S/L1
Field_Strength = 9.38906137[T] (400
X_Acq_Duration = 0.51904512[s]
X_Domain = Carbon13
X_Freq = 100.51775967[MHz]
X_Offset = 100[ppm]
X_Points = 16384
X_Prescans = 4
X_Resolution = 1.92661478[Hz]
X_Sweep = 31.56565657[kHz]
X_Sweep_Clipped = 25.25252525[kHz]
Irr_Domain = Proton
Irr_Freq = 399.75219779[MHz]
Irr_Offset = 5[ppm]
Blanking = 5[us]
Clipped = FALSE
Decimation_Reg = r: 198(197),g: 39
Scans = 512
Total_Scans = 512
Relaxation_Delay = 2[s]
Recvr_Gain = 56
Temp_Get = 21[dC]
X_90_Width = 8.57[us]
X_Acq_Time = 0.51904512[s]
X_Angle = 30[deg]
X_Atn = 7.8[dB]
X_Pulse = 2.85666667[us]
Irr_Atn_Dec = 26.16[dB]
Irr_Atn_Dec_Calc = 26.16[dB]
Irr_Atn_Dec_Default_Calc = 26.16[dB]
Irr_Atn_Noe = 26.16[dB]

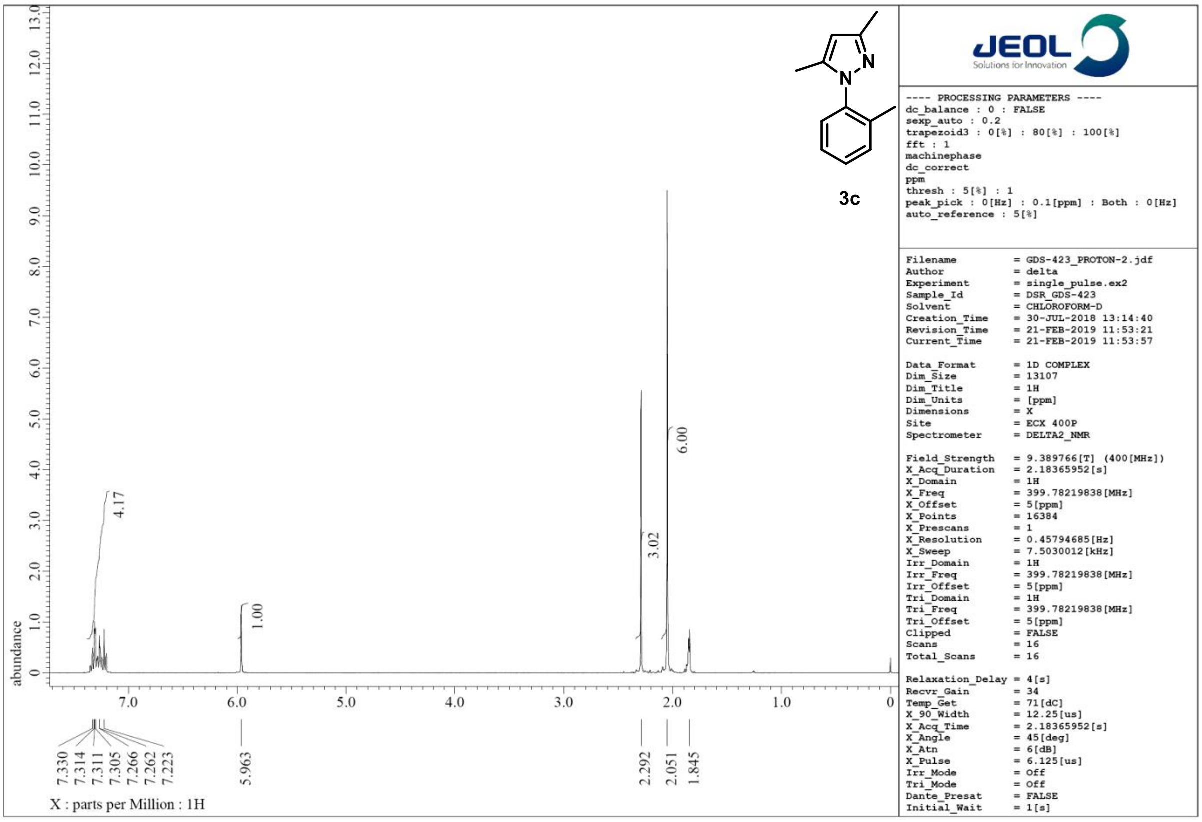
3c
JEOL
Solutions for Innovation
---- PROCESSING PARAMETERS ----
dc_balance : 0 : FALSE
sexp_auto : 0.2
trapezoid3 : 0[%] : 80[%] : 100[%]
fft : 1
machinephase
dc_correct
ppm
thresh : 5[%] : 1
peak_pick : 0[Hz] : 0.1[ppm] : Both : 0[Hz]
auto_reference : 5[%]
Filename = GDS-423_PROTON-2.jdf
Author = delta
Experiment = single_pulse.ex2
Sample_Id = DSR_GDS-423
Solvent = CHLOROFORM-D
Creation_Time = 30-JUL-2018 13:14:40
Revision_Time = 21-FEB-2019 11:53:21
Current_Time = 21-FEB-2019 11:53:57
Data_Format = 1D COMPLEX
Dim_Size = 13107
Dim_Title = 1H
Dim_Units = [ppm]
Dimensions = X
Site = ECX 400P
Spectrometer = DELTA2_NMR
Field_Strength = 9.389766[T] (400[MHz])
X_Acq_Duration = 2.18365952[s]
X_Domain = 1H
X_Freq = 399.78219838[MHz]
X_Offset = 5[ppm]
X_Points = 16384
X_Prescans = 1
X_Resolution = 0.45794685[Hz]
X_Sweep = 7.5030012[kHz]
Irr_Domain = 1H
Irr_Freq = 399.78219838[MHz]
Irr_Offset = 5[ppm]
Tri_Domain = 1H
Tri_Freq = 399.78219838[MHz]
Tri_Offset = 5[ppm]
Clipped = FALSE
Scans = 16
Total_Scans = 16
Relaxation_Delay = 4[s]
Recvr_Gain = 34
Temp_Get = 71[dC]
X_90_Width = 12.25[us]
X_Acq_Time = 2.18365952[s]
X_Angle = 45[deg]
X_Atn = 6[dB]
X_Pulse = 6.125[us]
Irr_Mode = Off
Tri_Mode = Off
Dante_Presat = FALSE
Initial_Wait = 1[s]
abundance
4.17
1.00
3.02
6.00
7.330
7.314
7.311
7.305
7.266
7.262
7.223
5.963
2.292
2.051
1.845
X : parts per Million : 1H

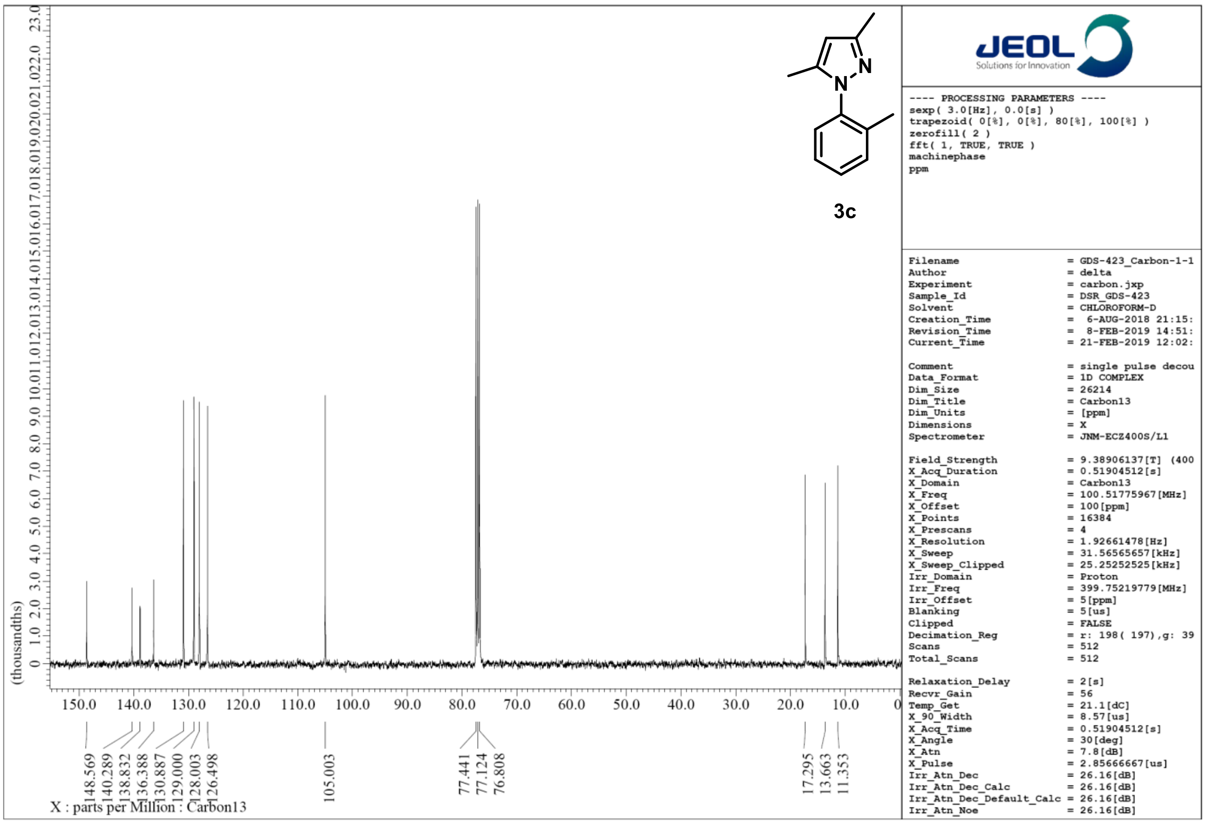
3c
JEOL
Solutions for Innovation
---- PROCESSING PARAMETERS ----
sexp(3.0[Hz], 0.0[s])
trapezoid(0[%], 0[%], 80[%], 100[%])
zerofill(2)
fft(1, TRUE, TRUE)
machinephase
ppm
Filename = GDS-423_Carbon-1-1
Author = delta
Experiment = carbon.jxp
Sample_Id = DSR_GDS-423
Solvent = CHLOROFORM-D
Creation_Time = 6-AUG-2018 21:15:
Revision_Time = 8-FEB-2019 14:51:
Current_Time = 21-FEB-2019 12:02:
Comment = single pulse decou
Data_Format = 1D COMPLEX
Dim_Size = 26214
Dim_Title = Carbon13
Dim_Units = [ppm]
Dimensions = X
Spectrometer = JNM-ECZ400S/L1
Field_Strength = 9.38906137[T] (400
X_Acq_Duration = 0.51904512[s]
X_Domain = Carbon13
X_Freq = 100.51775967[MHz]
X_Offset = 100[ppm]
X_Points = 16384
X_Prescans = 4
X_Resolution = 1.92661478[Hz]
X_Sweep = 31.56565657[kHz]
X_Sweep_Clipped = 25.25252525[kHz]
Irr_Domain = Proton
Irr_Freq = 399.75219779[MHz]
Irr_Offset = 5[ppm]
Blanking = 5[us]
Clipped = FALSE
Decimation_Reg = r: 198(197),g: 39
Scans = 512
Total_Scans = 512
Relaxation_Delay = 2[s]
Recvr_Gain = 56
Temp_Get = 21.1[dC]
X_90_Width = 8.57[us]
X_Acq_Time = 0.51904512[s]
X_Angle = 30[deg]
X_Atn = 7.8[dB]
X_Pulse = 2.85666667[us]
Irr_Atn_Dec = 26.16[dB]
Irr_Atn_Dec_Calc = 26.16[dB]
Irr_Atn_Dec_Default_Calc = 26.16[dB]
Irr_Atn_Noe = 26.16[dB]
(thousandths)
148.569
140.289
138.832
136.388
130.887
129.000
128.003
126.498
105.003
77.441
77.124
76.808
17.295
13.663
11.353
X : parts per Million : Carbon13

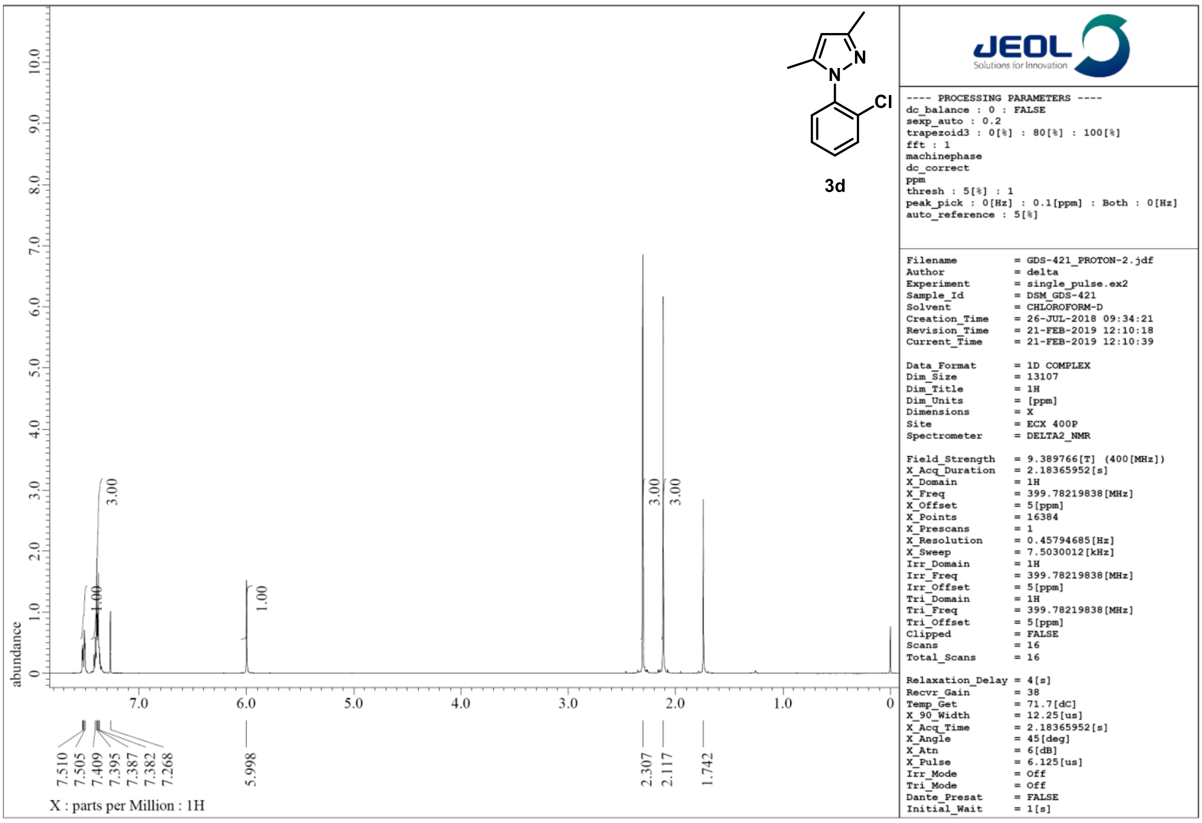

3d
Cl
N
---- PROCESSING PARAMETERS ----
dc_balance : 0 : FALSE
sexp_auto : 0.2
trapezoid3 : 0[%] : 80[%] : 100[%]
fft : 1
machinephase
dc_correct
ppm
thresh : 5[%] : 1
peak_pick : 0[Hz] : 0.1[ppm] : Both : 0[Hz]
auto_reference : 5[%]
Filename = GDS-421_PROTON-2.jdf
Author = delta
Experiment = single_pulse.ex2
Sample_Id = DSM_GDS-421
Solvent = CHLOROFORM-D
Creation_Time = 26-JUL-2018 09:34:21
Revision_Time = 21-FEB-2019 12:10:18
Current_Time = 21-FEB-2019 12:10:39
Data_Format = 1D COMPLEX
Dim_Size = 13107
Dim_Title = 1H
Dim_Units = [ppm]
Dimensions = X
Site = ECX 400P
Spectrometer = DELTA2_NMR
Field_Strength = 9.389766[T] (400[MHz])
X_Acq_Duration = 2.18365952[s]
X_Domain = 1H
X_Freq = 399.78219838[MHz]
X_Offset = 5[ppm]
X_Points = 16384
X_Prescans = 1
X_Resolution = 0.45794685[Hz]
X_Sweep = 7.5030012[kHz]
Irr_Domain = 1H
Irr_Freq = 399.78219838[MHz]
Irr_Offset = 5[ppm]
Tri_Domain = 1H
Tri_Freq = 399.78219838[MHz]
Tri_Offset = 5[ppm]
Clipped = FALSE
Scans = 16
Total_Scans = 16
Relaxation_Delay = 4[s]
Recvr_Gain = 38
Temp_Get = 71.7[dC]
X_90_Width = 12.25[us]
X_Acq_Time = 2.18365952[s]
X_Angle = 45[deg]
X_Atn = 6[dB]
X_Pulse = 6.125[us]
Irr_Mode = Off
Tri_Mode = Off
Dante_Presat = FALSE
Initial_Wait = 1[s]
abundance
10.0
9.0
8.0
7.0
6.0
5.0
4.0
3.0
2.0
1.0
0
3.00
1.00
1.00
3.00
3.00
7.0
6.0
5.0
4.0
3.0
2.0
1.0
0
7.510
7.505
7.409
7.395
7.387
7.382
7.268
5.998
2.307
2.117
1.742
X : parts per Million : 1H

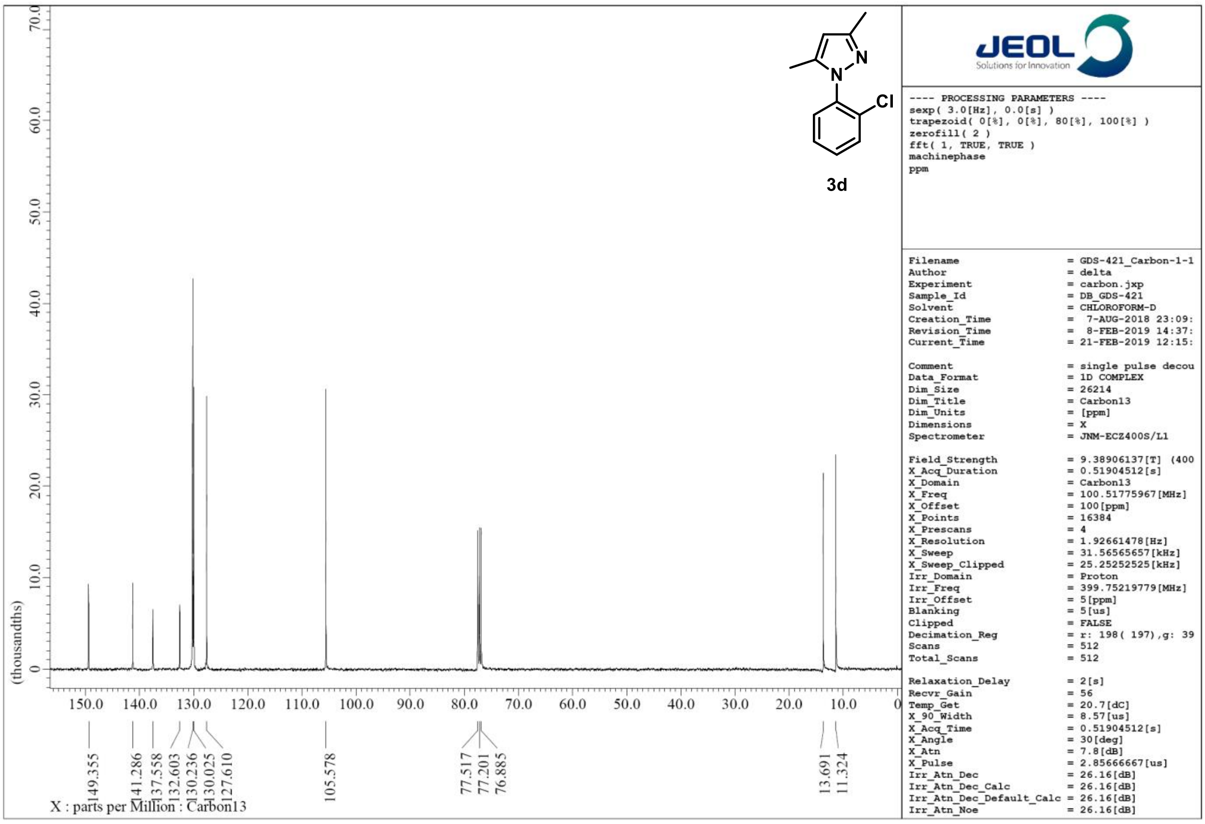

3d
Cl
N
---- PROCESSING PARAMETERS ----
sexp(3.0[Hz], 0.0[s])
trapezoid(0[%], 0[%], 80[%], 100[%])
zerofill(2)
fft(1, TRUE, TRUE)
machinephase
ppm
Filename = GDS-421_Carbon-1-1
Author = delta
Experiment = carbon.jxp
Sample_Id = DB_GDS-421
Solvent = CHLOROFORM-D
Creation_Time = 7-AUG-2018 23:09:
Revision_Time = 8-FEB-2019 14:37:
Current_Time = 21-FEB-2019 12:15:
Comment = single pulse decou
Data_Format = 1D COMPLEX
Dim_Size = 26214
Dim_Title = Carbon13
Dim_Units = [ppm]
Dimensions = X
Spectrometer = JNM-ECZ400S/L1
Field_Strength = 9.38906137[T] (400
X_Acq_Duration = 0.51904512[s]
X_Domain = Carbon13
X_Freq = 100.51775967[MHz]
X_Offset = 100[ppm]
X_Points = 16384
X_Prescans = 4
X_Resolution = 1.92661478[Hz]
X_Sweep = 31.56565657[kHz]
X_Sweep_Clipped = 25.25252525[kHz]
Irr_Domain = Proton
Irr_Freq = 399.75219779[MHz]
Irr_Offset = 5[ppm]
Blanking = 5[us]
Clipped = FALSE
Decimation_Reg = r: 198(197),g: 39
Scans = 512
Total_Scans = 512
Relaxation_Delay = 2[s]
Recvr_Gain = 56
Temp_Get = 20.7[dC]
X_90_Width = 8.57[us]
X_Acq_Time = 0.51904512[s]
X_Angle = 30[deg]
X_Atn = 7.8[dB]
X_Pulse = 2.85666667[us]
Irr_Atn_Dec = 26.16[dB]
Irr_Atn_Dec_Calc = 26.16[dB]
Irr_Atn_Dec_Default_Calc = 26.16[dB]
Irr_Atn_Noe = 26.16[dB]
(thousandths)
70.0
60.0
50.0
40.0
30.0
20.0
10.0
0
150.0
140.0
130.0
120.0
110.0
100.0
90.0
80.0
70.0
60.0
50.0
40.0
30.0
20.0
10.0
0
149.355
141.286
137.558
132.603
130.236
130.025
127.610
105.578
77.517
77.201
76.885
13.691
11.324
X : parts per Million : Carbon13

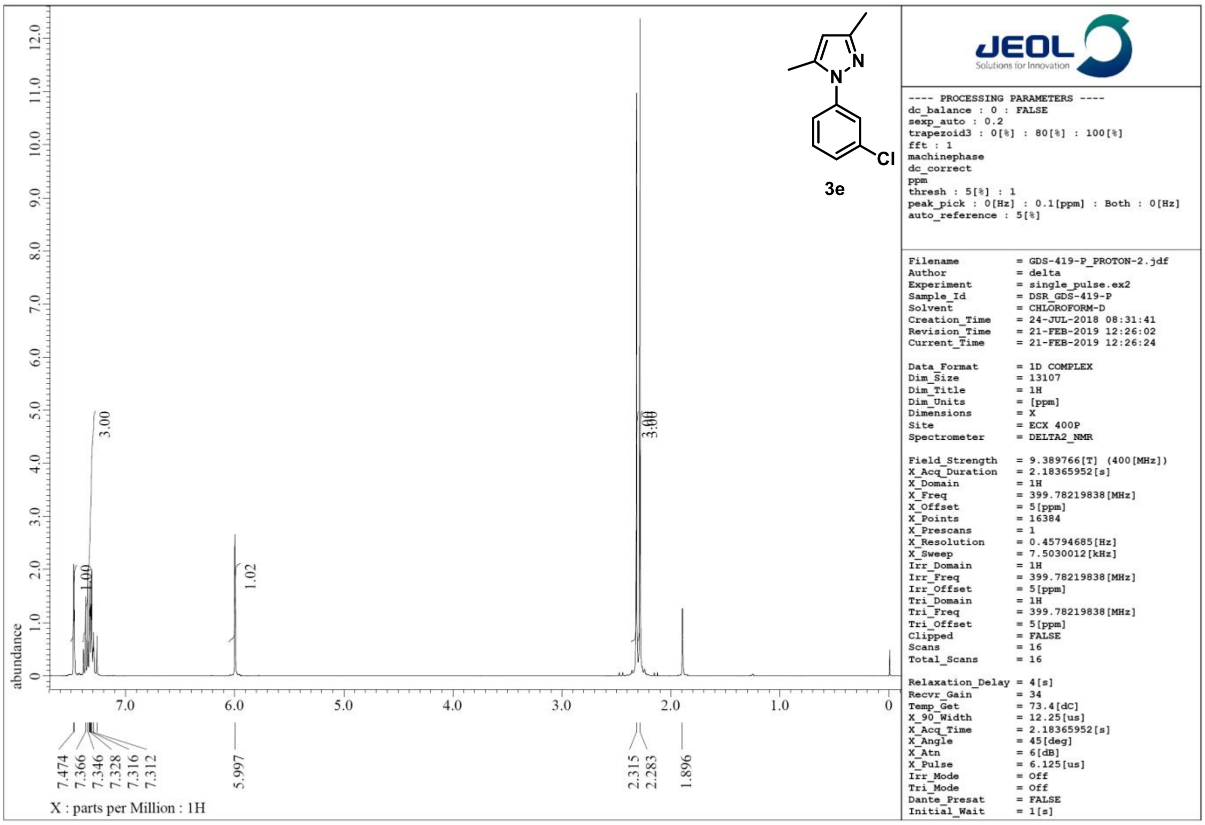
3e
Cl
N
N
abundance
7.474
7.366
7.346
7.328
7.316
7.312
5.997
2.315
2.283
1.896
3.00
1.00
1.02
3.00
X : parts per Million : 1H
---- PROCESSING PARAMETERS ----
dc_balance : 0 : FALSE
sexp_auto : 0.2
trapezoid3 : 0[%] : 80[%] : 100[%]
fft : 1
machinephase
dc_correct
ppm
thresh : 5[%] : 1
peak_pick : 0[Hz] : 0.1[ppm] : Both : 0[Hz]
auto_reference : 5[%]
Filename = GDS-419-P_PROTON-2.jdf
Author = delta
Experiment = single_pulse.ex2
Sample_Id = DSR_GDS-419-P
Solvent = CHLOROFORM-D
Creation_Time = 24-JUL-2018 08:31:41
Revision_Time = 21-FEB-2019 12:26:02
Current_Time = 21-FEB-2019 12:26:24
Data_Format = 1D COMPLEX
Dim_Size = 13107
Dim_Title = 1H
Dim_Units = [ppm]
Dimensions = X
Site = ECX 400P
Spectrometer = DELTA2_NMR
Field_Strength = 9.389766[T] (400[MHz])
X_Acq_Duration = 2.18365952[s]
X_Domain = 1H
X_Freq = 399.78219838[MHz]
X_Offset = 5[ppm]
X_Points = 16384
X_Prescans = 1
X_Resolution = 0.45794685[Hz]
X_Sweep = 7.5030012[kHz]
Irr_Domain = 1H
Irr_Freq = 399.78219838[MHz]
Irr_Offset = 5[ppm]
Tri_Domain = 1H
Tri_Freq = 399.78219838[MHz]
Tri_Offset = 5[ppm]
Clipped = FALSE
Scans = 16
Total_Scans = 16
Relaxation_Delay = 4[s]
Recvr_Gain = 34
Temp_Get = 73.4[dC]
X_90_Width = 12.25[us]
X_Acq_Time = 2.18365952[s]
X_Angle = 45[deg]
X_Atn = 6[dB]
X_Pulse = 6.125[us]
Irr_Mode = Off
Tri_Mode = Off
Dante_Presat = FALSE
Initial_Wait = 1[s]

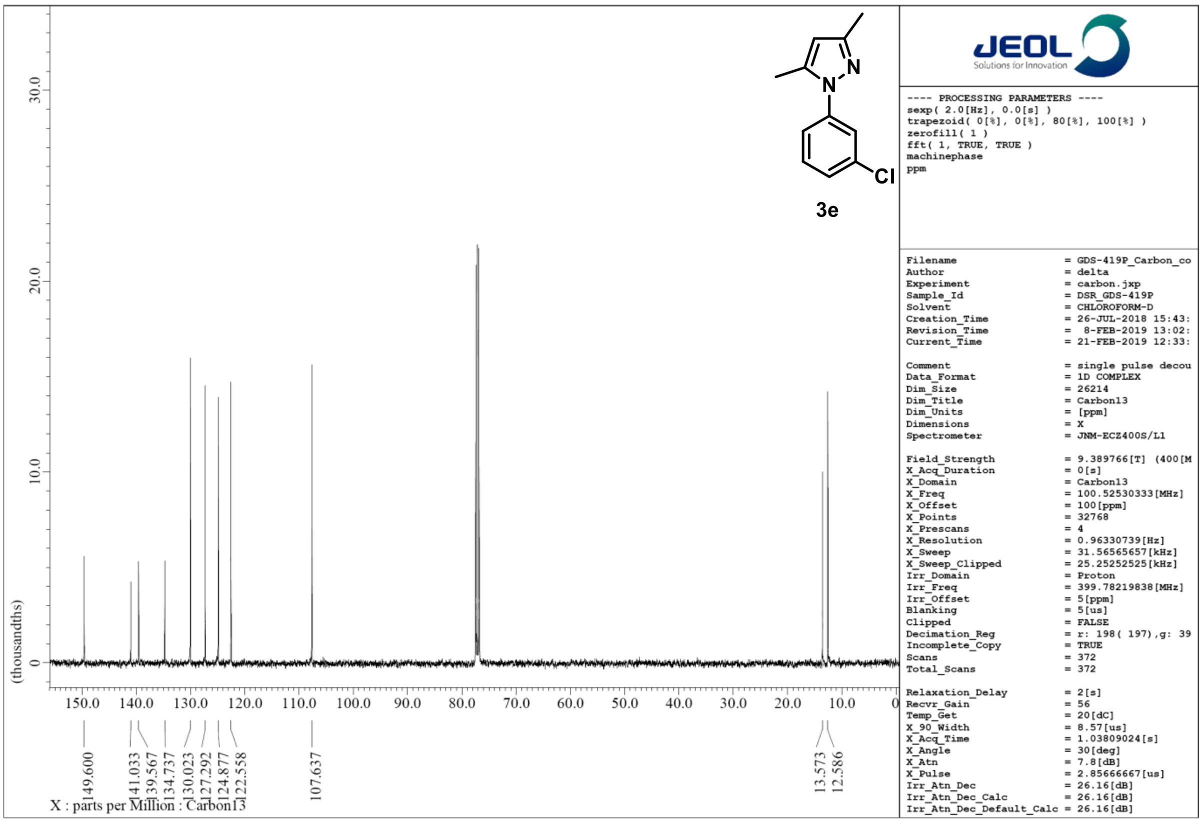
3e
Cl
N
N
(thousandths)
149.600
141.033
139.567
134.737
130.023
127.292
124.877
122.558
107.637
13.573
12.586
X : parts per Million : Carbon13
---- PROCESSING PARAMETERS ----
sexp(2.0[Hz], 0.0[s])
trapezoid(0[%], 0[%], 80[%], 100[%])
zerofill(1)
fft(1, TRUE, TRUE)
machinephase
ppm
Filename = GDS-419P_Carbon_co
Author = delta
Experiment = carbon.jxp
Sample_Id = DSR_GDS-419P
Solvent = CHLOROFORM-D
Creation_Time = 26-JUL-2018 15:43:
Revision_Time = 8-FEB-2019 13:02:
Current_Time = 21-FEB-2019 12:33:
Comment = single pulse decou
Data_Format = 1D COMPLEX
Dim_Size = 26214
Dim_Title = Carbon13
Dim_Units = [ppm]
Dimensions = X
Spectrometer = JNM-ECZ400S/L1
Field_Strength = 9.389766[T] (400[M
X_Acq_Duration = 0[s]
X_Domain = Carbon13
X_Freq = 100.52530333[MHz]
X_Offset = 100[ppm]
X_Points = 32768
X_Prescans = 4
X_Resolution = 0.96330739[Hz]
X_Sweep = 31.56565657[kHz]
X_Sweep_Clipped = 25.25252525[kHz]
Irr_Domain = Proton
Irr_Freq = 399.78219838[MHz]
Irr_Offset = 5[ppm]
Blanking = 5[us]
Clipped = FALSE
Decimation_Reg = r: 198(197),g: 39
Incomplete_Copy = TRUE
Scans = 372
Total_Scans = 372
Relaxation_Delay = 2[s]
Recvr_Gain = 56
Temp_Get = 20[dC]
X_90_Width = 8.57[us]
X_Acq_Time = 1.03809024[s]
X_Angle = 30[deg]
X_Atn = 7.8[dB]
X_Pulse = 2.85666667[us]
Irr_Atn_Dec = 26.16[dB]
Irr_Atn_Dec_Calc = 26.16[dB]
Irr_Atn_Dec_Default_Calc = 26.16[dB]

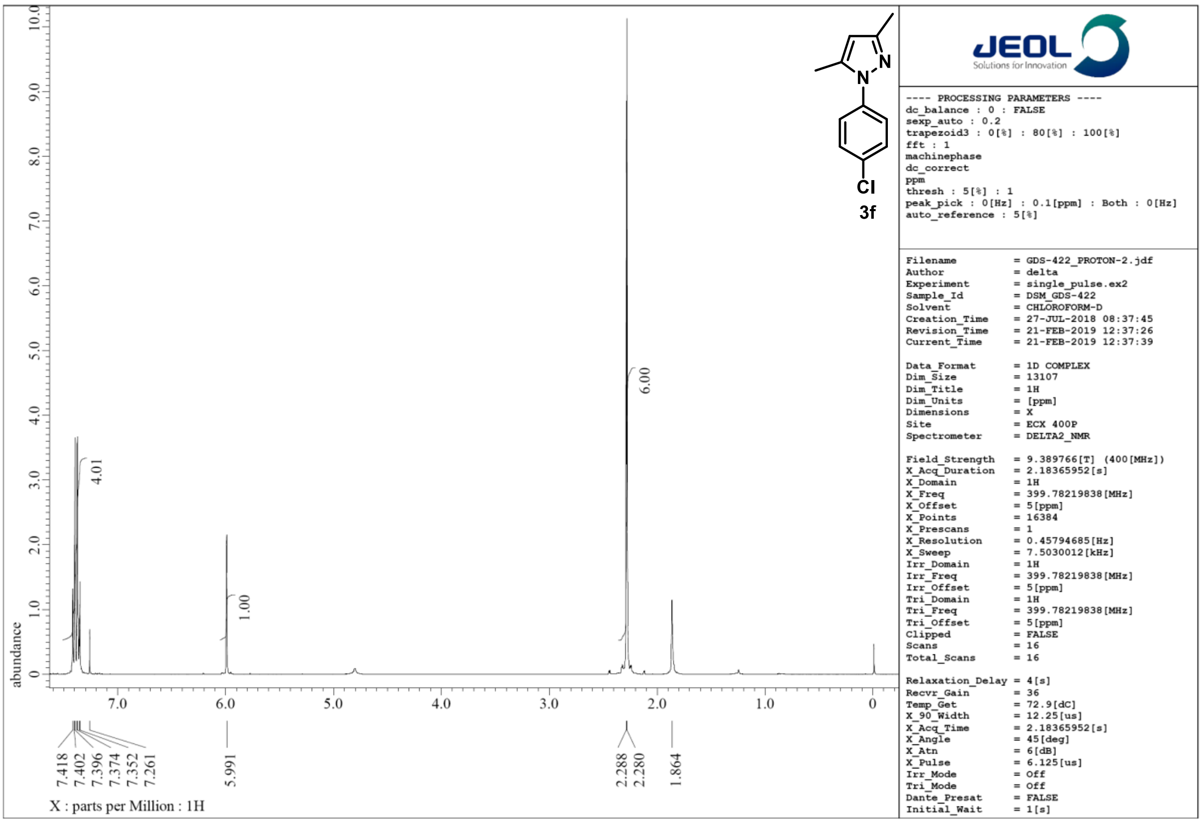
3f
Cl
N
N
JEOL
Solutions for Innovation
---- PROCESSING PARAMETERS ----
dc_balance : 0 : FALSE
sexp_auto : 0.2
trapezoid3 : 0[%] : 80[%] : 100[%]
fft : 1
machinephase
dc_correct
ppm
thresh : 5[%] : 1
peak_pick : 0[Hz] : 0.1[ppm] : Both : 0[Hz]
auto_reference : 5[%]
Filename = GDS-422_PROTON-2.jdf
Author = delta
Experiment = single_pulse.ex2
Sample_Id = DSM_GDS-422
Solvent = CHLOROFORM-D
Creation_Time = 27-JUL-2018 08:37:45
Revision_Time = 21-FEB-2019 12:37:26
Current_Time = 21-FEB-2019 12:37:39
Data_Format = 1D COMPLEX
Dim_Size = 13107
Dim_Title = 1H
Dim_Units = [ppm]
Dimensions = X
Site = ECX 400P
Spectrometer = DELTA2_NMR
Field_Strength = 9.389766[T] (400[MHz])
X_Acq_Duration = 2.18365952[s]
X_Domain = 1H
X_Freq = 399.78219838[MHz]
X_Offset = 5[ppm]
X_Points = 16384
X_Prescans = 1
X_Resolution = 0.45794685[Hz]
X_Sweep = 7.5030012[kHz]
Irr_Domain = 1H
Irr_Freq = 399.78219838[MHz]
Irr_Offset = 5[ppm]
Tri_Domain = 1H
Tri_Freq = 399.78219838[MHz]
Tri_Offset = 5[ppm]
Clipped = FALSE
Scans = 16
Total_Scans = 16
Relaxation_Delay = 4[s]
Recvr_Gain = 36
Temp_Get = 72.9[dC]
X_90_Width = 12.25[us]
X_Acq_Time = 2.18365952[s]
X_Angle = 45[deg]
X_Atn = 6[dB]
X_Pulse = 6.125[us]
Irr_Mode = Off
Tri_Mode = Off
Dante_Presat = FALSE
Initial_Wait = 1[s]
abundance
10.0
9.0
8.0
7.0
6.0
5.0
4.0
3.0
2.0
1.0
0
4.01
1.00
6.00
7.0
6.0
5.0
4.0
3.0
2.0
1.0
0
7.418
7.402
7.396
7.374
7.352
7.261
5.991
2.288
2.280
1.864
X : parts per Million : 1H

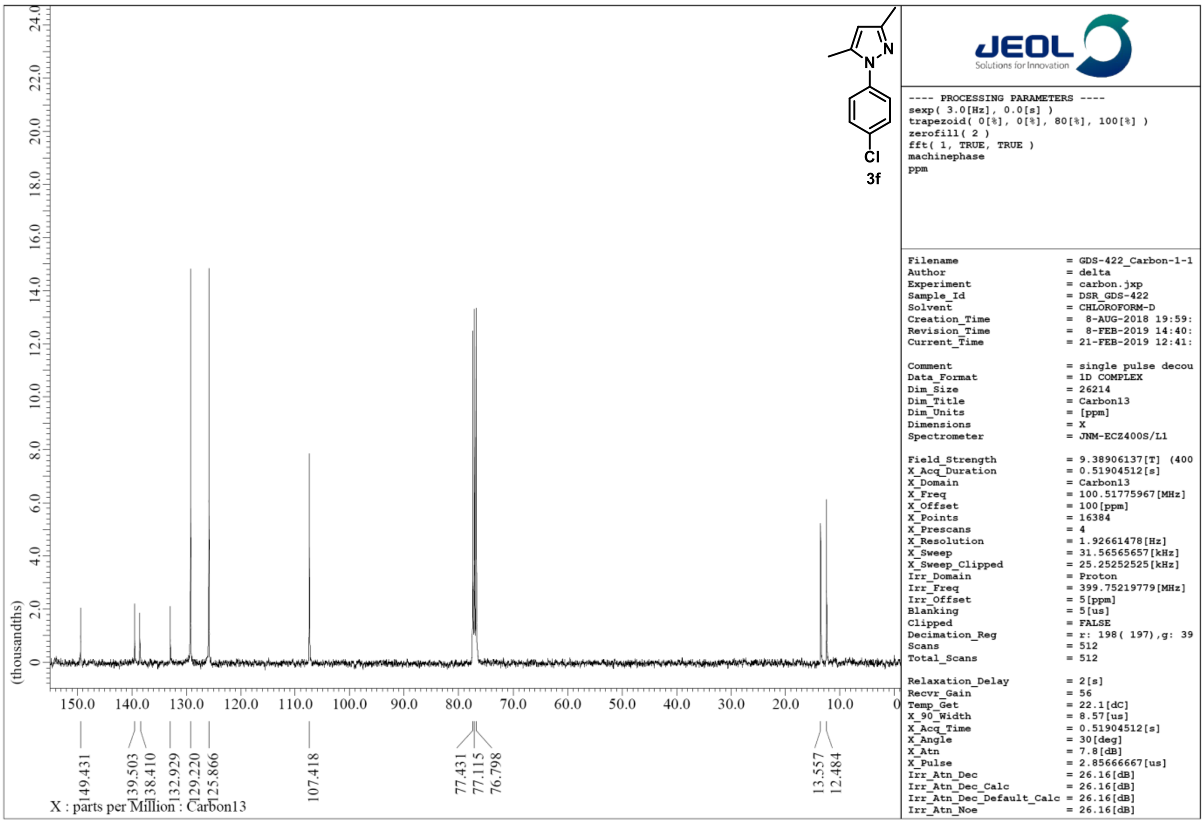
3f
Cl
N
N
JEOL
Solutions for Innovation
---- PROCESSING PARAMETERS ----
sexp(3.0[Hz], 0.0[s])
trapezoid(0[%], 0[%], 80[%], 100[%])
zerofill(2)
fft(1, TRUE, TRUE)
machinephase
ppm
Filename = GDS-422_Carbon-1-1
Author = delta
Experiment = carbon.jxp
Sample_Id = DSR_GDS-422
Solvent = CHLOROFORM-D
Creation_Time = 8-AUG-2018 19:59:
Revision_Time = 8-FEB-2019 14:40:
Current_Time = 21-FEB-2019 12:41:
Comment = single pulse decou
Data_Format = 1D COMPLEX
Dim_Size = 26214
Dim_Title = Carbon13
Dim_Units = [ppm]
Dimensions = X
Spectrometer = JNM-ECZ400S/L1
Field_Strength = 9.38906137[T] (400
X_Acq_Duration = 0.51904512[s]
X_Domain = Carbon13
X_Freq = 100.51775967[MHz]
X_Offset = 100[ppm]
X_Points = 16384
X_Prescans = 4
X_Resolution = 1.92661478[Hz]
X_Sweep = 31.56565657[kHz]
X_Sweep_Clipped = 25.25252525[kHz]
Irr_Domain = Proton
Irr_Freq = 399.75219779[MHz]
Irr_Offset = 5[ppm]
Blanking = 5[us]
Clipped = FALSE
Decimation_Reg = r: 198(197),g: 39
Scans = 512
Total_Scans = 512
Relaxation_Delay = 2[s]
Recvr_Gain = 56
Temp_Get = 22.1[dC]
X_90_Width = 8.57[us]
X_Acq_Time = 0.51904512[s]
X_Angle = 30[deg]
X_Atn = 7.8[dB]
X_Pulse = 2.85666667[us]
Irr_Atn_Dec = 26.16[dB]
Irr_Atn_Dec_Calc = 26.16[dB]
Irr_Atn_Dec_Default_Calc = 26.16[dB]
Irr_Atn_Noe = 26.16[dB]
(thousandths)
24.0
22.0
20.0
18.0
16.0
14.0
12.0
10.0
8.0
6.0
4.0
2.0
0
150.0
140.0
130.0
120.0
110.0
100.0
90.0
80.0
70.0
60.0
50.0
40.0
30.0
20.0
10.0
0
149.431
139.503
138.410
132.929
129.220
125.866
107.418
77.431
77.115
76.798
13.557
12.484
X : parts per Million : Carbon13

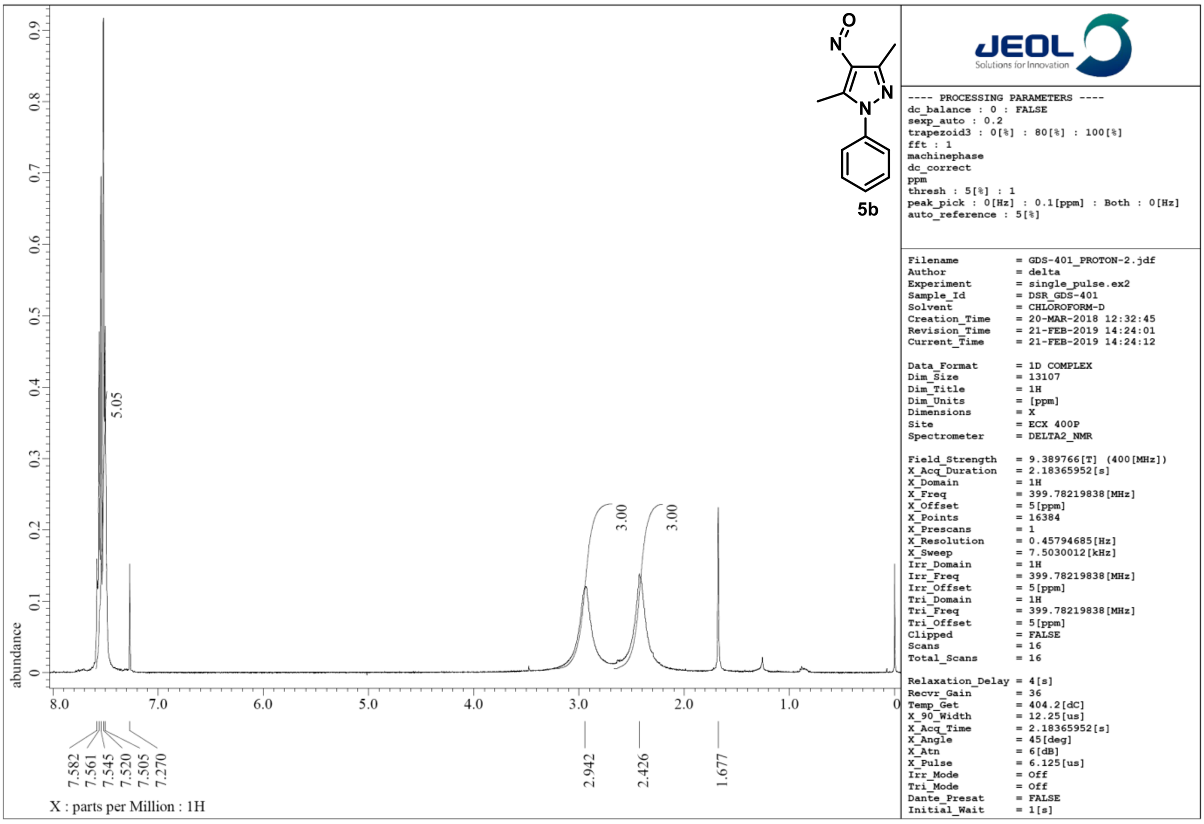
5b
JEOL
Solutions for Innovation
---- PROCESSING PARAMETERS ----
dc_balance : 0 : FALSE
sexp_auto : 0.2
trapezoid3 : 0[%] : 80[%] : 100[%]
fft : 1
machinephase
dc_correct
ppm
thresh : 5[%] : 1
peak_pick : 0[Hz] : 0.1[ppm] : Both : 0[Hz]
auto_reference : 5[%]
Filename = GDS-401_PROTON-2.jdf
Author = delta
Experiment = single_pulse.ex2
Sample_Id = DSR_GDS-401
Solvent = CHLOROFORM-D
Creation_Time = 20-MAR-2018 12:32:45
Revision_Time = 21-FEB-2019 14:24:01
Current_Time = 21-FEB-2019 14:24:12
Data_Format = 1D COMPLEX
Dim_Size = 13107
Dim_Title = 1H
Dim_Units = [ppm]
Dimensions = X
Site = ECX 400P
Spectrometer = DELTA2_NMR
Field_Strength = 9.389766[T] (400[MHz])
X_Acq_Duration = 2.18365952[s]
X_Domain = 1H
X_Freq = 399.78219838[MHz]
X_Offset = 5[ppm]
X_Points = 16384
X_Prescans = 1
X_Resolution = 0.45794685[Hz]
X_Sweep = 7.5030012[kHz]
Irr_Domain = 1H
Irr_Freq = 399.78219838[MHz]
Irr_Offset = 5[ppm]
Tri_Domain = 1H
Tri_Freq = 399.78219838[MHz]
Tri_Offset = 5[ppm]
Clipped = FALSE
Scans = 16
Total_Scans = 16
Relaxation_Delay = 4[s]
Recvr_Gain = 36
Temp_Get = 404.2[dC]
X_90_Width = 12.25[us]
X_Acq_Time = 2.18365952[s]
X_Angle = 45[deg]
X_Atn = 6[dB]
X_Pulse = 6.125[us]
Irr_Mode = Off
Tri_Mode = Off
Dante_Presat = FALSE
Initial_Wait = 1[s]
abundance
5.05
3.00
3.00
7.582
7.561
7.545
7.520
7.505
7.270
2.942
2.426
1.677
X : parts per Million : 1H

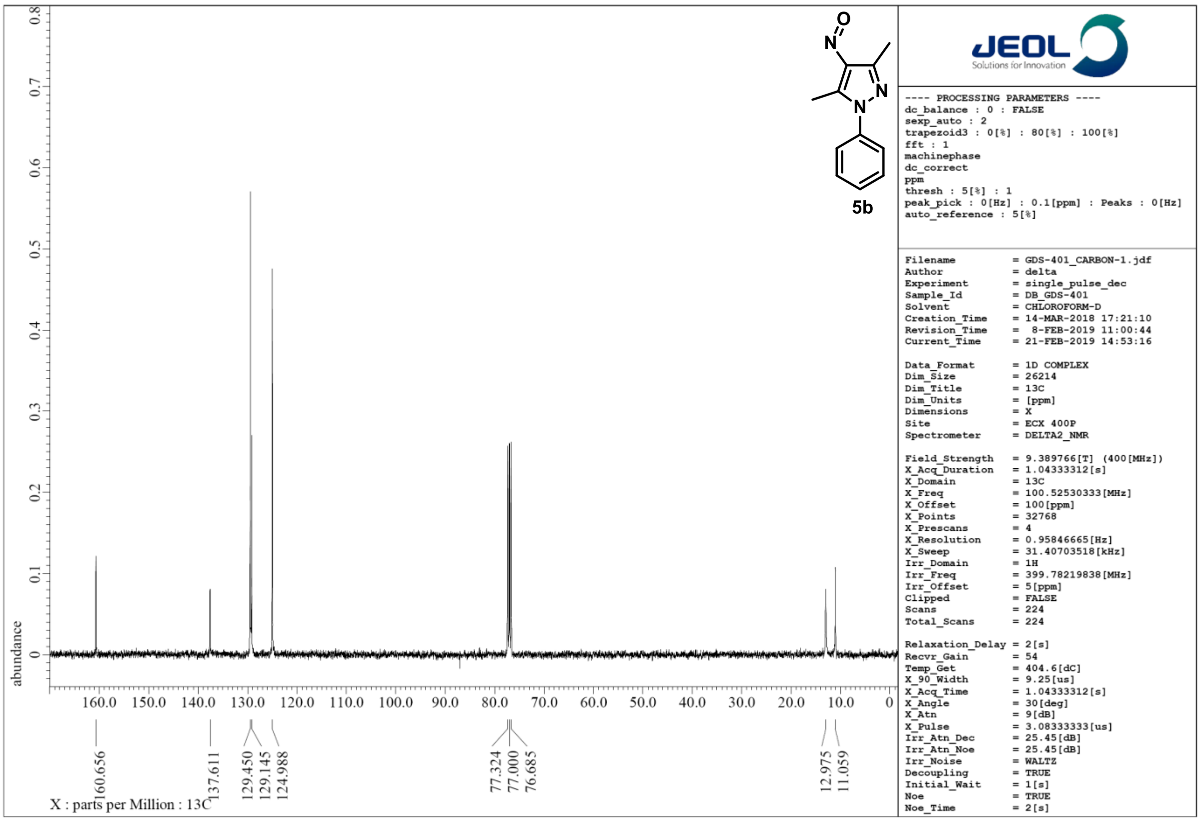
5b
JEOL
Solutions for Innovation
---- PROCESSING PARAMETERS ----
dc_balance : 0 : FALSE
sexp_auto : 2
trapezoid3 : 0[%] : 80[%] : 100[%]
fft : 1
machinephase
dc_correct
ppm
thresh : 5[%] : 1
peak_pick : 0[Hz] : 0.1[ppm] : Peaks : 0[Hz]
auto_reference : 5[%]
Filename = GDS-401_CARBON-1.jdf
Author = delta
Experiment = single_pulse_dec
Sample_Id = DB_GDS-401
Solvent = CHLOROFORM-D
Creation_Time = 14-MAR-2018 17:21:10
Revision_Time = 8-FEB-2019 11:00:44
Current_Time = 21-FEB-2019 14:53:16
Data_Format = 1D COMPLEX
Dim_Size = 26214
Dim_Title = 13C
Dim_Units = [ppm]
Dimensions = X
Site = ECX 400P
Spectrometer = DELTA2_NMR
Field_Strength = 9.389766[T] (400[MHz])
X_Acq_Duration = 1.04333312[s]
X_Domain = 13C
X_Freq = 100.52530333[MHz]
X_Offset = 100[ppm]
X_Points = 32768
X_Prescans = 4
X_Resolution = 0.95846665[Hz]
X_Sweep = 31.40703518[kHz]
Irr_Domain = 1H
Irr_Freq = 399.78219838[MHz]
Irr_Offset = 5[ppm]
Clipped = FALSE
Scans = 224
Total_Scans = 224
Relaxation_Delay = 2[s]
Recvr_Gain = 54
Temp_Get = 404.6[dC]
X_90_Width = 9.25[us]
X_Acq_Time = 1.04333312[s]
X_Angle = 30[deg]
X_Atn = 9[dB]
X_Pulse = 3.08333333[us]
Irr_Atn_Dec = 25.45[dB]
Irr_Atn_Noe = 25.45[dB]
Irr_Noise = WALTZ
Decoupling = TRUE
Initial_Wait = 1[s]
Noe = TRUE
Noe_Time = 2[s]
abundance
160.656
137.611
129.450
129.145
124.988
77.324
77.000
76.685
12.975
11.059
X : parts per Million : 13C

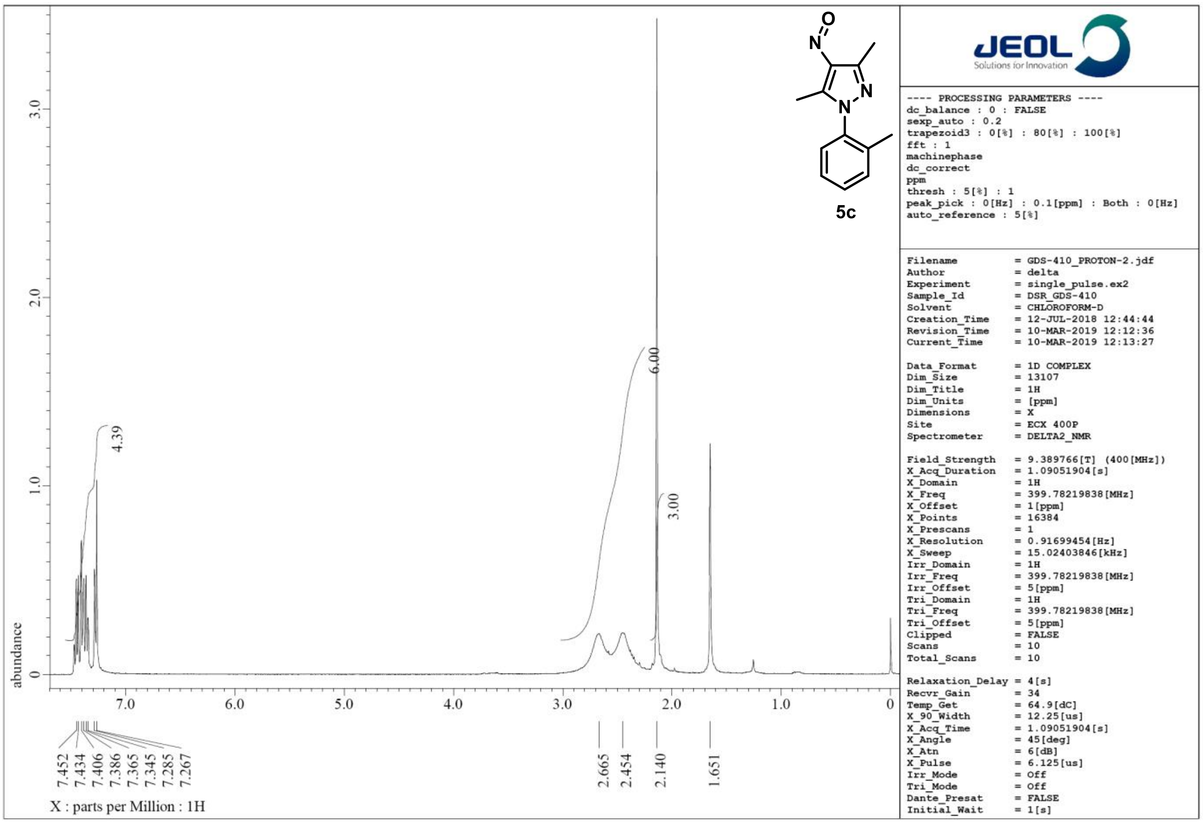
5c
JEOL
Solutions for Innovation
---- PROCESSING PARAMETERS ----
dc_balance : 0 : FALSE
sexp_auto : 0.2
trapezoid3 : 0[%] : 80[%] : 100[%]
fft : 1
machinephase
dc_correct
ppm
thresh : 5[%] : 1
peak_pick : 0[Hz] : 0.1[ppm] : Both : 0[Hz]
auto_reference : 5[%]
Filename = GDS-410_PROTON-2.jdf
Author = delta
Experiment = single_pulse.ex2
Sample_Id = DSR_GDS-410
Solvent = CHLOROFORM-D
Creation_Time = 12-JUL-2018 12:44:44
Revision_Time = 10-MAR-2019 12:12:36
Current_Time = 10-MAR-2019 12:13:27
Data_Format = 1D COMPLEX
Dim_Size = 13107
Dim_Title = 1H
Dim_Units = [ppm]
Dimensions = X
Site = ECX 400P
Spectrometer = DELTA2_NMR
Field_Strength = 9.389766[T] (400[MHz])
X_Acq_Duration = 1.09051904[s]
X_Domain = 1H
X_Freq = 399.78219838[MHz]
X_Offset = 1[ppm]
X_Points = 16384
X_Prescans = 1
X_Resolution = 0.91699454[Hz]
X_Sweep = 15.02403846[kHz]
Irr_Domain = 1H
Irr_Freq = 399.78219838[MHz]
Irr_Offset = 5[ppm]
Tri_Domain = 1H
Tri_Freq = 399.78219838[MHz]
Tri_Offset = 5[ppm]
Clipped = FALSE
Scans = 10
Total_Scans = 10
Relaxation_Delay = 4[s]
Recvr_Gain = 34
Temp_Get = 64.9[dC]
X_90_Width = 12.25[us]
X_Acq_Time = 1.09051904[s]
X_Angle = 45[deg]
X_Atn = 6[dB]
X_Pulse = 6.125[us]
Irr_Mode = Off
Tri_Mode = Off
Dante_Presat = FALSE
Initial_Wait = 1[s]
abundance
3.0
2.0
1.0
0
4.39
6.00
3.00
7.0
6.0
5.0
4.0
3.0
2.0
1.0
0
7.452
7.434
7.406
7.386
7.365
7.345
7.285
7.267
2.665
2.454
2.140
1.651
X : parts per Million : 1H

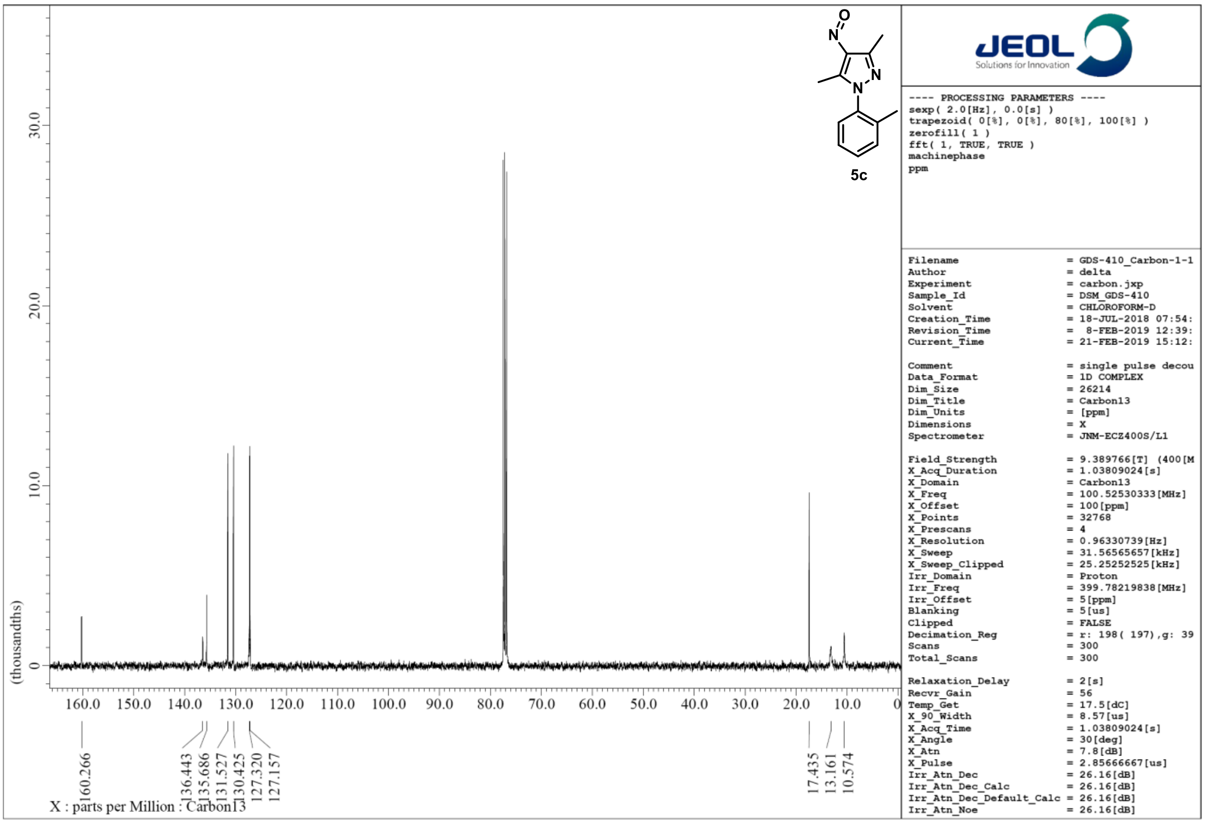
5c
JEOL
Solutions for Innovation
---- PROCESSING PARAMETERS ----
sexp(2.0[Hz], 0.0[s])
trapezoid(0[%], 0[%], 80[%], 100[%])
zerofill(1)
fft(1, TRUE, TRUE)
machinephase
ppm
Filename = GDS-410_Carbon-1-1
Author = delta
Experiment = carbon.jxp
Sample_Id = DSM_GDS-410
Solvent = CHLOROFORM-D
Creation_Time = 18-JUL-2018 07:54:
Revision_Time = 8-FEB-2019 12:39:
Current_Time = 21-FEB-2019 15:12:
Comment = single pulse decou
Data_Format = 1D COMPLEX
Dim_Size = 26214
Dim_Title = Carbon13
Dim_Units = [ppm]
Dimensions = X
Spectrometer = JNM-ECZ400S/L1
Field_Strength = 9.389766[T] (400[M
X_Acq_Duration = 1.03809024[s]
X_Domain = Carbon13
X_Freq = 100.52530333[MHz]
X_Offset = 100[ppm]
X_Points = 32768
X_Prescans = 4
X_Resolution = 0.96330739[Hz]
X_Sweep = 31.56565657[kHz]
X_Sweep_Clipped = 25.25252525[kHz]
Irr_Domain = Proton
Irr_Freq = 399.78219838[MHz]
Irr_Offset = 5[ppm]
Blanking = 5[us]
Clipped = FALSE
Decimation_Reg = r: 198(197),g: 39
Scans = 300
Total_Scans = 300
Relaxation_Delay = 2[s]
Recvr_Gain = 56
Temp_Get = 17.5[dC]
X_90_Width = 8.57[us]
X_Acq_Time = 1.03809024[s]
X_Angle = 30[deg]
X_Atn = 7.8[dB]
X_Pulse = 2.85666667[us]
Irr_Atn_Dec = 26.16[dB]
Irr_Atn_Dec_Calc = 26.16[dB]
Irr_Atn_Dec_Default_Calc = 26.16[dB]
Irr_Atn_Noe = 26.16[dB]
(thousandths)
30.0
20.0
10.0
0
160.0
150.0
140.0
130.0
120.0
110.0
100.0
90.0
80.0
70.0
60.0
50.0
40.0
30.0
20.0
10.0
0
160.266
136.443
135.686
131.527
130.425
127.320
127.157
17.435
13.161
10.574
X : parts per Million : Carbon13

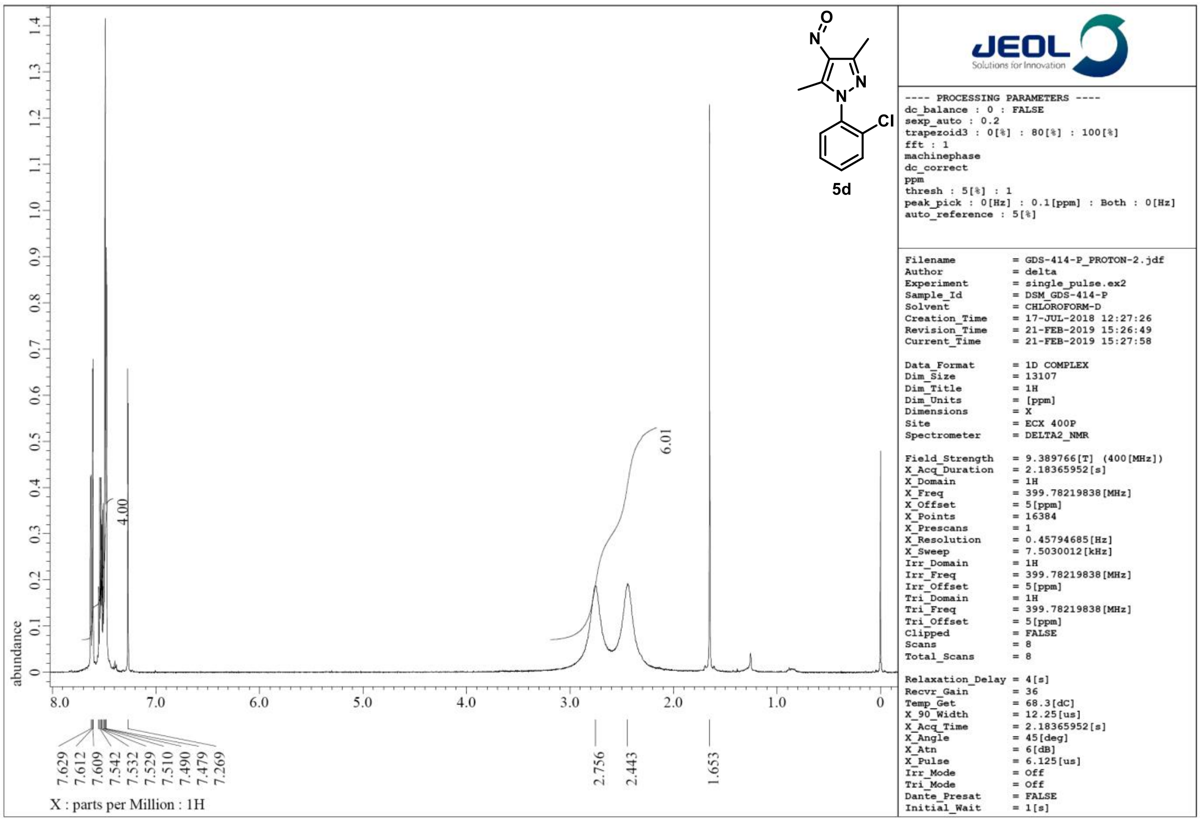

5d
Cl
N
N
N
O
abundance
X : parts per Million : 1H
4.00
6.01
7.629
7.612
7.609
7.542
7.532
7.529
7.510
7.490
7.479
7.269
2.756
2.443
1.653
---- PROCESSING PARAMETERS ----
dc_balance : 0 : FALSE
sexp_auto : 0.2
trapezoid3 : 0[%] : 80[%] : 100[%]
fft : 1
machinephase
dc_correct
ppm
thresh : 5[%] : 1
peak_pick : 0[Hz] : 0.1[ppm] : Both : 0[Hz]
auto_reference : 5[%]
Filename = GDS-414-P_PROTON-2.jdf
Author = delta
Experiment = single_pulse.ex2
Sample_Id = DSM_GDS-414-P
Solvent = CHLOROFORM-D
Creation_Time = 17-JUL-2018 12:27:26
Revision_Time = 21-FEB-2019 15:26:49
Current_Time = 21-FEB-2019 15:27:58
Data_Format = 1D COMPLEX
Dim_Size = 13107
Dim_Title = 1H
Dim_Units = [ppm]
Dimensions = X
Site = ECX 400P
Spectrometer = DELTA2_NMR
Field_Strength = 9.389766[T] (400[MHz])
X_Acq_Duration = 2.18365952[s]
X_Domain = 1H
X_Freq = 399.78219838[MHz]
X_Offset = 5[ppm]
X_Points = 16384
X_Prescans = 1
X_Resolution = 0.45794685[Hz]
X_Sweep = 7.5030012[kHz]
Irr_Domain = 1H
Irr_Freq = 399.78219838[MHz]
Irr_Offset = 5[ppm]
Tri_Domain = 1H
Tri_Freq = 399.78219838[MHz]
Tri_Offset = 5[ppm]
Clipped = FALSE
Scans = 8
Total_Scans = 8
Relaxation_Delay = 4[s]
Recvr_Gain = 36
Temp_Get = 68.3[dC]
X_90_Width = 12.25[us]
X_Acq_Time = 2.18365952[s]
X_Angle = 45[deg]
X_Atn = 6[dB]
X_Pulse = 6.125[us]
Irr_Mode = Off
Tri_Mode = Off
Dante_Presat = FALSE
Initial_Wait = 1[s]

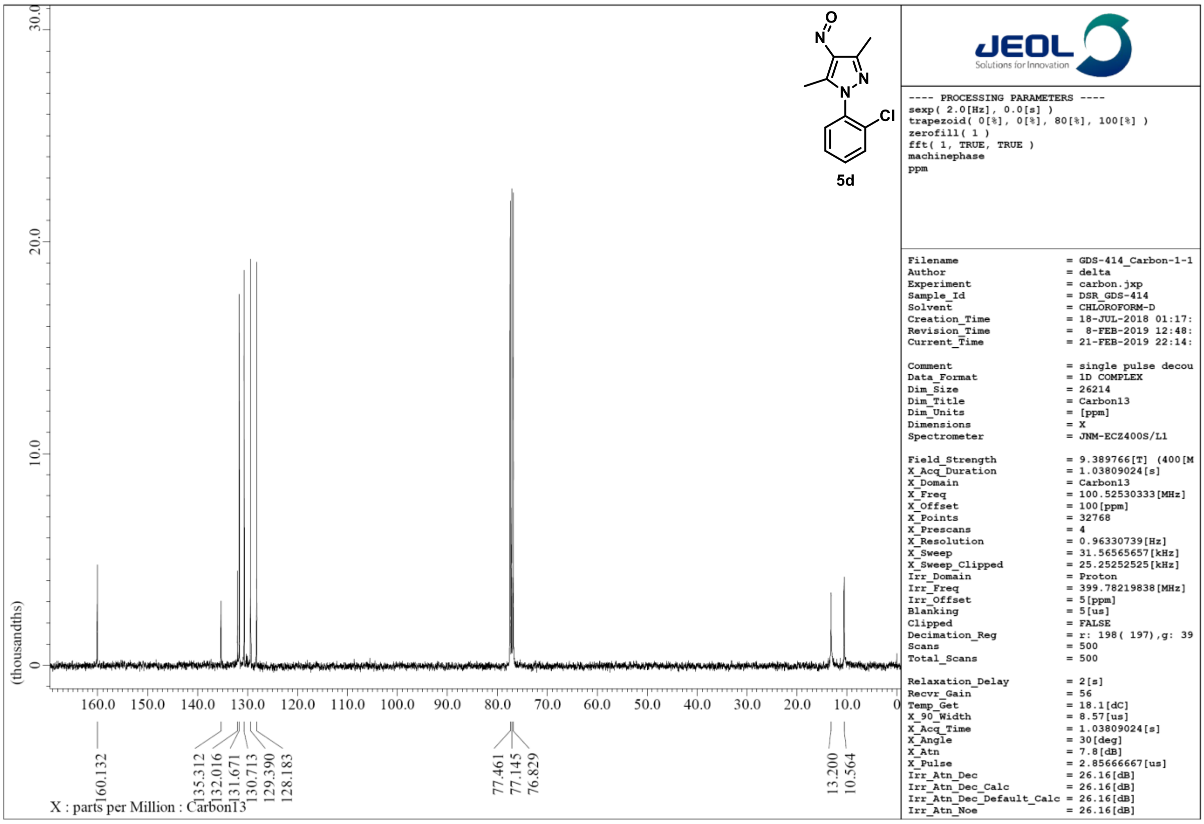

5d
Cl
N
N
N
O
(thousandths)
X : parts per Million : Carbon13
160.132
135.312
132.016
131.671
130.713
129.390
128.183
77.461
77.145
76.829
13.200
10.564
---- PROCESSING PARAMETERS ----
sexp(2.0[Hz], 0.0[s])
trapezoid(0[%], 0[%], 80[%], 100[%])
zerofill(1)
fft(1, TRUE, TRUE)
machinephase
ppm
Filename = GDS-414_Carbon-1-1
Author = delta
Experiment = carbon.jxp
Sample_Id = DSR_GDS-414
Solvent = CHLOROFORM-D
Creation_Time = 18-JUL-2018 01:17:
Revision_Time = 8-FEB-2019 12:48:
Current_Time = 21-FEB-2019 22:14:
Comment = single pulse decou
Data_Format = 1D COMPLEX
Dim_Size = 26214
Dim_Title = Carbon13
Dim_Units = [ppm]
Dimensions = X
Spectrometer = JNM-ECZ400S/L1
Field_Strength = 9.389766[T] (400[M
X_Acq_Duration = 1.03809024[s]
X_Domain = Carbon13
X_Freq = 100.52530333[MHz]
X_Offset = 100[ppm]
X_Points = 32768
X_Prescans = 4
X_Resolution = 0.96330739[Hz]
X_Sweep = 31.56565657[kHz]
X_Sweep_Clipped = 25.25252525[kHz]
Irr_Domain = Proton
Irr_Freq = 399.78219838[MHz]
Irr_Offset = 5[ppm]
Blanking = 5[us]
Clipped = FALSE
Decimation_Reg = r: 198(197),g: 39
Scans = 500
Total_Scans = 500
Relaxation_Delay = 2[s]
Recvr_Gain = 56
Temp_Get = 18.1[dC]
X_90_Width = 8.57[us]
X_Acq_Time = 1.03809024[s]
X_Angle = 30[deg]
X_Atn = 7.8[dB]
X_Pulse = 2.85666667[us]
Irr_Atn_Dec = 26.16[dB]
Irr_Atn_Dec_Calc = 26.16[dB]
Irr_Atn_Dec_Default_Calc = 26.16[dB]
Irr_Atn_Noe = 26.16[dB]

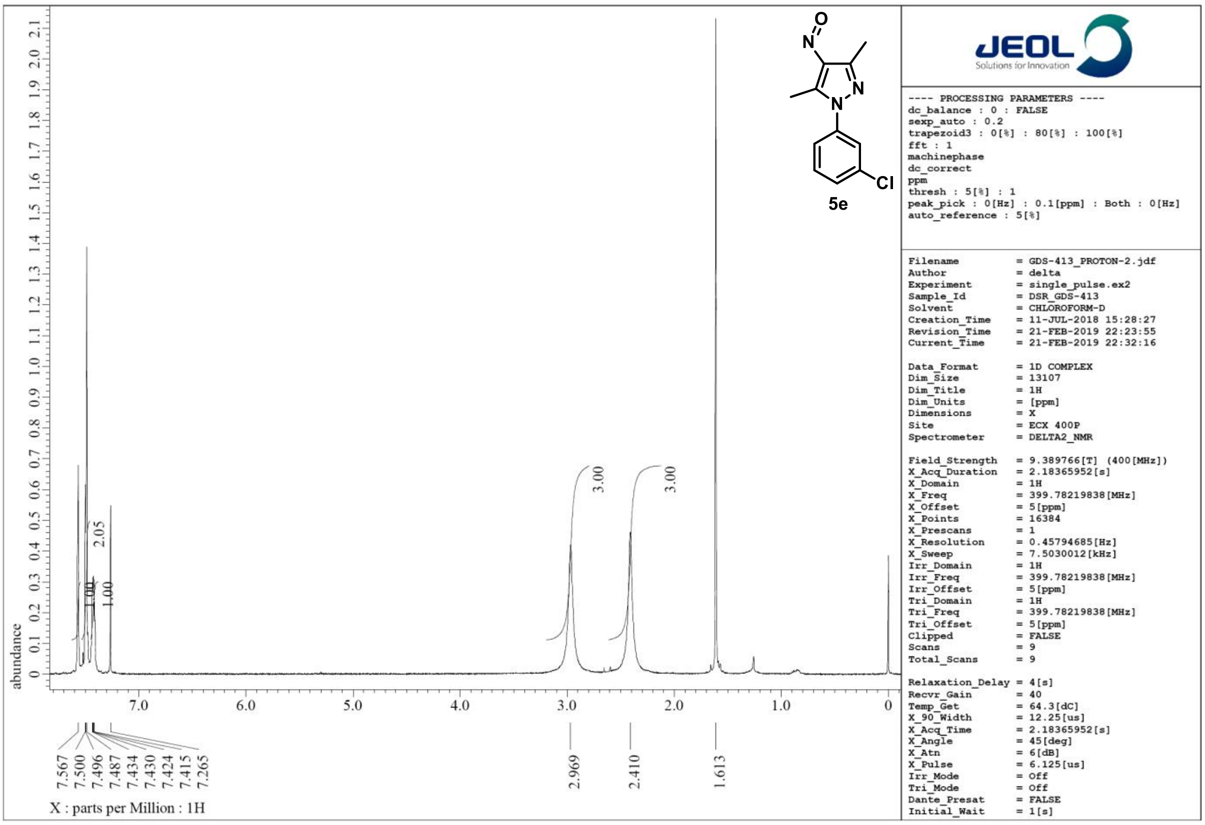
5e
JEOL
Solutions for Innovation
---- PROCESSING PARAMETERS ----
dc_balance : 0 : FALSE
sexp_auto : 0.2
trapezoid3 : 0[%] : 80[%] : 100[%]
fft : 1
machinephase
dc_correct
ppm
thresh : 5[%] : 1
peak_pick : 0[Hz] : 0.1[ppm] : Both : 0[Hz]
auto_reference : 5[%]
Filename = GDS-413_PROTON-2.jdf
Author = delta
Experiment = single_pulse.ex2
Sample_Id = DSR_GDS-413
Solvent = CHLOROFORM-D
Creation_Time = 11-JUL-2018 15:28:27
Revision_Time = 21-FEB-2019 22:23:55
Current_Time = 21-FEB-2019 22:32:16
Data_Format = 1D COMPLEX
Dim_Size = 13107
Dim_Title = 1H
Dim_Units = [ppm]
Dimensions = X
Site = ECX 400P
Spectrometer = DELTA2_NMR
Field_Strength = 9.389766[T] (400[MHz])
X_Acq_Duration = 2.18365952[s]
X_Domain = 1H
X_Freq = 399.78219838[MHz]
X_Offset = 5[ppm]
X_Points = 16384
X_Prescans = 1
X_Resolution = 0.45794685[Hz]
X_Sweep = 7.5030012[kHz]
Irr_Domain = 1H
Irr_Freq = 399.78219838[MHz]
Irr_Offset = 5[ppm]
Tri_Domain = 1H
Tri_Freq = 399.78219838[MHz]
Tri_Offset = 5[ppm]
Clipped = FALSE
Scans = 9
Total_Scans = 9
Relaxation_Delay = 4[s]
Recvr_Gain = 40
Temp_Get = 64.3[dC]
X_90_Width = 12.25[us]
X_Acq_Time = 2.18365952[s]
X_Angle = 45[deg]
X_Atn = 6[dB]
X_Pulse = 6.125[us]
Irr_Mode = Off
Tri_Mode = Off
Dante_Presat = FALSE
Initial_Wait = 1[s]
abundance
3.00
3.00
2.05
1.00
1.00
7.567
7.500
7.496
7.487
7.434
7.430
7.424
7.415
7.265
2.969
2.410
1.613
X : parts per Million : 1H

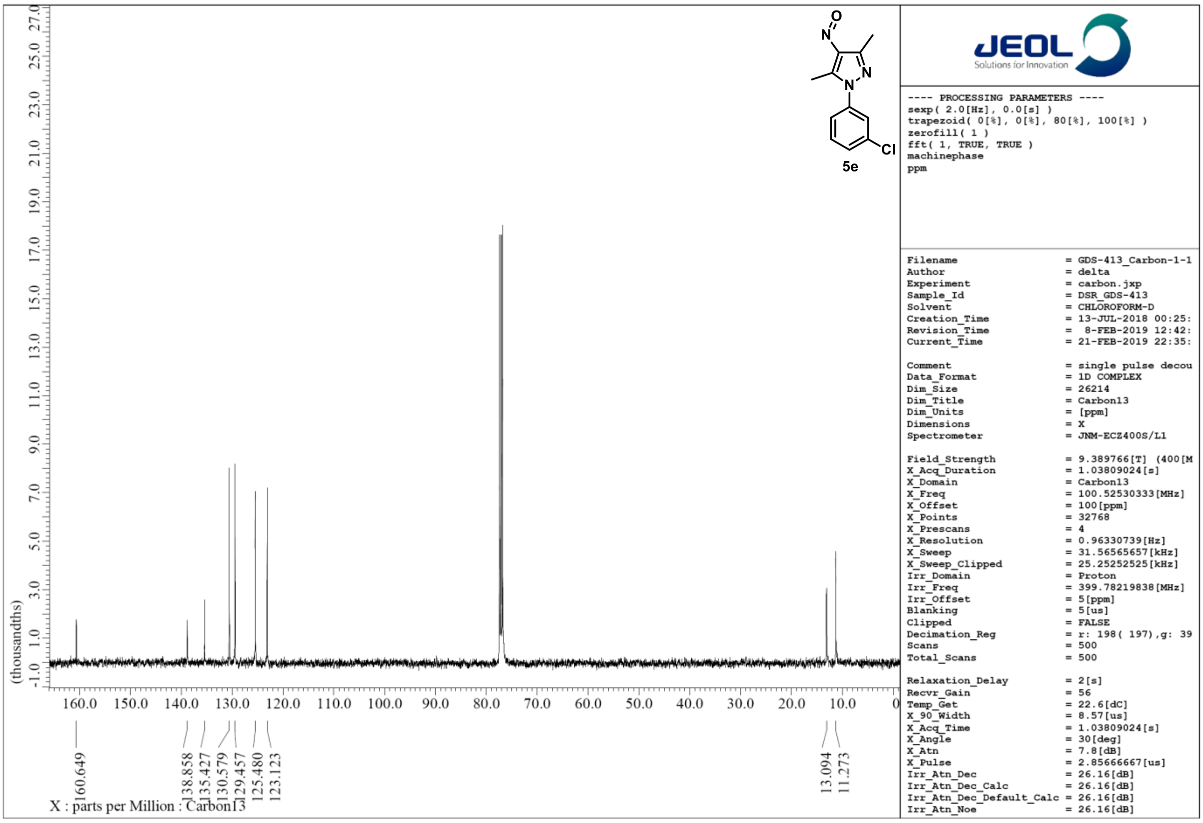
5e
JEOL
Solutions for Innovation
---- PROCESSING PARAMETERS ----
sexp(2.0[Hz], 0.0[s])
trapezoid(0[%], 0[%], 80[%], 100[%])
zerofill(1)
fft(1, TRUE, TRUE)
machinephase
ppm
Filename = GDS-413_Carbon-1-1
Author = delta
Experiment = carbon.jxp
Sample_Id = DSR_GDS-413
Solvent = CHLOROFORM-D
Creation_Time = 13-JUL-2018 00:25:
Revision_Time = 8-FEB-2019 12:42:
Current_Time = 21-FEB-2019 22:35:
Comment = single pulse decou
Data_Format = 1D COMPLEX
Dim_Size = 26214
Dim_Title = Carbon13
Dim_Units = [ppm]
Dimensions = X
Spectrometer = JNM-ECZ400S/L1
Field_Strength = 9.389766[T] (400[M
X_Acq_Duration = 1.03809024[s]
X_Domain = Carbon13
X_Freq = 100.52530333[MHz]
X_Offset = 100[ppm]
X_Points = 32768
X_Prescans = 4
X_Resolution = 0.96330739[Hz]
X_Sweep = 31.56565657[kHz]
X_Sweep_Clipped = 25.25252525[kHz]
Irr_Domain = Proton
Irr_Freq = 399.78219838[MHz]
Irr_Offset = 5[ppm]
Blanking = 5[us]
Clipped = FALSE
Decimation_Reg = r: 198(197),g: 39
Scans = 500
Total_Scans = 500
Relaxation_Delay = 2[s]
Recvr_Gain = 56
Temp_Get = 22.6[dC]
X_90_Width = 8.57[us]
X_Acq_Time = 1.03809024[s]
X_Angle = 30[deg]
X_Atn = 7.8[dB]
X_Pulse = 2.85666667[us]
Irr_Atn_Dec = 26.16[dB]
Irr_Atn_Dec_Calc = 26.16[dB]
Irr_Atn_Dec_Default_Calc = 26.16[dB]
Irr_Atn_Noe = 26.16[dB]
(thousandths)
160.649
138.858
135.427
130.579
129.457
125.480
123.123
13.094
11.273
X : parts per Million : Carbon13

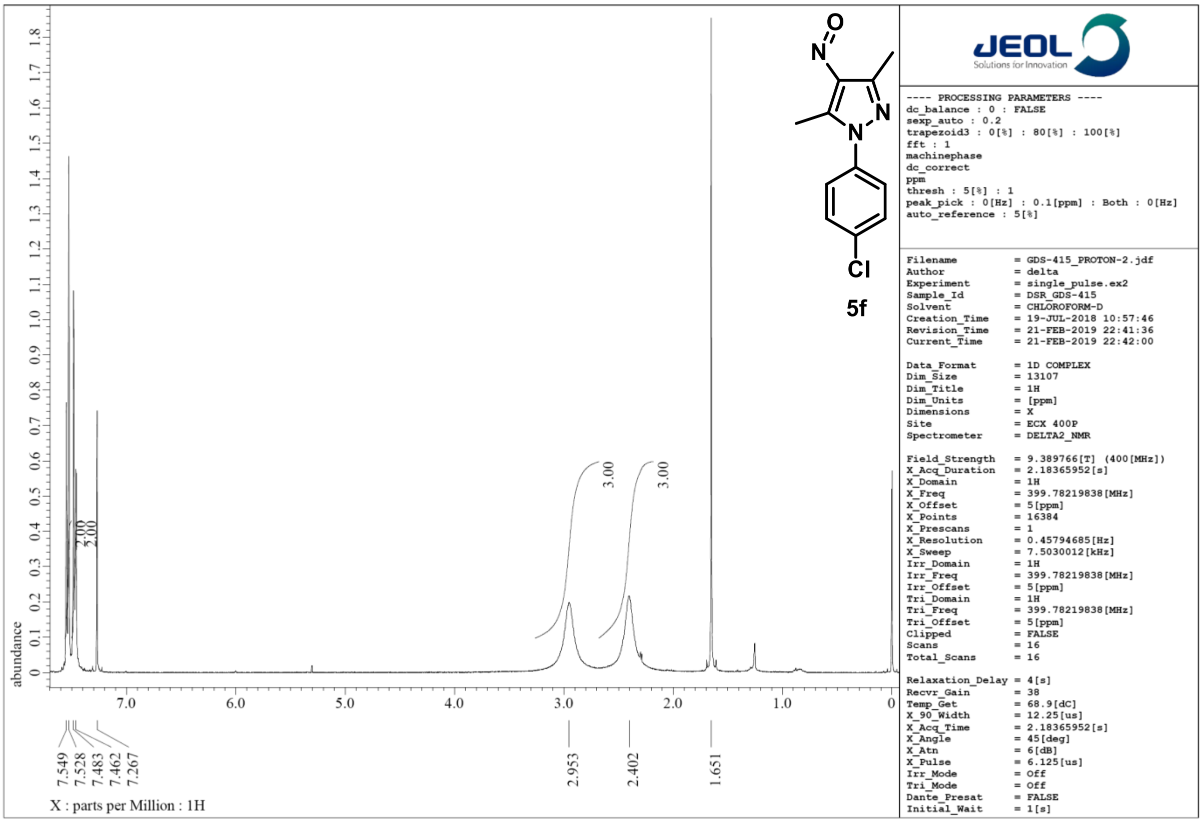
5f
JEOL
Solutions for Innovation
---- PROCESSING PARAMETERS ----
dc_balance : 0 : FALSE
sexp_auto : 0.2
trapezoid3 : 0[%] : 80[%] : 100[%]
fft : 1
machinephase
dc_correct
ppm
thresh : 5[%] : 1
peak_pick : 0[Hz] : 0.1[ppm] : Both : 0[Hz]
auto_reference : 5[%]
Filename = GDS-415_PROTON-2.jdf
Author = delta
Experiment = single_pulse.ex2
Sample_Id = DSR_GDS-415
Solvent = CHLOROFORM-D
Creation_Time = 19-JUL-2018 10:57:46
Revision_Time = 21-FEB-2019 22:41:36
Current_Time = 21-FEB-2019 22:42:00
Data_Format = 1D COMPLEX
Dim_Size = 13107
Dim_Title = 1H
Dim_Units = [ppm]
Dimensions = X
Site = ECX 400P
Spectrometer = DELTA2_NMR
Field_Strength = 9.389766[T] (400[MHz])
X_Acq_Duration = 2.18365952[s]
X_Domain = 1H
X_Freq = 399.78219838[MHz]
X_Offset = 5[ppm]
X_Points = 16384
X_Prescans = 1
X_Resolution = 0.45794685[Hz]
X_Sweep = 7.5030012[kHz]
Irr_Domain = 1H
Irr_Freq = 399.78219838[MHz]
Irr_Offset = 5[ppm]
Tri_Domain = 1H
Tri_Freq = 399.78219838[MHz]
Tri_Offset = 5[ppm]
Clipped = FALSE
Scans = 16
Total_Scans = 16
Relaxation_Delay = 4[s]
Recvr_Gain = 38
Temp_Get = 68.9[dC]
X_90_Width = 12.25[us]
X_Acq_Time = 2.18365952[s]
X_Angle = 45[deg]
X_Atn = 6[dB]
X_Pulse = 6.125[us]
Irr_Mode = Off
Tri_Mode = Off
Dante_Presat = FALSE
Initial_Wait = 1[s]
abundance
3.00
3.00
2.00
2.00
7.549
7.528
7.483
7.462
7.267
2.953
2.402
1.651
X : parts per Million : 1H

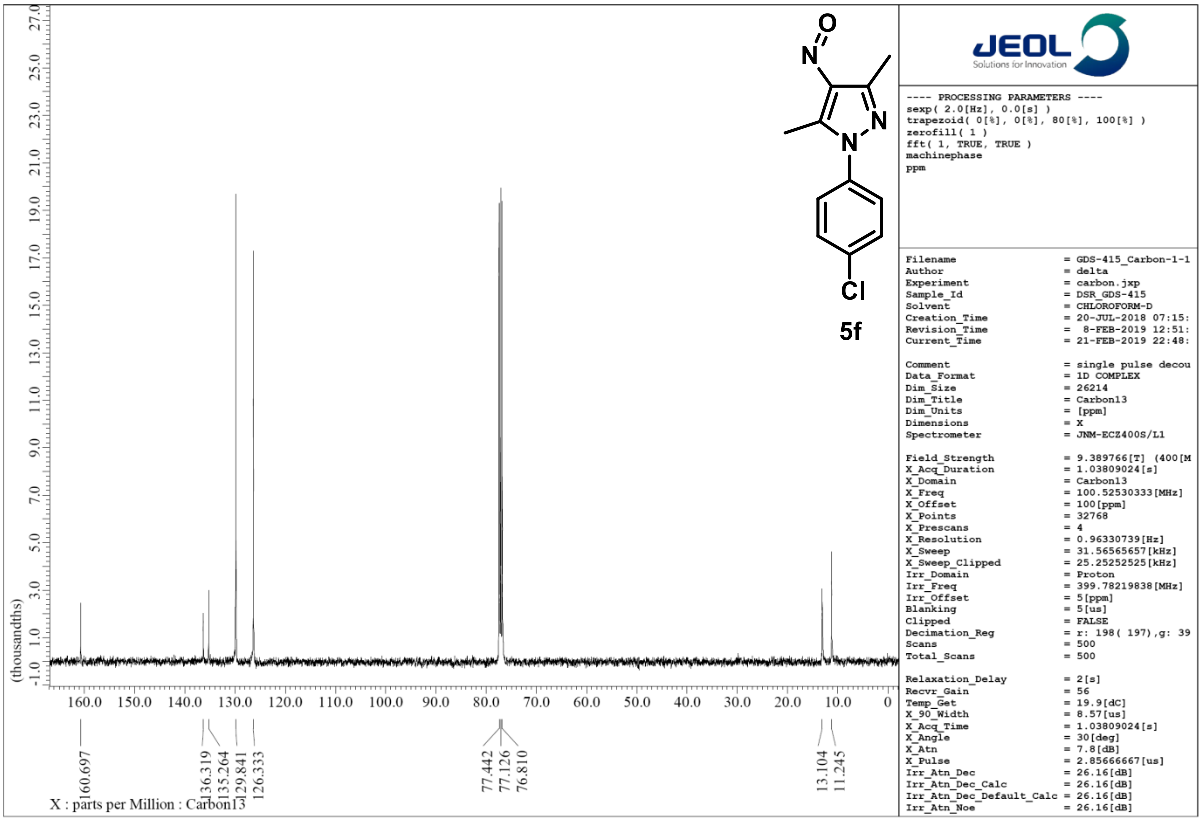
5f
JEOL
Solutions for Innovation
---- PROCESSING PARAMETERS ----
sexp(2.0[Hz], 0.0[s])
trapezoid(0[%], 0[%], 80[%], 100[%])
zerofill(1)
fft(1, TRUE, TRUE)
machinephase
ppm
Filename = GDS-415_Carbon-1-1
Author = delta
Experiment = carbon.jxp
Sample_Id = DSR_GDS-415
Solvent = CHLOROFORM-D
Creation_Time = 20-JUL-2018 07:15:
Revision_Time = 8-FEB-2019 12:51:
Current_Time = 21-FEB-2019 22:48:
Comment = single pulse decou
Data_Format = 1D COMPLEX
Dim_Size = 26214
Dim_Title = Carbon13
Dim_Units = [ppm]
Dimensions = X
Spectrometer = JNM-ECZ400S/L1
Field_Strength = 9.389766[T] (400[M
X_Acq_Duration = 1.03809024[s]
X_Domain = Carbon13
X_Freq = 100.52530333[MHz]
X_Offset = 100[ppm]
X_Points = 32768
X_Prescans = 4
X_Resolution = 0.96330739[Hz]
X_Sweep = 31.56565657[kHz]
X_Sweep_Clipped = 25.25252525[kHz]
Irr_Domain = Proton
Irr_Freq = 399.78219838[MHz]
Irr_Offset = 5[ppm]
Blanking = 5[us]
Clipped = FALSE
Decimation_Reg = r: 198(197),g: 39
Scans = 500
Total_Scans = 500
Relaxation_Delay = 2[s]
Recvr_Gain = 56
Temp_Get = 19.9[dC]
X_90_Width = 8.57[us]
X_Acq_Time = 1.03809024[s]
X_Angle = 30[deg]
X_Atn = 7.8[dB]
X_Pulse = 2.85666667[us]
Irr_Atn_Dec = 26.16[dB]
Irr_Atn_Dec_Calc = 26.16[dB]
Irr_Atn_Dec_Default_Calc = 26.16[dB]
Irr_Atn_Noe = 26.16[dB]
(thousandths)
160.697
136.319
135.264
129.841
126.333
77.442
77.126
76.810
13.104
11.245
X : parts per Million : Carbon13

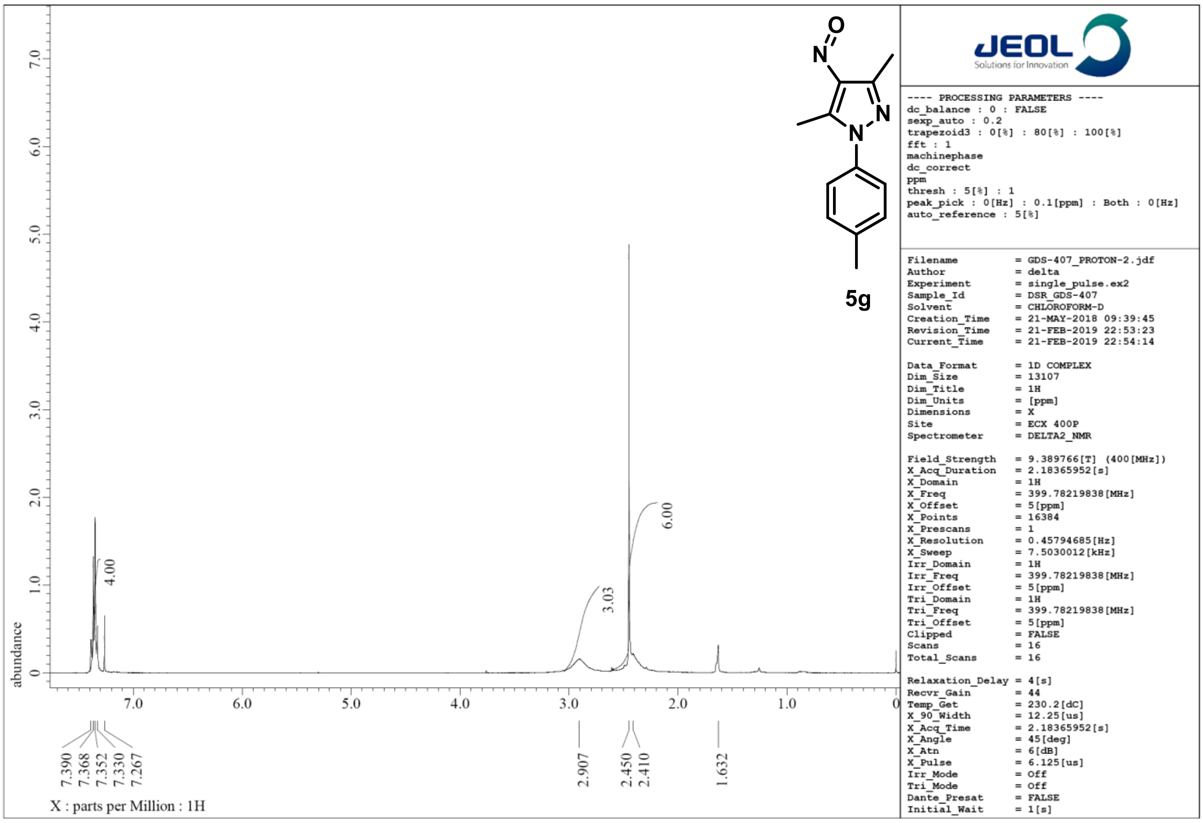
5g
abundance
4.00
3.03
6.00
7.390
7.368
7.352
7.330
7.267
2.907
2.450
2.410
1.632
X : parts per Million : 1H
JEOL
Solutions for Innovation
---- PROCESSING PARAMETERS ----
dc_balance : 0 : FALSE
sexp_auto : 0.2
trapezoid3 : 0[%] : 80[%] : 100[%]
fft : 1
machinephase
dc_correct
ppm
thresh : 5[%] : 1
peak_pick : 0[Hz] : 0.1[ppm] : Both : 0[Hz]
auto_reference : 5[%]
Filename = GDS-407_PROTON-2.jdf
Author = delta
Experiment = single_pulse.ex2
Sample_Id = DSR_GDS-407
Solvent = CHLOROFORM-D
Creation_Time = 21-MAY-2018 09:39:45
Revision_Time = 21-FEB-2019 22:53:23
Current_Time = 21-FEB-2019 22:54:14
Data_Format = 1D COMPLEX
Dim_Size = 13107
Dim_Title = 1H
Dim_Units = [ppm]
Dimensions = X
Site = ECX 400P
Spectrometer = DELTA2_NMR
Field_Strength = 9.389766[T] (400[MHz])
X_Acq_Duration = 2.18365952[s]
X_Domain = 1H
X_Freq = 399.78219838[MHz]
X_Offset = 5[ppm]
X_Points = 16384
X_Prescans = 1
X_Resolution = 0.45794685[Hz]
X_Sweep = 7.5030012[kHz]
Irr_Domain = 1H
Irr_Freq = 399.78219838[MHz]
Irr_Offset = 5[ppm]
Tri_Domain = 1H
Tri_Freq = 399.78219838[MHz]
Tri_Offset = 5[ppm]
Clipped = FALSE
Scans = 16
Total_Scans = 16
Relaxation_Delay = 4[s]
Recvr_Gain = 44
Temp_Get = 230.2[dC]
X_90_Width = 12.25[us]
X_Acq_Time = 2.18365952[s]
X_Angle = 45[deg]
X_Atn = 6[dB]
X_Pulse = 6.125[us]
Irr_Mode = Off
Tri_Mode = Off
Dante_Presat = FALSE
Initial_Wait = 1[s]

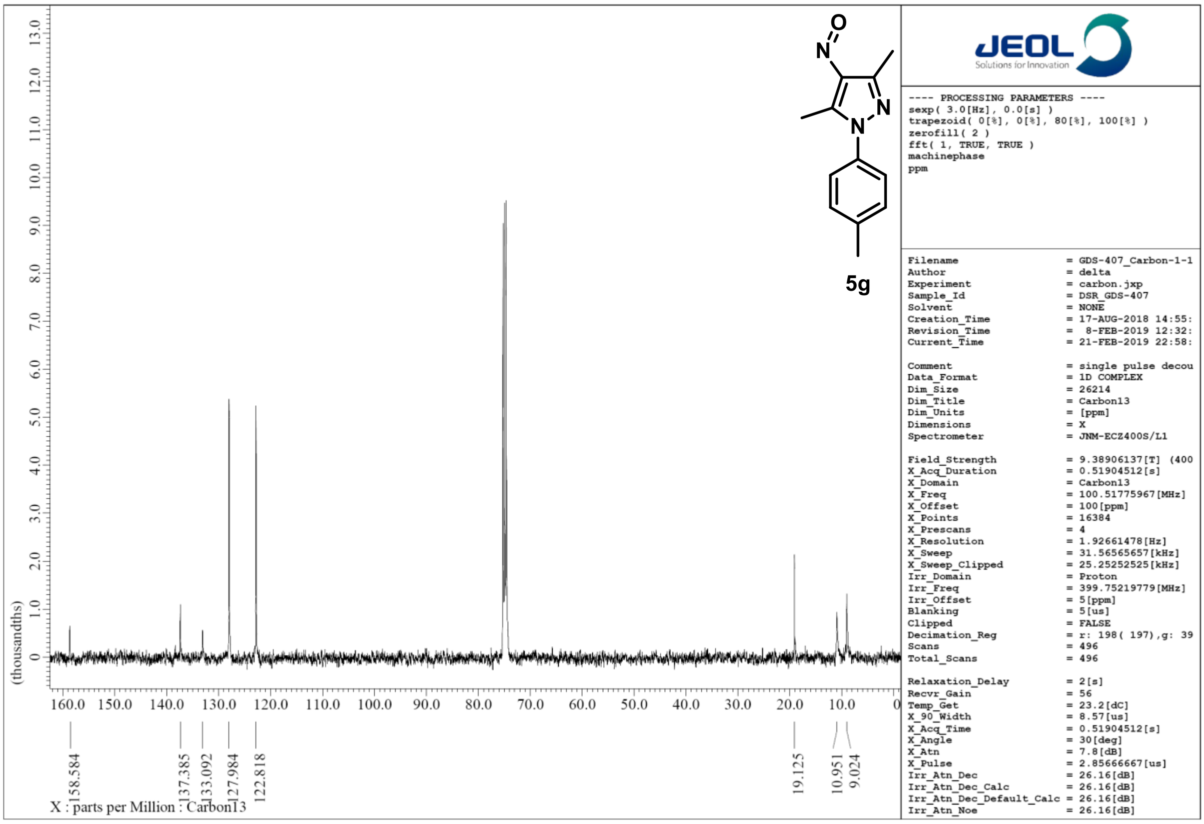
5g
(thousandths)
158.584
137.385
133.092
127.984
122.818
19.125
10.951
9.024
X : parts per Million : Carbon13
JEOL
Solutions for Innovation
---- PROCESSING PARAMETERS ----
sexp(3.0[Hz], 0.0[s])
trapezoid(0[%], 0[%], 80[%], 100[%])
zerofill(2)
fft(1, TRUE, TRUE)
machinephase
ppm
Filename = GDS-407_Carbon-1-1
Author = delta
Experiment = carbon.jxp
Sample_Id = DSR_GDS-407
Solvent = NONE
Creation_Time = 17-AUG-2018 14:55:
Revision_Time = 8-FEB-2019 12:32:
Current_Time = 21-FEB-2019 22:58:
Comment = single pulse decou
Data_Format = 1D COMPLEX
Dim_Size = 26214
Dim_Title = Carbon13
Dim_Units = [ppm]
Dimensions = X
Spectrometer = JNM-ECZ400S/L1
Field_Strength = 9.38906137[T] (400
X_Acq_Duration = 0.51904512[s]
X_Domain = Carbon13
X_Freq = 100.51775967[MHz]
X_Offset = 100[ppm]
X_Points = 16384
X_Prescans = 4
X_Resolution = 1.92661478[Hz]
X_Sweep = 31.56565657[kHz]
X_Sweep_Clipped = 25.25252525[kHz]
Irr_Domain = Proton
Irr_Freq = 399.75219779[MHz]
Irr_Offset = 5[ppm]
Blanking = 5[us]
Clipped = FALSE
Decimation_Reg = r: 198(197),g: 39
Scans = 496
Total_Scans = 496
Relaxation_Delay = 2[s]
Recvr_Gain = 56
Temp_Get = 23.2[dC]
X_90_Width = 8.57[us]
X_Acq_Time = 0.51904512[s]
X_Angle = 30[deg]
X_Atn = 7.8[dB]
X_Pulse = 2.85666667[us]
Irr_Atn_Dec = 26.16[dB]
Irr_Atn_Dec_Calc = 26.16[dB]
Irr_Atn_Dec_Default_Calc = 26.16[dB]
Irr_Atn_Noe = 26.16[dB]

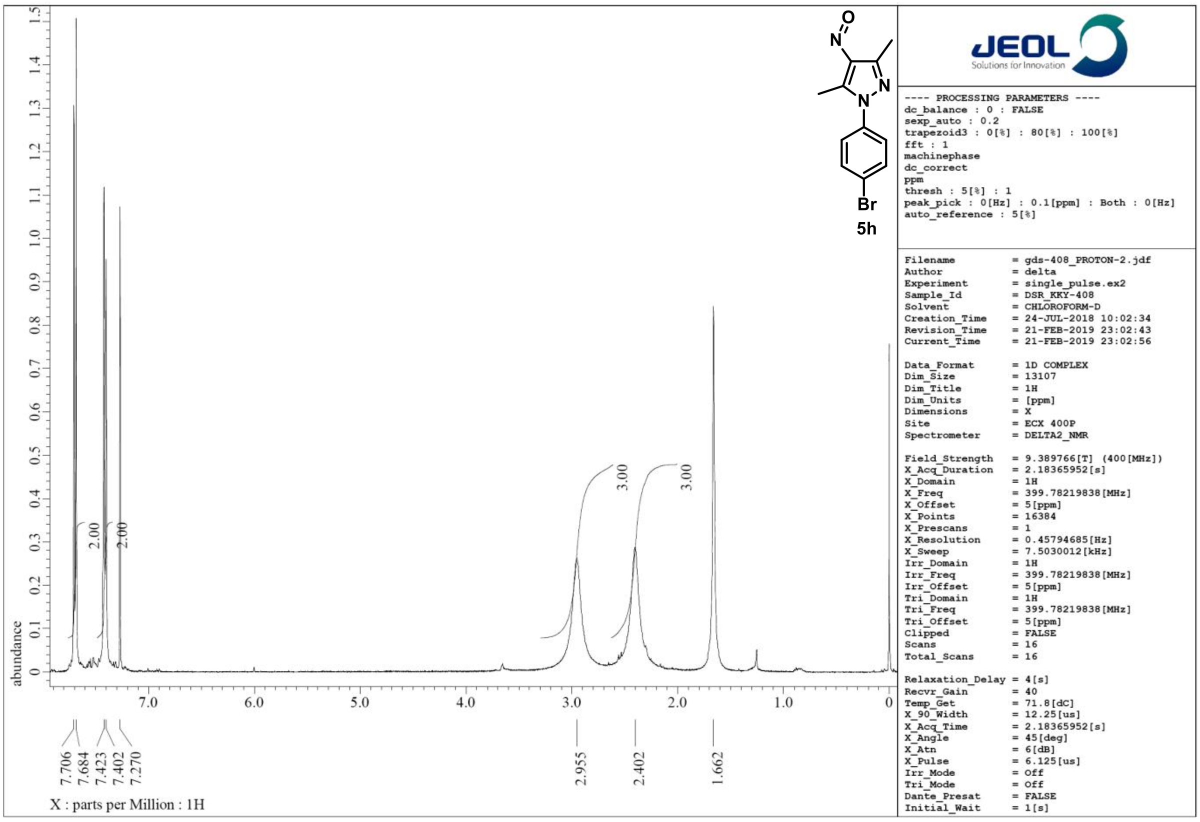
5h
Br
N
N
N
O
JEOL
Solutions for Innovation
---- PROCESSING PARAMETERS ----
dc_balance : 0 : FALSE
sexp_auto : 0.2
trapezoid3 : 0[%] : 80[%] : 100[%]
fft : 1
machinephase
dc_correct
ppm
thresh : 5[%] : 1
peak_pick : 0[Hz] : 0.1[ppm] : Both : 0[Hz]
auto_reference : 5[%]
Filename = gds-408_PROTON-2.jdf
Author = delta
Experiment = single_pulse.ex2
Sample_Id = DSR_KKY-408
Solvent = CHLOROFORM-D
Creation_Time = 24-JUL-2018 10:02:34
Revision_Time = 21-FEB-2019 23:02:43
Current_Time = 21-FEB-2019 23:02:56
Data_Format = 1D COMPLEX
Dim_Size = 13107
Dim_Title = 1H
Dim_Units = [ppm]
Dimensions = X
Site = ECX 400P
Spectrometer = DELTA2_NMR
Field_Strength = 9.389766[T] (400[MHz])
X_Acq_Duration = 2.18365952[s]
X_Domain = 1H
X_Freq = 399.78219838[MHz]
X_Offset = 5[ppm]
X_Points = 16384
X_Prescans = 1
X_Resolution = 0.45794685[Hz]
X_Sweep = 7.5030012[kHz]
Irr_Domain = 1H
Irr_Freq = 399.78219838[MHz]
Irr_Offset = 5[ppm]
Tri_Domain = 1H
Tri_Freq = 399.78219838[MHz]
Tri_Offset = 5[ppm]
Clipped = FALSE
Scans = 16
Total_Scans = 16
Relaxation_Delay = 4[s]
Recvr_Gain = 40
Temp_Get = 71.8[dC]
X_90_Width = 12.25[us]
X_Acq_Time = 2.18365952[s]
X_Angle = 45[deg]
X_Atn = 6[dB]
X_Pulse = 6.125[us]
Irr_Mode = Off
Tri_Mode = Off
Dante_Presat = FALSE
Initial_Wait = 1[s]
abundance
X : parts per Million : 1H
2.00
2.00
3.00
3.00
7.706
7.684
7.423
7.402
7.270
2.955
2.402
1.662

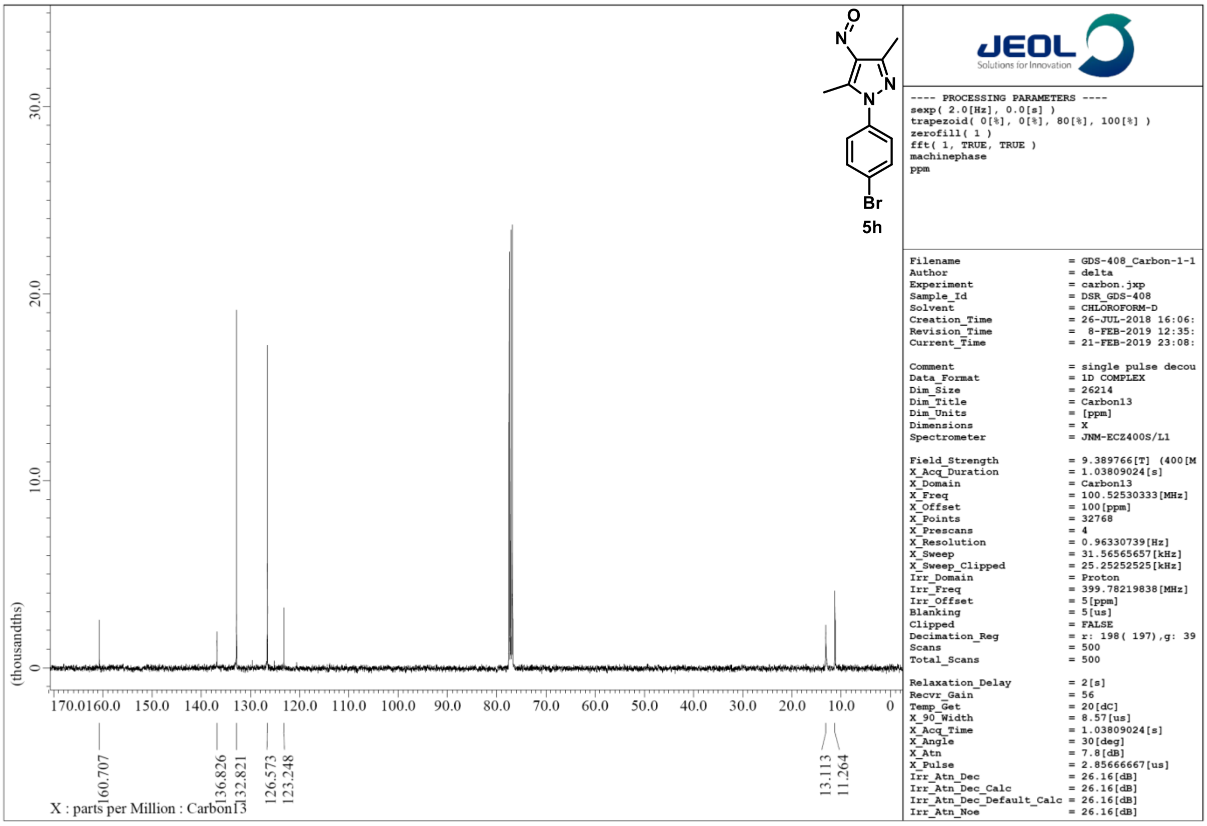
5h
Br
N
N
N
O
JEOL
Solutions for Innovation
---- PROCESSING PARAMETERS ----
sexp(2.0[Hz], 0.0[s])
trapezoid(0[%], 0[%], 80[%], 100[%])
zerofill(1)
fft(1, TRUE, TRUE)
machinephase
ppm
Filename = GDS-408_Carbon-1-1
Author = delta
Experiment = carbon.jxp
Sample_Id = DSR_GDS-408
Solvent = CHLOROFORM-D
Creation_Time = 26-JUL-2018 16:06:
Revision_Time = 8-FEB-2019 12:35:
Current_Time = 21-FEB-2019 23:08:
Comment = single pulse decou
Data_Format = 1D COMPLEX
Dim_Size = 26214
Dim_Title = Carbon13
Dim_Units = [ppm]
Dimensions = X
Spectrometer = JNM-ECZ400S/L1
Field_Strength = 9.389766[T] (400[M
X_Acq_Duration = 1.03809024[s]
X_Domain = Carbon13
X_Freq = 100.52530333[MHz]
X_Offset = 100[ppm]
X_Points = 32768
X_Prescans = 4
X_Resolution = 0.96330739[Hz]
X_Sweep = 31.56565657[kHz]
X_Sweep_Clipped = 25.25252525[kHz]
Irr_Domain = Proton
Irr_Freq = 399.78219838[MHz]
Irr_Offset = 5[ppm]
Blanking = 5[us]
Clipped = FALSE
Decimation_Reg = r: 198(197),g: 39
Scans = 500
Total_Scans = 500
Relaxation_Delay = 2[s]
Recvr_Gain = 56
Temp_Get = 20[dC]
X_90_Width = 8.57[us]
X_Acq_Time = 1.03809024[s]
X_Angle = 30[deg]
X_Atn = 7.8[dB]
X_Pulse = 2.85666667[us]
Irr_Atn_Dec = 26.16[dB]
Irr_Atn_Dec_Calc = 26.16[dB]
Irr_Atn_Dec_Default_Calc = 26.16[dB]
Irr_Atn_Noe = 26.16[dB]
(thousandths)
X : parts per Million : Carbon13
160.707
136.826
132.821
126.573
123.248
13.113
11.264

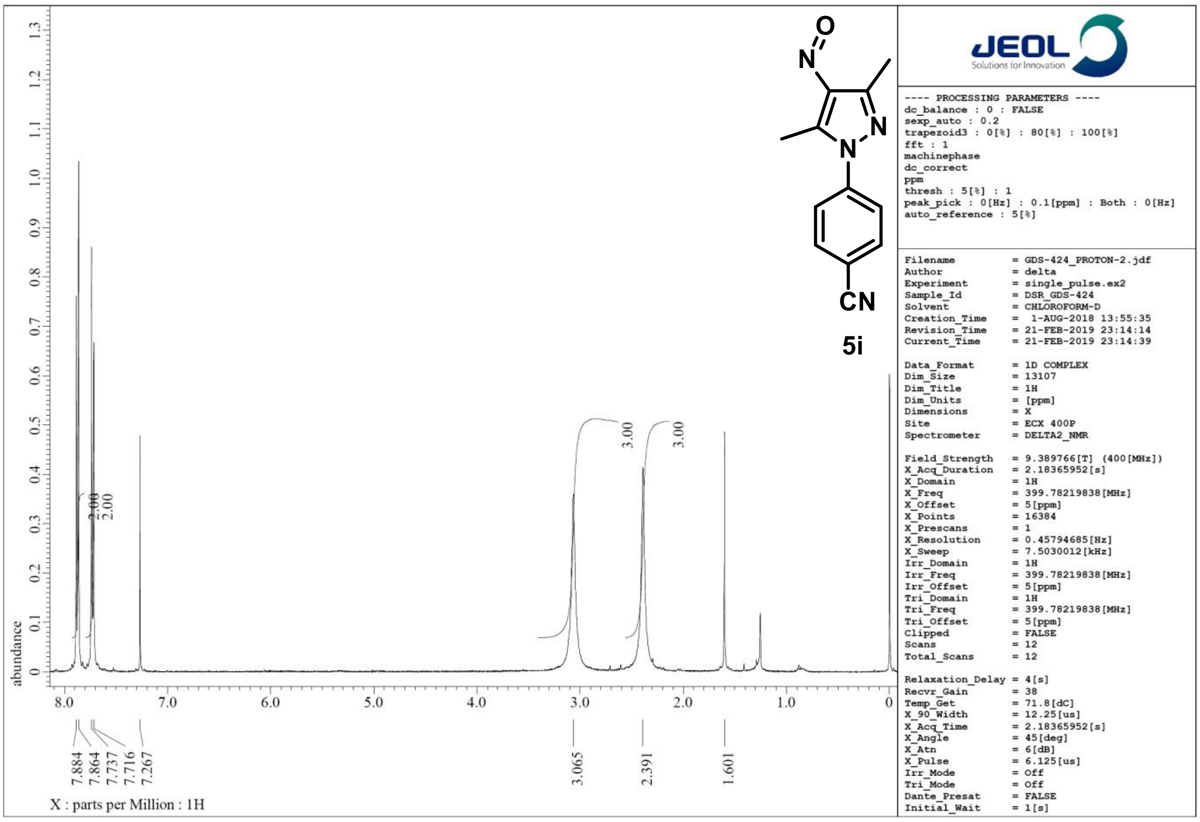
5i
CN
JEOL
Solutions for Innovation
---- PROCESSING PARAMETERS ----
dc_balance : 0 : FALSE
sexp_auto : 0.2
trapezoid3 : 0[%] : 80[%] : 100[%]
fft : 1
machinephase
dc_correct
ppm
thresh : 5[%] : 1
peak_pick : 0[Hz] : 0.1[ppm] : Both : 0[Hz]
auto_reference : 5[%]
Filename = GDS-424_PROTON-2.jdf
Author = delta
Experiment = single_pulse.ex2
Sample_Id = DSR_GDS-424
Solvent = CHLOROFORM-D
Creation_Time = 1-AUG-2018 13:55:35
Revision_Time = 21-FEB-2019 23:14:14
Current_Time = 21-FEB-2019 23:14:39
Data_Format = 1D COMPLEX
Dim_Size = 13107
Dim_Title = 1H
Dim_Units = [ppm]
Dimensions = X
Site = ECX 400P
Spectrometer = DELTA2_NMR
Field_Strength = 9.389766[T] (400[MHz])
X_Acq_Duration = 2.18365952[s]
X_Domain = 1H
X_Freq = 399.78219838[MHz]
X_Offset = 5[ppm]
X_Points = 16384
X_Prescans = 1
X_Resolution = 0.45794685[Hz]
X_Sweep = 7.5030012[kHz]
Irr_Domain = 1H
Irr_Freq = 399.78219838[MHz]
Irr_Offset = 5[ppm]
Tri_Domain = 1H
Tri_Freq = 399.78219838[MHz]
Tri_Offset = 5[ppm]
Clipped = FALSE
Scans = 12
Total_Scans = 12
Relaxation_Delay = 4[s]
Recvr_Gain = 38
Temp_Get = 71.8[dC]
X_90_Width = 12.25[us]
X_Acq_Time = 2.18365952[s]
X_Angle = 45[deg]
X_Atn = 6[dB]
X_Pulse = 6.125[us]
Irr_Mode = Off
Tri_Mode = Off
Dante_Presat = FALSE
Initial_Wait = 1[s]
abundance
2.00
2.00
3.00
3.00
7.884
7.864
7.737
7.716
7.267
3.065
2.391
1.601
X : parts per Million : 1H

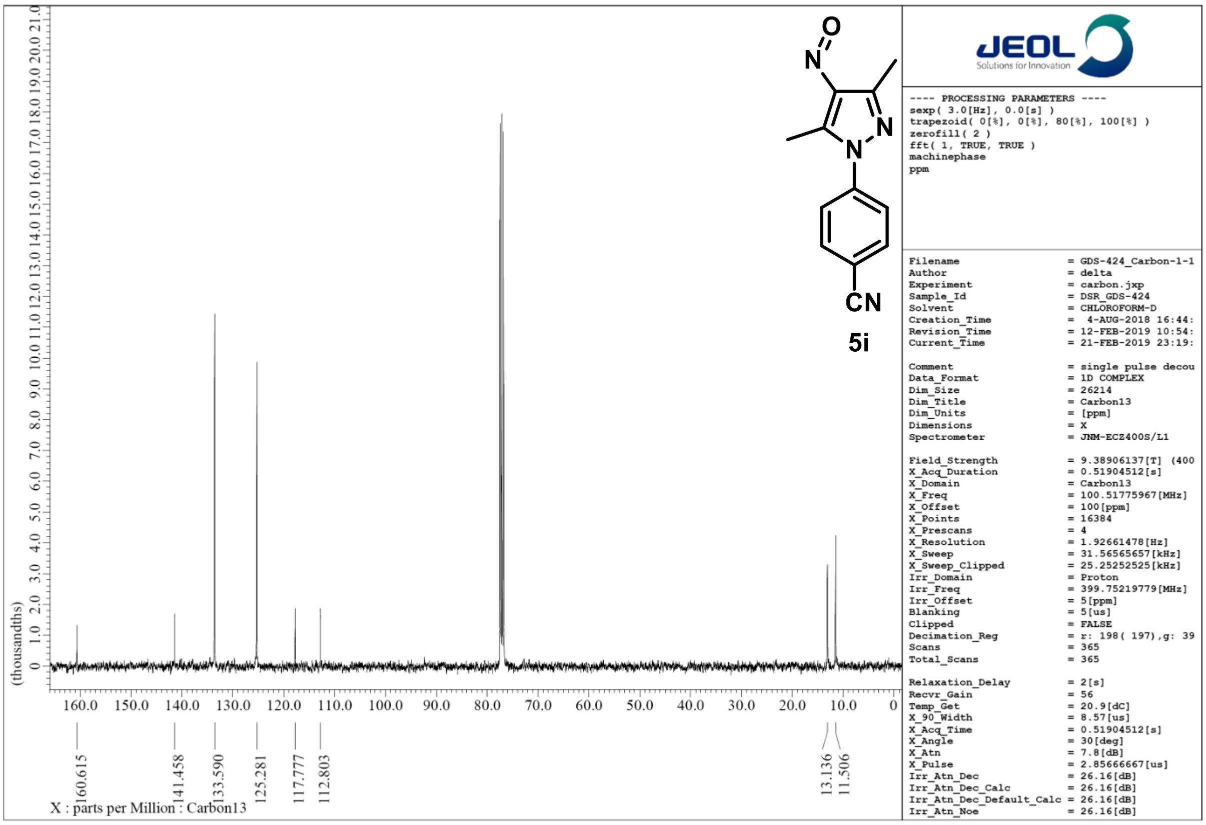
5i
CN
JEOL
Solutions for Innovation
---- PROCESSING PARAMETERS ----
sexp(3.0[Hz], 0.0[s])
trapezoid(0[%], 0[%], 80[%], 100[%])
zerofill(2)
fft(1, TRUE, TRUE)
machinephase
ppm
Filename = GDS-424_Carbon-1-1
Author = delta
Experiment = carbon.jxp
Sample_Id = DSR_GDS-424
Solvent = CHLOROFORM-D
Creation_Time = 4-AUG-2018 16:44:
Revision_Time = 12-FEB-2019 10:54:
Current_Time = 21-FEB-2019 23:19:
Comment = single pulse decou
Data_Format = 1D COMPLEX
Dim_Size = 26214
Dim_Title = Carbon13
Dim_Units = [ppm]
Dimensions = X
Spectrometer = JNM-ECZ400S/L1
Field_Strength = 9.38906137[T] (400
X_Acq_Duration = 0.51904512[s]
X_Domain = Carbon13
X_Freq = 100.51775967[MHz]
X_Offset = 100[ppm]
X_Points = 16384
X_Prescans = 4
X_Resolution = 1.92661478[Hz]
X_Sweep = 31.56565657[kHz]
X_Sweep_Clipped = 25.25252525[kHz]
Irr_Domain = Proton
Irr_Freq = 399.75219779[MHz]
Irr_Offset = 5[ppm]
Blanking = 5[us]
Clipped = FALSE
Decimation_Reg = r: 198(197),g: 39
Scans = 365
Total_Scans = 365
Relaxation_Delay = 2[s]
Recvr_Gain = 56
Temp_Get = 20.9[dC]
X_90_Width = 8.57[us]
X_Acq_Time = 0.51904512[s]
X_Angle = 30[deg]
X_Atn = 7.8[dB]
X_Pulse = 2.85666667[us]
Irr_Atn_Dec = 26.16[dB]
Irr_Atn_Dec_Calc = 26.16[dB]
Irr_Atn_Dec_Default_Calc = 26.16[dB]
Irr_Atn_Noe = 26.16[dB]
(thousandths)
160.615
141.458
133.590
125.281
117.777
112.803
13.136
11.506
X : parts per Million : Carbon13

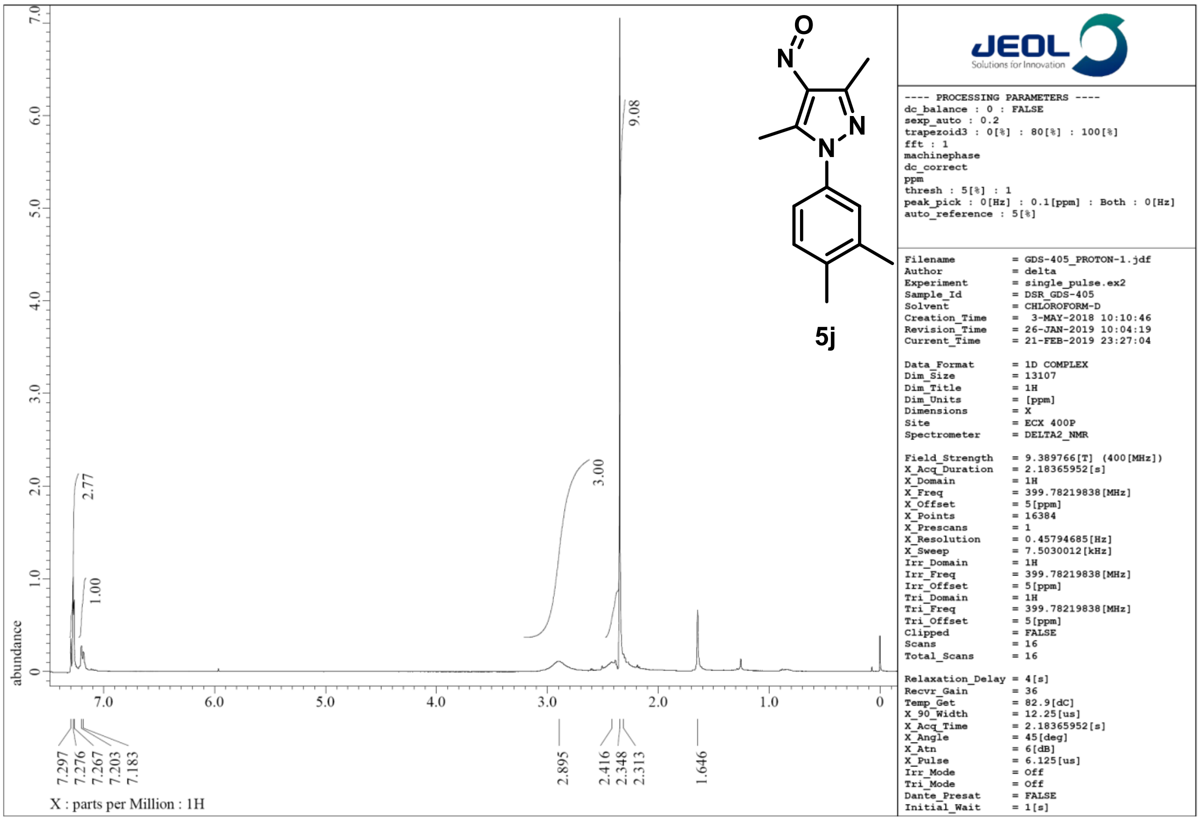
5j
---- PROCESSING PARAMETERS ----
dc_balance : 0 : FALSE
sexp_auto : 0.2
trapezoid3 : 0[%] : 80[%] : 100[%]
fft : 1
machinephase
dc_correct
ppm
thresh : 5[%] : 1
peak_pick : 0[Hz] : 0.1[ppm] : Both : 0[Hz]
auto_reference : 5[%]
Filename = GDS-405_PROTON-1.jdf
Author = delta
Experiment = single_pulse.ex2
Sample_Id = DSR_GDS-405
Solvent = CHLOROFORM-D
Creation_Time = 3-MAY-2018 10:10:46
Revision_Time = 26-JAN-2019 10:04:19
Current_Time = 21-FEB-2019 23:27:04
Data_Format = 1D COMPLEX
Dim_Size = 13107
Dim_Title = 1H
Dim_Units = [ppm]
Dimensions = X
Site = ECX 400P
Spectrometer = DELTA2_NMR
Field_Strength = 9.389766[T] (400[MHz])
X_Acq_Duration = 2.18365952[s]
X_Domain = 1H
X_Freq = 399.78219838[MHz]
X_Offset = 5[ppm]
X_Points = 16384
X_Prescans = 1
X_Resolution = 0.45794685[Hz]
X_Sweep = 7.5030012[kHz]
Irr_Domain = 1H
Irr_Freq = 399.78219838[MHz]
Irr_Offset = 5[ppm]
Tri_Domain = 1H
Tri_Freq = 399.78219838[MHz]
Tri_Offset = 5[ppm]
Clipped = FALSE
Scans = 16
Total_Scans = 16
Relaxation_Delay = 4[s]
Recvr_Gain = 36
Temp_Get = 82.9[dC]
X_90_Width = 12.25[us]
X_Acq_Time = 2.18365952[s]
X_Angle = 45[deg]
X_Atn = 6[dB]
X_Pulse = 6.125[us]
Irr_Mode = Off
Tri_Mode = Off
Dante_Presat = FALSE
Initial_Wait = 1[s]
abundance
X : parts per Million : 1H
2.77
1.00
3.00
9.08
7.297 7.276 7.267 7.203 7.183
2.895
2.416 2.348 2.313
1.646

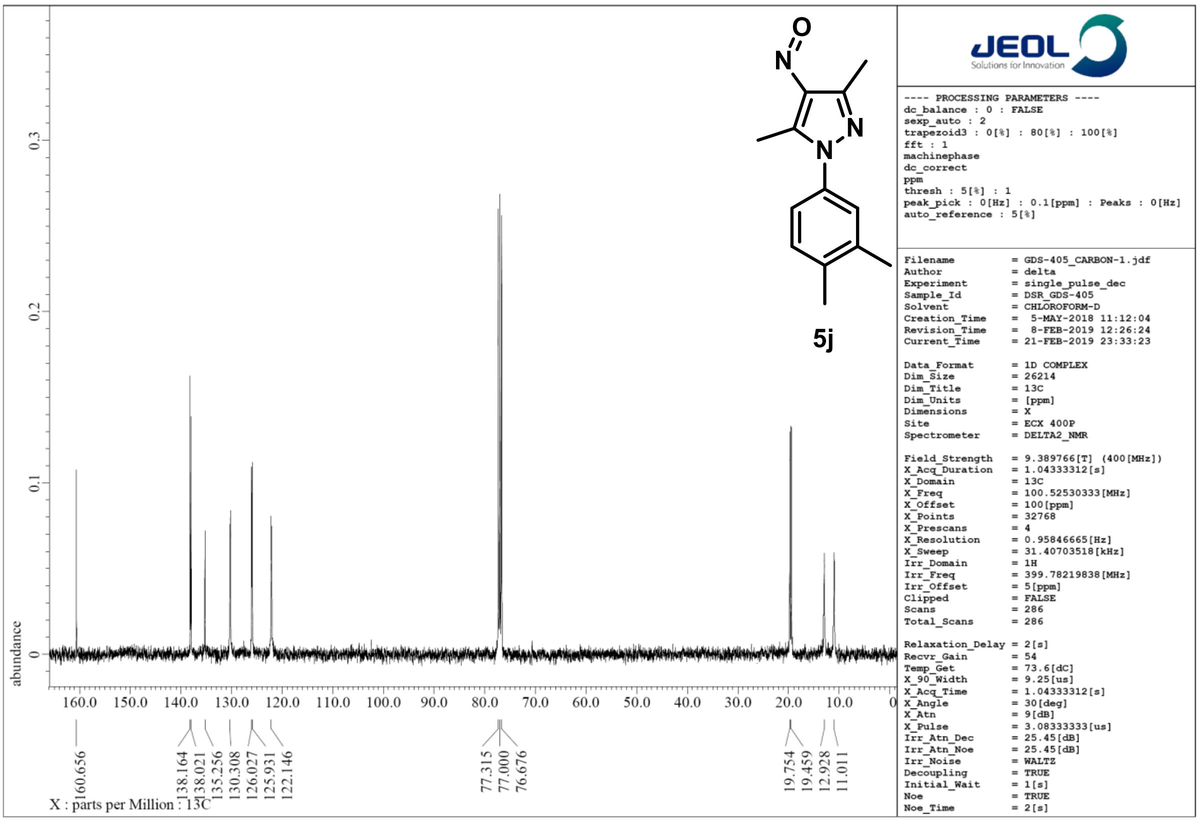
5j
---- PROCESSING PARAMETERS ----
dc_balance : 0 : FALSE
sexp_auto : 2
trapezoid3 : 0[%] : 80[%] : 100[%]
fft : 1
machinephase
dc_correct
ppm
thresh : 5[%] : 1
peak_pick : 0[Hz] : 0.1[ppm] : Peaks : 0[Hz]
auto_reference : 5[%]
Filename = GDS-405_CARBON-1.jdf
Author = delta
Experiment = single_pulse_dec
Sample_Id = DSR_GDS-405
Solvent = CHLOROFORM-D
Creation_Time = 5-MAY-2018 11:12:04
Revision_Time = 8-FEB-2019 12:26:24
Current_Time = 21-FEB-2019 23:33:23
Data_Format = 1D COMPLEX
Dim_Size = 26214
Dim_Title = 13C
Dim_Units = [ppm]
Dimensions = X
Site = ECX 400P
Spectrometer = DELTA2_NMR
Field_Strength = 9.389766[T] (400[MHz])
X_Acq_Duration = 1.04333312[s]
X_Domain = 13C
X_Freq = 100.52530333[MHz]
X_Offset = 100[ppm]
X_Points = 32768
X_Prescans = 4
X_Resolution = 0.95846665[Hz]
X_Sweep = 31.40703518[kHz]
Irr_Domain = 1H
Irr_Freq = 399.78219838[MHz]
Irr_Offset = 5[ppm]
Clipped = FALSE
Scans = 286
Total_Scans = 286
Relaxation_Delay = 2[s]
Recvr_Gain = 54
Temp_Get = 73.6[dC]
X_90_Width = 9.25[us]
X_Acq_Time = 1.04333312[s]
X_Angle = 30[deg]
X_Atn = 9[dB]
X_Pulse = 3.08333333[us]
Irr_Atn_Dec = 25.45[dB]
Irr_Atn_Noe = 25.45[dB]
Irr_Noise = WALTZ
Decoupling = TRUE
Initial_Wait = 1[s]
Noe = TRUE
Noe_Time = 2[s]
abundance
X : parts per Million : 13C
160.656
138.164 138.021 135.256 130.308 126.027 125.931 122.146
77.315 77.000 76.676
19.754 19.459 12.928 11.011

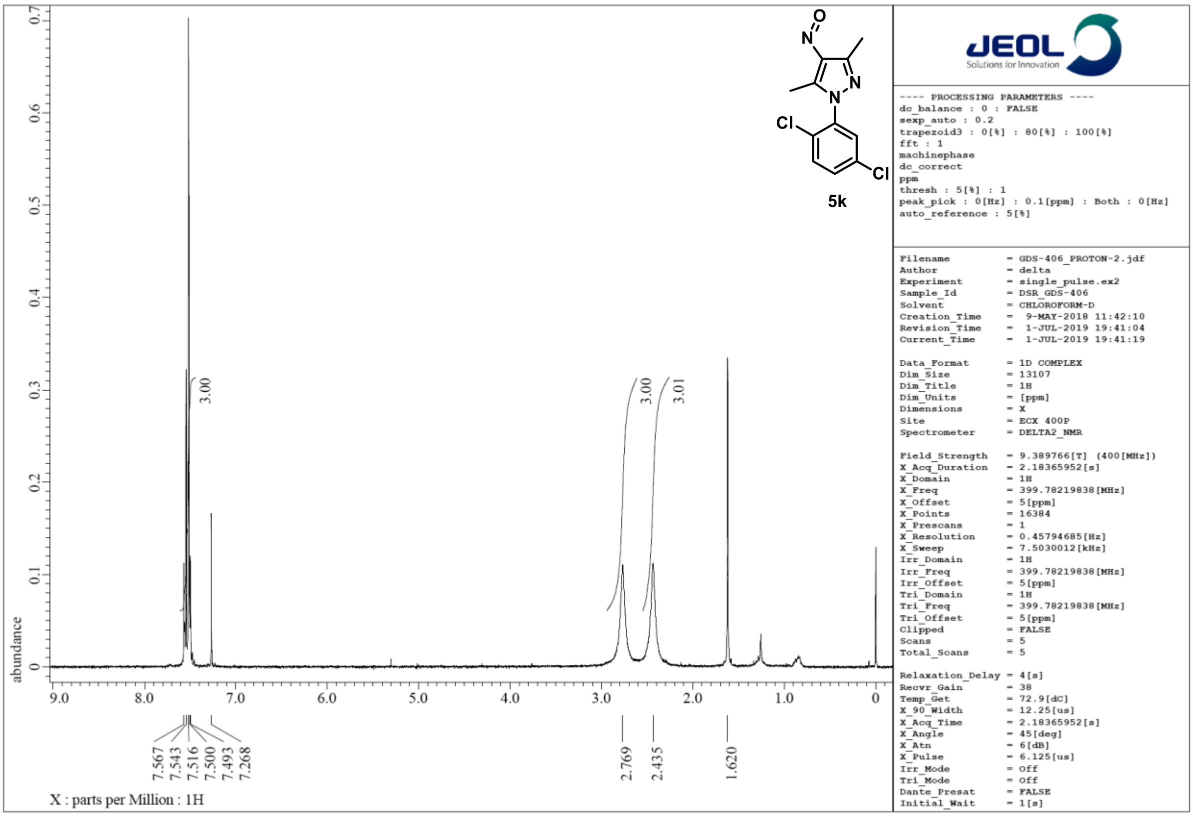
5k
Cl
Cl
N
N
N
O
JEOL
Solutions for Innovation
---- PROCESSING PARAMETERS ----
dc_balance : 0 : FALSE
sexp_auto : 0.2
trapezoid3 : 0[%] : 80[%] : 100[%]
fft : 1
machinephase
dc_correct
ppm
thresh : 5[%] : 1
peak_pick : 0[Hz] : 0.1[ppm] : Both : 0[Hz]
auto_reference : 5[%]
Filename = GDS-406_PROTON-2.jdf
Author = delta
Experiment = single_pulse.ex2
Sample_Id = DSR_GDS-406
Solvent = CHLOROFORM-D
Creation_Time = 9-MAY-2018 11:42:10
Revision_Time = 1-JUL-2019 19:41:04
Current_Time = 1-JUL-2019 19:41:19
Data_Format = 1D COMPLEX
Dim_Size = 13107
Dim_Title = 1H
Dim_Units = [ppm]
Dimensions = X
Site = ECX 400P
Spectrometer = DELTA2_NMR
Field_Strength = 9.389766[T] (400[MHz])
X_Acq_Duration = 2.18365952[s]
X_Domain = 1H
X_Freq = 399.78219838[MHz]
X_Offset = 5[ppm]
X_Points = 16384
X_Prescans = 1
X_Resolution = 0.45794685[Hz]
X_Sweep = 7.5030012[kHz]
Irr_Domain = 1H
Irr_Freq = 399.78219838[MHz]
Irr_Offset = 5[ppm]
Tri_Domain = 1H
Tri_Freq = 399.78219838[MHz]
Tri_Offset = 5[ppm]
Clipped = FALSE
Scans = 5
Total_Scans = 5
Relaxation_Delay = 4[s]
Recvr_Gain = 38
Temp_Get = 72.9[dC]
X_90_Width = 12.25[us]
X_Acq_Time = 2.18365952[s]
X_Angle = 45[deg]
X_Atn = 6[dB]
X_Pulse = 6.125[us]
Irr_Mode = Off
Tri_Mode = Off
Dante_Presat = FALSE
Initial_Wait = 1[s]
abundance
0 0.1 0.2 0.3 0.4 0.5 0.6 0.7
3.00 3.00 3.01
9.0 8.0 7.0 6.0 5.0 4.0 3.0 2.0 1.0 0
7.567 7.543 7.516 7.500 7.493 7.268
2.769 2.435 1.620
X : parts per Million : 1H

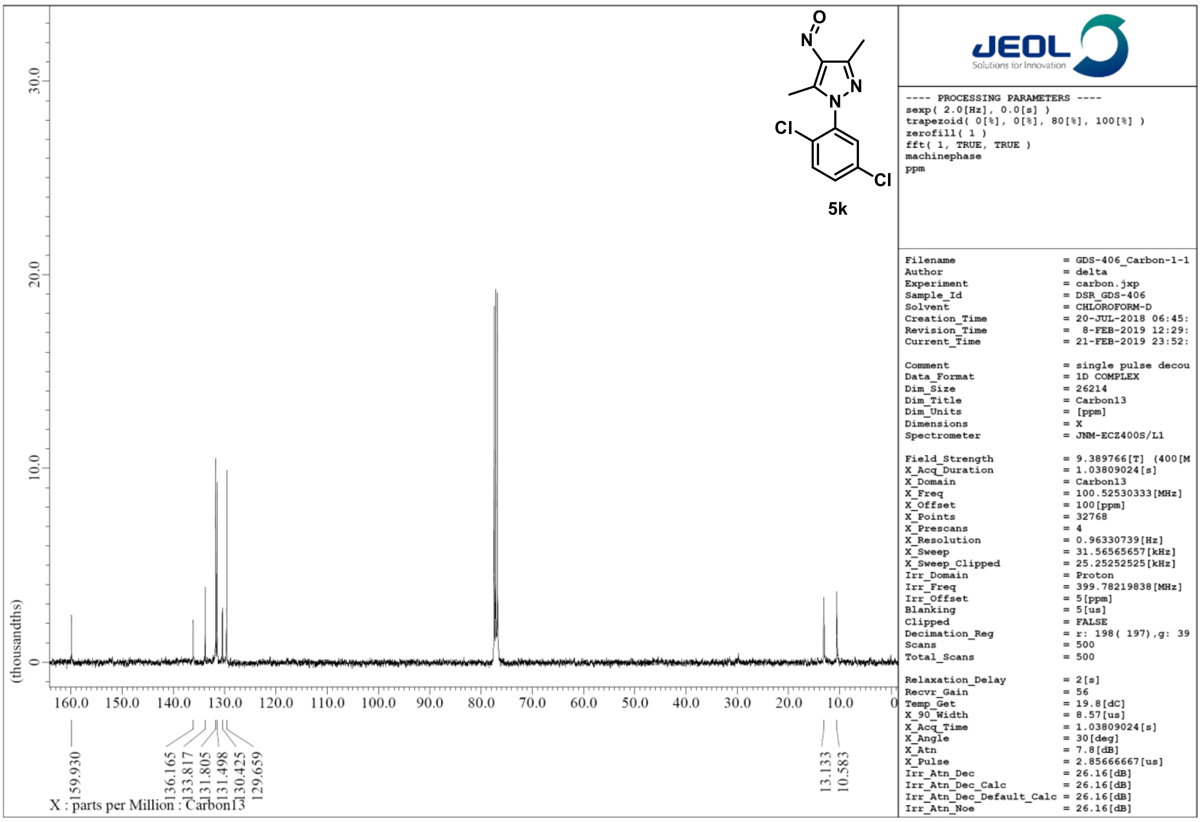
5k
Cl
Cl
N
N
N
O
JEOL
Solutions for Innovation
---- PROCESSING PARAMETERS ----
sexp(2.0[Hz], 0.0[s])
trapezoid(0[%], 0[%], 80[%], 100[%])
zerofill(1)
fft(1, TRUE, TRUE)
machinephase
ppm
Filename = GDS-406_Carbon-1-1
Author = delta
Experiment = carbon.jxp
Sample_Id = DSR_GDS-406
Solvent = CHLOROFORM-D
Creation_Time = 20-JUL-2018 06:45:
Revision_Time = 8-FEB-2019 12:29:
Current_Time = 21-FEB-2019 23:52:
Comment = single pulse decou
Data_Format = 1D COMPLEX
Dim_Size = 26214
Dim_Title = Carbon13
Dim_Units = [ppm]
Dimensions = X
Spectrometer = JNM-ECZ400S/L1
Field_Strength = 9.389766[T] (400[M
X_Acq_Duration = 1.03809024[s]
X_Domain = Carbon13
X_Freq = 100.52530333[MHz]
X_Offset = 100[ppm]
X_Points = 32768
X_Prescans = 4
X_Resolution = 0.96330739[Hz]
X_Sweep = 31.56565657[kHz]
X_Sweep_Clipped = 25.25252525[kHz]
Irr_Domain = Proton
Irr_Freq = 399.78219838[MHz]
Irr_Offset = 5[ppm]
Blanking = 5[us]
Clipped = FALSE
Decimation_Reg = r: 198(197),g: 39
Scans = 500
Total_Scans = 500
Relaxation_Delay = 2[s]
Recvr_Gain = 56
Temp_Get = 19.8[dC]
X_90_Width = 8.57[us]
X_Acq_Time = 1.03809024[s]
X_Angle = 30[deg]
X_Atn = 7.8[dB]
X_Pulse = 2.85666667[us]
Irr_Atn_Dec = 26.16[dB]
Irr_Atn_Dec_Calc = 26.16[dB]
Irr_Atn_Dec_Default_Calc = 26.16[dB]
Irr_Atn_Noe = 26.16[dB]
(thousandths)
0 10.0 20.0 30.0
160.0 150.0 140.0 130.0 120.0 110.0 100.0 90.0 80.0 70.0 60.0 50.0 40.0 30.0 20.0 10.0 0
159.930
136.165 133.817 131.805 131.498 130.425 129.659
13.133 10.583
X : parts per Million : Carbon13

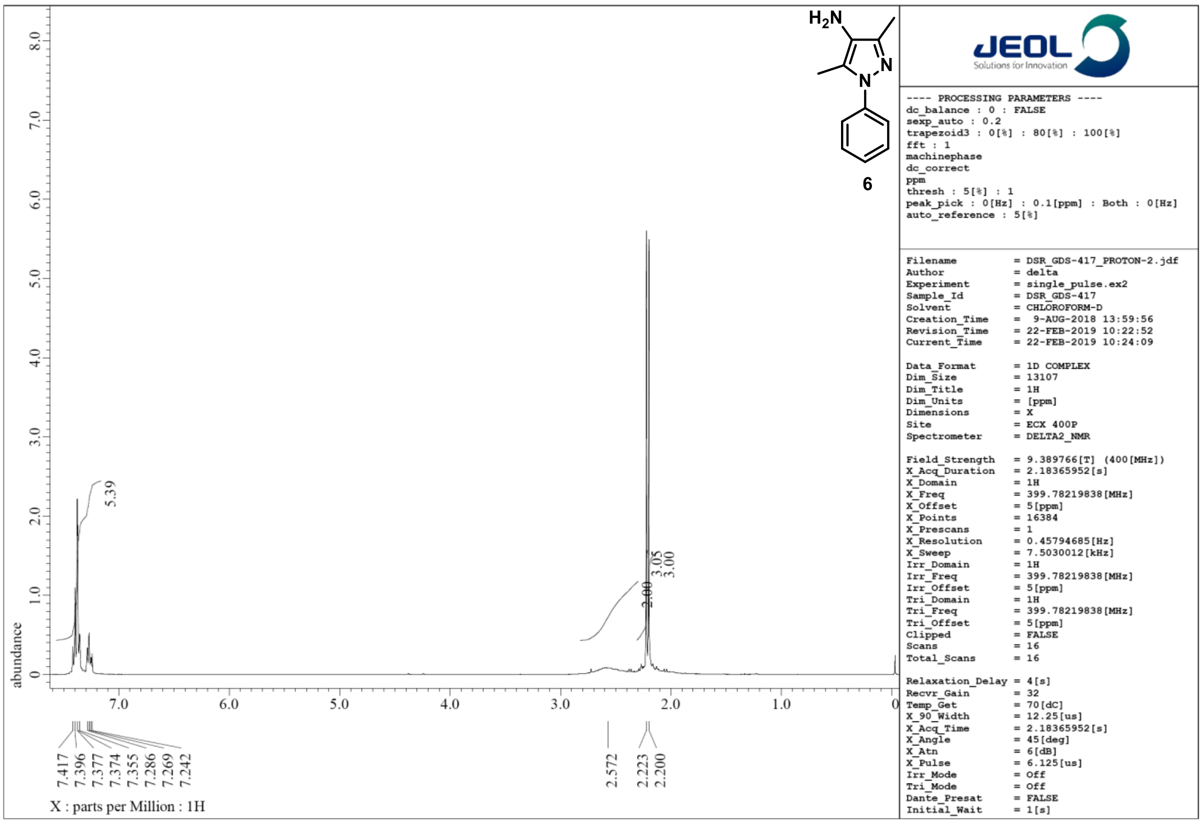

H2N
N
N
6
JEOL
Solutions for Innovation
---- PROCESSING PARAMETERS ----
dc_balance : 0 : FALSE
sexp_auto : 0.2
trapezoid3 : 0[%] : 80[%] : 100[%]
fft : 1
machinephase
dc_correct
ppm
thresh : 5[%] : 1
peak_pick : 0[Hz] : 0.1[ppm] : Both : 0[Hz]
auto_reference : 5[%]
Filename = DSR_GDS-417_PROTON-2.jdf
Author = delta
Experiment = single_pulse.ex2
Sample_Id = DSR_GDS-417
Solvent = CHLOROFORM-D
Creation_Time = 9-AUG-2018 13:59:56
Revision_Time = 22-FEB-2019 10:22:52
Current_Time = 22-FEB-2019 10:24:09
Data_Format = 1D COMPLEX
Dim_Size = 13107
Dim_Title = 1H
Dim_Units = [ppm]
Dimensions = X
Site = ECX 400P
Spectrometer = DELTA2_NMR
Field_Strength = 9.389766[T] (400[MHz])
X_Acq_Duration = 2.18365952[s]
X_Domain = 1H
X_Freq = 399.78219838[MHz]
X_Offset = 5[ppm]
X_Points = 16384
X_Prescans = 1
X_Resolution = 0.45794685[Hz]
X_Sweep = 7.5030012[kHz]
Irr_Domain = 1H
Irr_Freq = 399.78219838[MHz]
Irr_Offset = 5[ppm]
Tri_Domain = 1H
Tri_Freq = 399.78219838[MHz]
Tri_Offset = 5[ppm]
Clipped = FALSE
Scans = 16
Total_Scans = 16
Relaxation_Delay = 4[s]
Recvr_Gain = 32
Temp_Get = 70[dC]
X_90_Width = 12.25[us]
X_Acq_Time = 2.18365952[s]
X_Angle = 45[deg]
X_Atn = 6[dB]
X_Pulse = 6.125[us]
Irr_Mode = Off
Tri_Mode = Off
Dante_Presat = FALSE
Initial_Wait = 1[s]
abundance
8.0
7.0
6.0
5.0
4.0
3.0
2.0
1.0
0
5.39
2.00
3.05
3.00
7.0
6.0
5.0
4.0
3.0
2.0
1.0
0
7.417
7.396
7.377
7.374
7.355
7.286
7.269
7.242
2.572
2.223
2.200
X : parts per Million : 1H

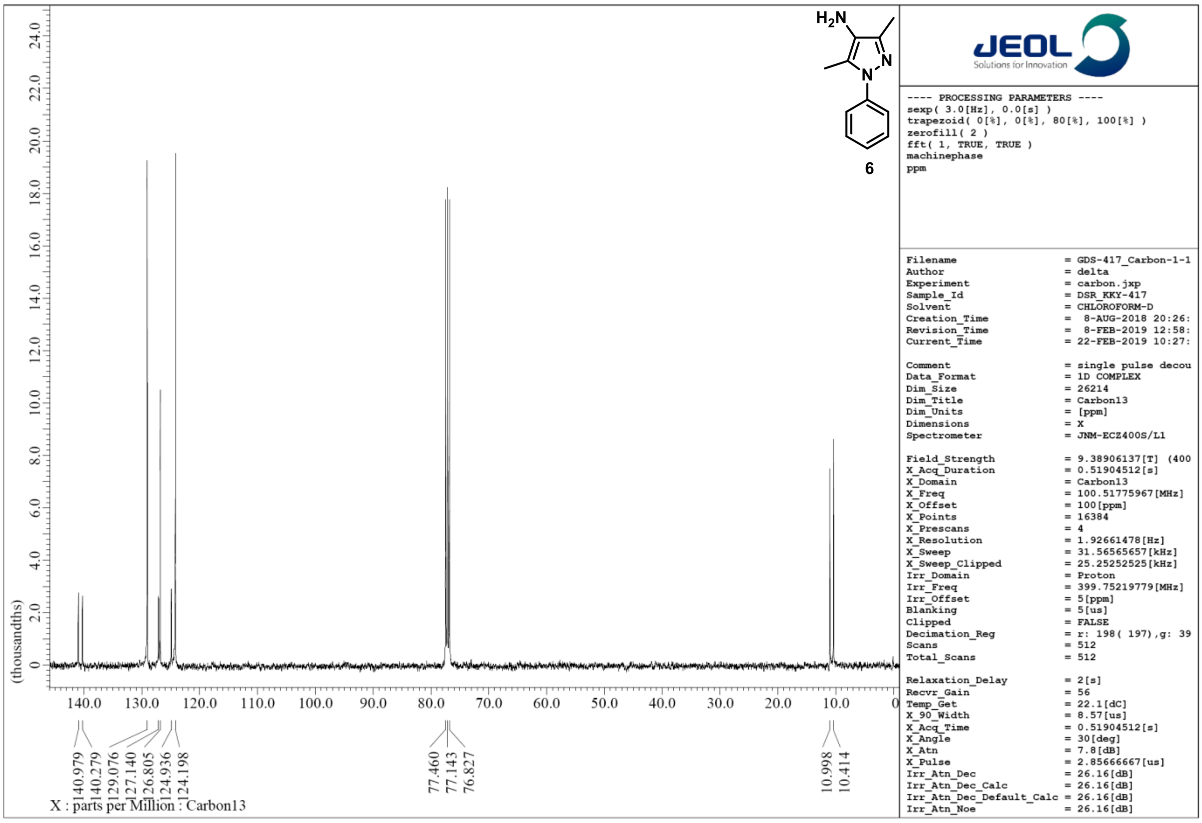

H2N
N
N
6
JEOL
Solutions for Innovation
---- PROCESSING PARAMETERS ----
sexp(3.0[Hz], 0.0[s])
trapezoid(0[%], 0[%], 80[%], 100[%])
zerofill(2)
fft(1, TRUE, TRUE)
machinephase
ppm
Filename = GDS-417_Carbon-1-1
Author = delta
Experiment = carbon.jxp
Sample_Id = DSR_KKY-417
Solvent = CHLOROFORM-D
Creation_Time = 8-AUG-2018 20:26:
Revision_Time = 8-FEB-2019 12:58:
Current_Time = 22-FEB-2019 10:27:
Comment = single pulse decou
Data_Format = 1D COMPLEX
Dim_Size = 26214
Dim_Title = Carbon13
Dim_Units = [ppm]
Dimensions = X
Spectrometer = JNM-ECZ400S/L1
Field_Strength = 9.38906137[T] (400
X_Acq_Duration = 0.51904512[s]
X_Domain = Carbon13
X_Freq = 100.51775967[MHz]
X_Offset = 100[ppm]
X_Points = 16384
X_Prescans = 4
X_Resolution = 1.92661478[Hz]
X_Sweep = 31.56565657[kHz]
X_Sweep_Clipped = 25.25252525[kHz]
Irr_Domain = Proton
Irr_Freq = 399.75219779[MHz]
Irr_Offset = 5[ppm]
Blanking = 5[us]
Clipped = FALSE
Decimation_Reg = r: 198(197),g: 39
Scans = 512
Total_Scans = 512
Relaxation_Delay = 2[s]
Recvr_Gain = 56
Temp_Get = 22.1[dC]
X_90_Width = 8.57[us]
X_Acq_Time = 0.51904512[s]
X_Angle = 30[deg]
X_Atn = 7.8[dB]
X_Pulse = 2.85666667[us]
Irr_Atn_Dec = 26.16[dB]
Irr_Atn_Dec_Calc = 26.16[dB]
Irr_Atn_Dec_Default_Calc = 26.16[dB]
Irr_Atn_Noe = 26.16[dB]
(thousandths)
24.0
22.0
20.0
18.0
16.0
14.0
12.0
10.0
8.0
6.0
4.0
2.0
0
140.0
130.0
120.0
110.0
100.0
90.0
80.0
70.0
60.0
50.0
40.0
30.0
20.0
10.0
0
140.979
140.279
129.076
127.140
126.805
124.936
124.198
77.460
77.143
76.827
10.998
10.414
X : parts per Million : Carbon13

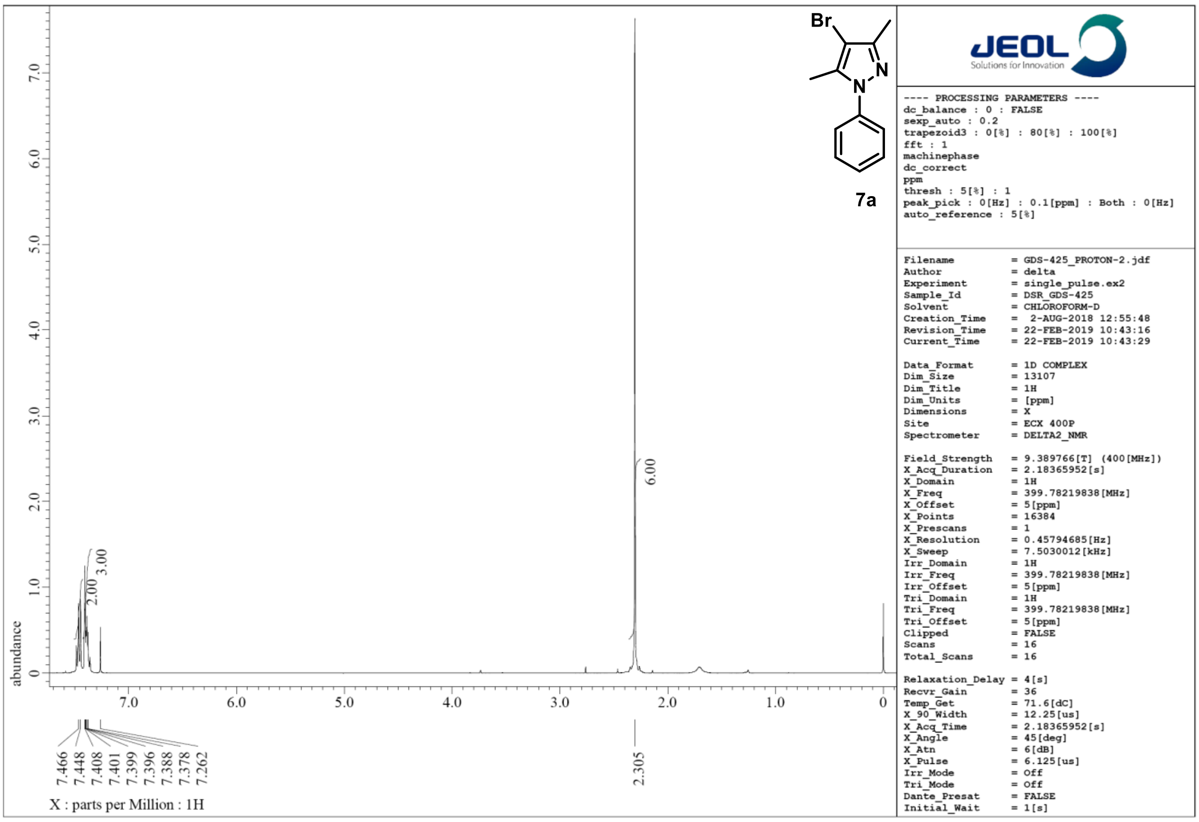
7a
JEOL
Solutions for Innovation
---- PROCESSING PARAMETERS ----
dc_balance : 0 : FALSE
sexp_auto : 0.2
trapezoid3 : 0[%] : 80[%] : 100[%]
fft : 1
machinephase
dc_correct
ppm
thresh : 5[%] : 1
peak_pick : 0[Hz] : 0.1[ppm] : Both : 0[Hz]
auto_reference : 5[%]
Filename = GDS-425_PROTON-2.jdf
Author = delta
Experiment = single_pulse.ex2
Sample_Id = DSR_GDS-425
Solvent = CHLOROFORM-D
Creation_Time = 2-AUG-2018 12:55:48
Revision_Time = 22-FEB-2019 10:43:16
Current_Time = 22-FEB-2019 10:43:29
Data_Format = 1D COMPLEX
Dim_Size = 13107
Dim_Title = 1H
Dim_Units = [ppm]
Dimensions = X
Site = ECX 400P
Spectrometer = DELTA2_NMR
Field_Strength = 9.389766[T] (400[MHz])
X_Acq_Duration = 2.18365952[s]
X_Domain = 1H
X_Freq = 399.78219838[MHz]
X_Offset = 5[ppm]
X_Points = 16384
X_Prescans = 1
X_Resolution = 0.45794685[Hz]
X_Sweep = 7.5030012[kHz]
Irr_Domain = 1H
Irr_Freq = 399.78219838[MHz]
Irr_Offset = 5[ppm]
Tri_Domain = 1H
Tri_Freq = 399.78219838[MHz]
Tri_Offset = 5[ppm]
Clipped = FALSE
Scans = 16
Total_Scans = 16
Relaxation_Delay = 4[s]
Recvr_Gain = 36
Temp_Get = 71.6[dC]
X_90_Width = 12.25[us]
X_Acq_Time = 2.18365952[s]
X_Angle = 45[deg]
X_Atn = 6[dB]
X_Pulse = 6.125[us]
Irr_Mode = Off
Tri_Mode = Off
Dante_Presat = FALSE
Initial_Wait = 1[s]
abundance
3.00
2.00
6.00
7.466 7.448 7.408 7.401 7.399 7.396 7.388 7.378 7.262
2.305
X : parts per Million : 1H

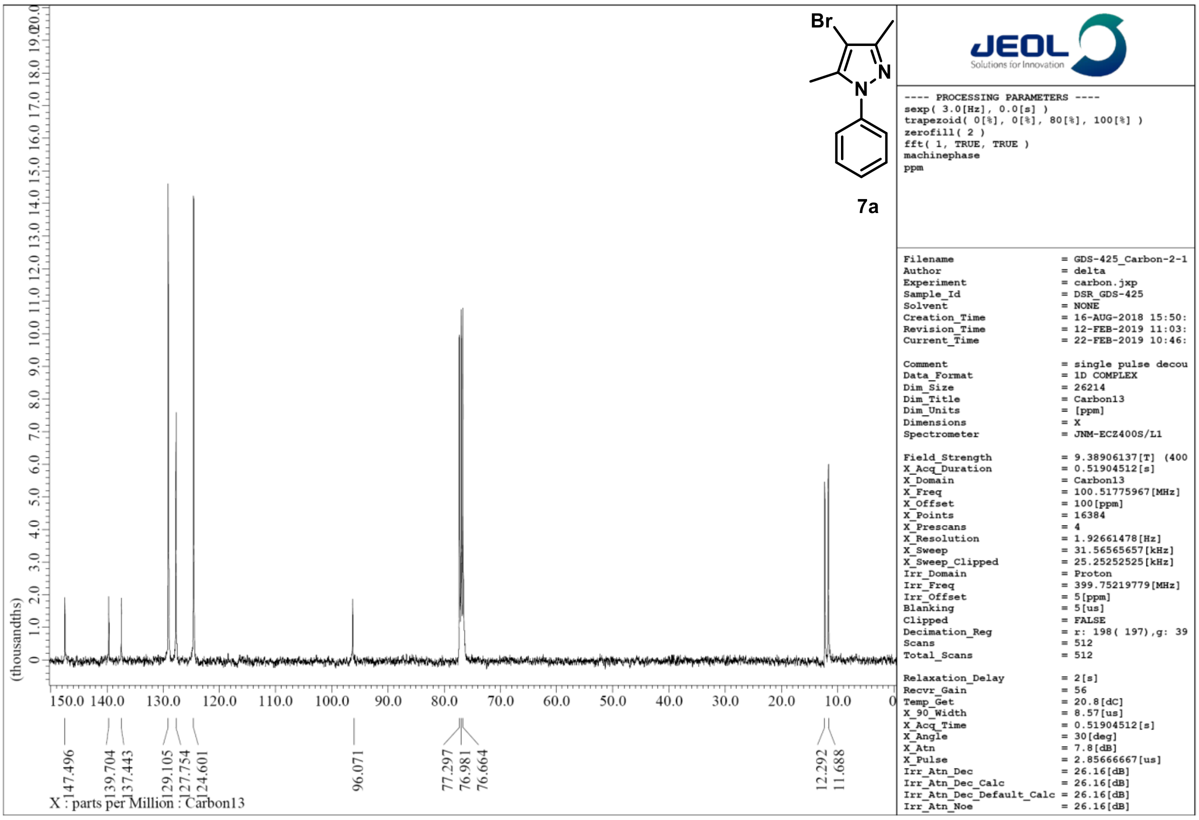
7a
JEOL
Solutions for Innovation
---- PROCESSING PARAMETERS ----
sexp(3.0[Hz], 0.0[s])
trapezoid(0[%], 0[%], 80[%], 100[%])
zerofill(2)
fft(1, TRUE, TRUE)
machinephase
ppm
Filename = GDS-425_Carbon-2-1
Author = delta
Experiment = carbon.jxp
Sample_Id = DSR_GDS-425
Solvent = NONE
Creation_Time = 16-AUG-2018 15:50:
Revision_Time = 12-FEB-2019 11:03:
Current_Time = 22-FEB-2019 10:46:
Comment = single pulse decou
Data_Format = 1D COMPLEX
Dim_Size = 26214
Dim_Title = Carbon13
Dim_Units = [ppm]
Dimensions = X
Spectrometer = JNM-ECZ400S/L1
Field_Strength = 9.38906137[T] (400
X_Acq_Duration = 0.51904512[s]
X_Domain = Carbon13
X_Freq = 100.51775967[MHz]
X_Offset = 100[ppm]
X_Points = 16384
X_Prescans = 4
X_Resolution = 1.92661478[Hz]
X_Sweep = 31.56565657[kHz]
X_Sweep_Clipped = 25.25252525[kHz]
Irr_Domain = Proton
Irr_Freq = 399.75219779[MHz]
Irr_Offset = 5[ppm]
Blanking = 5[us]
Clipped = FALSE
Decimation_Reg = r: 198(197),g: 39
Scans = 512
Total_Scans = 512
Relaxation_Delay = 2[s]
Recvr_Gain = 56
Temp_Get = 20.8[dC]
X_90_Width = 8.57[us]
X_Acq_Time = 0.51904512[s]
X_Angle = 30[deg]
X_Atn = 7.8[dB]
X_Pulse = 2.85666667[us]
Irr_Atn_Dec = 26.16[dB]
Irr_Atn_Dec_Calc = 26.16[dB]
Irr_Atn_Dec_Default_Calc = 26.16[dB]
Irr_Atn_Noe = 26.16[dB]
(thousandths)
147.496 139.704 137.443 129.105 127.754 124.601 96.071 77.297 76.981 76.664 12.292 11.688
X : parts per Million : Carbon13

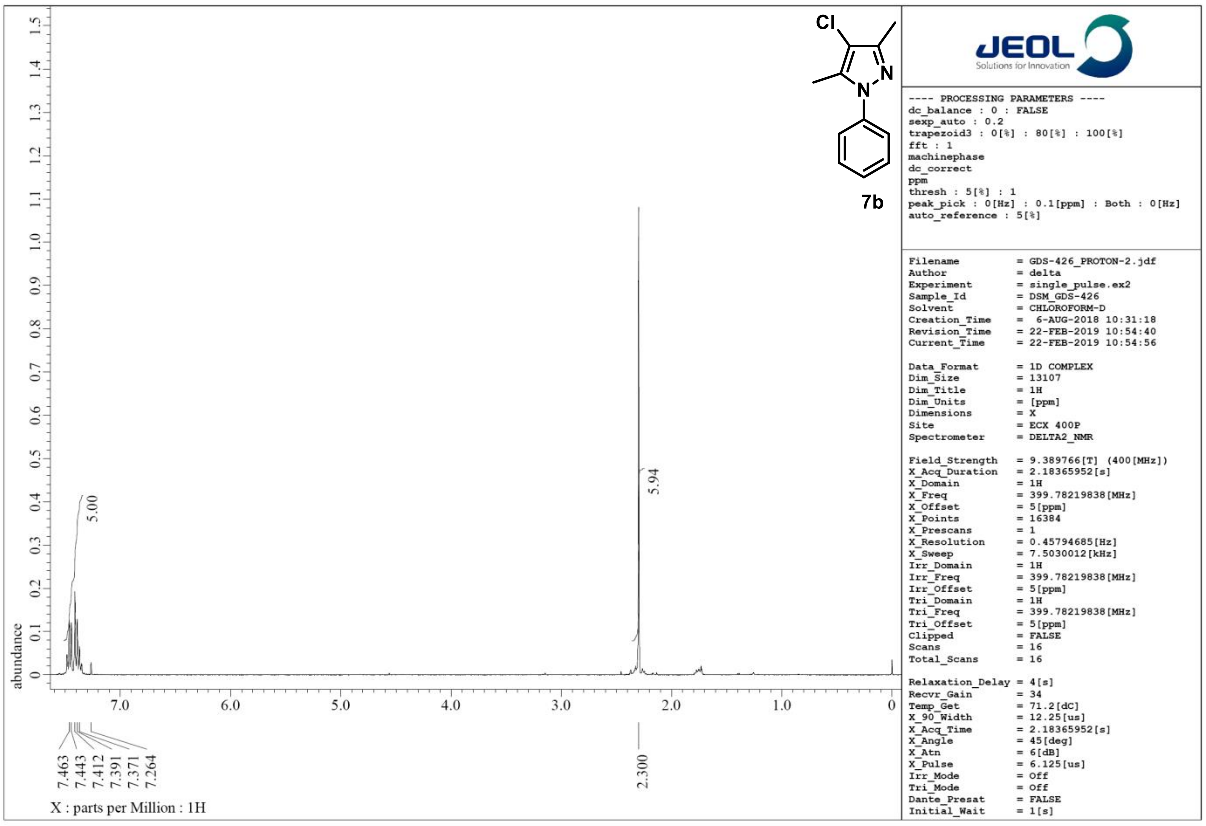

JEOL Solutions for Innovation

```
---- PROCESSING PARAMETERS ----
dc_balance : 0 : FALSE
sexp_auto : 0.2
trapezoid3 : 0[%] : 80[%] : 100[%]
fft : 1
machinephase
dc_correct
ppm
thresh : 5[%] : 1
peak_pick : 0[Hz] : 0.1[ppm] : Both : 0[Hz]
auto_reference : 5[%]

Filename         = GDS-426_PROTON-2.jdf
Author           = delta
Experiment       = single_pulse.ex2
Sample_Id        = DSM_GDS-426
Solvent          = CHLOROFORM-D
Creation_Time    =  6-AUG-2018 10:31:18
Revision_Time    = 22-FEB-2019 10:54:40
Current_Time     = 22-FEB-2019 10:54:56

Data_Format      = 1D COMPLEX
Dim_Size         = 13107
Dim_Title        = 1H
Dim_Units        = [ppm]
Dimensions       = X
Site             = ECX 400P
Spectrometer     = DELTA2_NMR

Field_Strength   = 9.389766[T] (400[MHz])
X_Acq_Duration   = 2.18365952[s]
X_Domain         = 1H
X_Freq           = 399.78219838[MHz]
X_Offset         = 5[ppm]
X_Points         = 16384
X_Prescans       = 1
X_Resolution     = 0.45794685[Hz]
X_Sweep          = 7.5030012[kHz]
Irr_Domain       = 1H
Irr_Freq         = 399.78219838[MHz]
Irr_Offset       = 5[ppm]
Tri_Domain       = 1H
Tri_Freq         = 399.78219838[MHz]
Tri_Offset       = 5[ppm]
Clipped          = FALSE
Scans            = 16
Total_Scans      = 16

Relaxation_Delay = 4[s]
Recvr_Gain       = 34
Temp_Get         = 71.2[dC]
X_90_Width       = 12.25[us]
X_Acq_Time       = 2.18365952[s]
X_Angle          = 45[deg]
X_Atn            = 6[dB]
X_Pulse          = 6.125[us]
Irr_Mode         = Off
Tri_Mode         = Off
Dante_Presat     = FALSE
Initial_Wait     = 1[s]
```

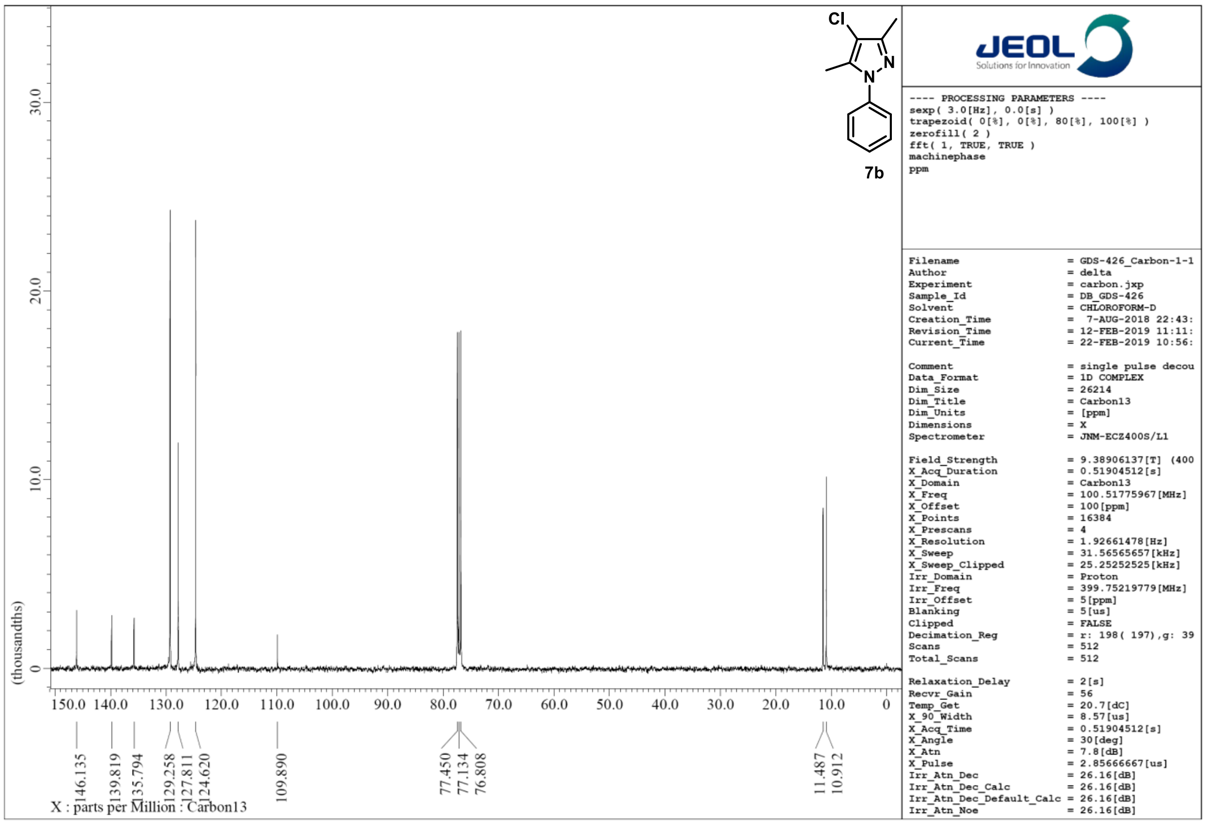

JEOL Solutions for Innovation

```
---- PROCESSING PARAMETERS ----
sexp( 3.0[Hz], 0.0[s] )
trapezoid( 0[%], 0[%], 80[%], 100[%] )
zerofill( 2 )
fft( 1, TRUE, TRUE )
machinephase
ppm

Filename                 = GDS-426_Carbon-1-1
Author                   = delta
Experiment               = carbon.jxp
Sample_Id                = DB_GDS-426
Solvent                  = CHLOROFORM-D
Creation_Time            =  7-AUG-2018 22:43:
Revision_Time            = 12-FEB-2019 11:11:
Current_Time             = 22-FEB-2019 10:56:

Comment                  = single pulse decou
Data_Format              = 1D COMPLEX
Dim_Size                 = 26214
Dim_Title                = Carbon13
Dim_Units                = [ppm]
Dimensions               = X
Spectrometer             = JNM-ECZ400S/L1

Field_Strength           = 9.38906137[T] (400
X_Acq_Duration           = 0.51904512[s]
X_Domain                 = Carbon13
X_Freq                   = 100.51775967[MHz]
X_Offset                 = 100[ppm]
X_Points                 = 16384
X_Prescans               = 4
X_Resolution             = 1.92661478[Hz]
X_Sweep                  = 31.56565657[kHz]
X_Sweep_Clipped          = 25.25252525[kHz]
Irr_Domain               = Proton
Irr_Freq                 = 399.75219779[MHz]
Irr_Offset               = 5[ppm]
Blanking                 = 5[us]
Clipped                  = FALSE
Decimation_Reg           = r: 198( 197),g: 39
Scans                    = 512
Total_Scans              = 512

Relaxation_Delay         = 2[s]
Recvr_Gain               = 56
Temp_Get                 = 20.7[dC]
X_90_Width               = 8.57[us]
X_Acq_Time               = 0.51904512[s]
X_Angle                  = 30[deg]
X_Atn                    = 7.8[dB]
X_Pulse                  = 2.85666667[us]
Irr_Atn_Dec              = 26.16[dB]
Irr_Atn_Dec_Calc         = 26.16[dB]
Irr_Atn_Dec_Default_Calc = 26.16[dB]
Irr_Atn_Noe              = 26.16[dB]
```

LEGENDS FOR SUPPLEMENTARY FIGURES

Supplementary Figure 1: This figure shows the chemical structures of shortlisted compounds obtained from phenotypic screens performed in *M. bovis* BCG.

Supplementary Figure 2: Two drug checkerboard assay to study the interaction of NSC 18725 with INH, RIF, EMB, BDQ, BTZ043 or PA-824. Fractional inhibitory concentrations (FICs) and fractional inhibitory concentration index (∑FIC) for various drug combinations were calculated using the following formula: FIC=MIC in combination/MIC alone. ∑FIC = FIC of drug A + FIC of drug B. ∑FIC value ≤ 0.5 indicate synergistic activity, ∑FIC of ≥ 4.0 indicate antagonistic activity, and values in between (≤ 4.0 and > .5) indicate an additive interaction. Wells showing + sign denote growth, - sign denote no growth, and (-) sign denote activity due to a combination. The data shown in this panel is representative of three independent experiments.
